# Supplementary material for: A Systematic Review and Appraisal of Epidemiological Studies on Household Fuel Use and Its Health Effects Using Demographic and Health Surveys
Source: Int J Environ Res Public Health. 2021 Feb 3;18(4):1411. doi: 10.3390/ijerph18041411 (PMC7913474; doi:10.3390/ijerph18041411)
Supplement: Supplementary file 1 [file ijerph-18-01411-s001.zip › Supplementary files/Table S1_Search terms and strategies.docx]

**S1 Table.** Search terms and techniques applied in to each database

| **MeSH search terms** | | | **Word and phrase search terms** | | |
| --- | --- | --- | --- | --- | --- |
| Cooking | | | Indoor cooking | charcoal | Crop waste |
| indoor air pollution | | | Outdoor cooking | coal | Agricultural waste |
| Family characteristics | | | Cooking fuel | Biofuel | Corp residue |
| Health surveys | | | Heating fuel | Dung | Agricultural residue |
| Cross-sectional studies | | | Kitchen | Biomass | Plant residue |
|  | | | House | Biomass fuel | Household characteristics |
|  | | | Fuel | Fossil fuel | Household air pollution |
|  | | | Smoke | Solid fuel | indoor |
|  | | | Wood | Wood smoke | Air pollution |
|  | | | Kerosene |  |  |
| **Searching techniques across databases** | | | | | |
| **PubMed** | cooking[Mesh] OR “indoor cooking”[tiab] OR “outdoor cooking” [tiab] OR “cooking fuel”[tiab] OR “heating fuel”[tiab] OR kitchen[tiab] OR hous*[tiab] OR fuel[tiab] OR smok*[tiab] OR wood[tiab] OR kerosene[tiab] OR charcoal[tiab] OR coal[tiab] OR biofuel*[tiab] OR dung[tiab] OR biomass[tiab] OR "biomass fuel"[tiab] OR "fossil fuel"[tiab] OR "solid fuel"[tiab] OR "wood smoke"[tiab] OR "crop waste"[tiab] OR "agricultural waste"[tiab] OR "crop residue"[tiab] OR "agricultural residue"[tiab] OR "plant residue"[tiab]  **AND**  "Family Characteristics"[Mesh] OR “household characteristics” OR "Health Surveys"[Mesh] OR "Cross-Sectional Studies"[Mesh]  **AND**  "Air Pollution, Indoor"[Mesh] OR "household air pollution" OR indoor*[tiab] | | | | |
| **Scopus** | cooking OR “indoor cooking” OR “outdoor cooking” OR "Cooking fuel" OR "heating fuel" OR kitchen OR hous* OR fuel OR smok* wood OR kerosene OR charcoal OR coal OR biofuel* OR dung OR biomass OR “biomass fuel” OR “fossil fuel” OR “solid fuel” OR “wood smoke” OR "crop waste" OR "agricultural waste" OR "crop residue" OR "agricultural residue" OR “plant residue”  **AND**  “family characteristics” OR “household characteristics” OR “health survey*” OR “cross-sectional stud*”  **AND**  "household air pollution" OR "indoor air pollution" | | | | |
| **Web of Science** | TS=cooking OR TS="indoor cooking" OR TS="outdoor cooking" OR TS="cooking fuel" OR TS="heating fuel" OR TS=kitchen OR TS=house OR TS= fuel OR TS=smok* OR (TS=wood OR TS=kerosene OR TS=charcoal OR TS=coal OR TS=biofuel* OR TS=dung OR TS=biomass OR TS=“biomass fuel” OR TS=“fossil fuel” OR TS=“solid fuel” OR TS=“wood smoke” OR TS="crop waste" OR TS="agricultural waste" OR TS="crop residue" OR TS="agricultural residue" OR TS=“plant residue”  **AND**  TS=“family characteristics” OR TS=“household characteristics” OR TS=“health survey*” OR TS=“cross-sectional stud*”  **AND**  TS="household air pollution" OR TS="indoor air pollution" OR TS="air pollution" | | | | |
| **The DHS program publication search portal** | | **Title or Abstract** | | cooking fuel **OR** indoor air pollution **OR** household air pollution | |
|  |  | Topics | | all | |
|  |  | Country | | any | |
|  |  | Published from | | any | |
|  |  | Journal | | any | |
