# Supplementary material for: A Systematic Review and Appraisal of Epidemiological Studies on Household Fuel Use and Its Health Effects Using Demographic and Health Surveys
Source: Int J Environ Res Public Health. 2021 Feb 3;18(4):1411. doi: 10.3390/ijerph18041411 (PMC7913474; doi:10.3390/ijerph18041411)
Supplement: Supplementary file 1 [file ijerph-18-01411-s001.zip › Supplementary files/Table S2_Quality and Risk of Bias assessment.docx]

**S2 Table. Risk of bias assessment table**

Based on **Morgan et al. (2019):** A risk of bias instrument for non-randomized studies of exposures tool [1]

1. **RoB assessment tool: Acute Respiratory Infection in Childhood**

**Step I:** review question, potential confounders, co-interventions, and exposure and outcome measurement accuracy information

| **Step I items** | **Response** |
| --- | --- |
| PECO question | Is there a difference in respiratory health risks in under-five children from households practicing indoor combustion of polluting fuel (wood, charcoal, coal, animal dung, plant residue, crop waste and kerosene) vs. the counterparts from households using clean fuel (Liquefied Petroleum Gas, electricity, biogas and solar energy?) |
| Confounding for HAP and child ARI | - Outdoor air pollutants (it can be point or/and mobile source), e.g., articulate mater, NOx, CO, … etc. - Inadequacy or unvaccinated - Nutritional status (under nourished) - Breastfeeding status (inadequate or non-breast feeding children - Exisitng health condition (asthma, HIV and TB) - smoking (presence of smoker (s) in the house; passive smoking) - crowding (number of occupants per house/room) - Age of the child - Birth weight (being small at birth) |
| Co-interventions | - None identified |
| Accuracy of the measurement of exposure to  HAP | The following techniques are placed in descending order of exposure assessment accuracy.   - Biological monitoring (collecting a sample of biomarkers from blood or urine samples) - Personal monitoring (placing a pollution monitor on an individual for a specified period) - Micro-environmental area-based monitoring (placement of the monitor near household pollution source - Questionnaire/self-report (fuel/stove type, ventilation, cooking place and behaviour, etc.). However, these indicators are more stable over a year than a single measurement of personal exposure or area of concentration. |
| Accuracy of the measurement of outcome (ARI) | The following are assessment methods for Acute Respiratory Tract Infection:   - Recall (by caregiver) of key symptoms and signs within a specified time period (recall up to two weeks). Such self-reported episodes of ARI is less accurate. - Combination of recall by caregivers of key symptoms with direct observation of signs by trained personnel under WHO guideline - Assessment by physician, chest ex-ray or blood culture - Assessment methods conducted by physician or trained health care workers base on predefined assessment criteria or diagnostic investigations are more accurate. |

**Dinabandhu Mondal, Pintu Paul (2020):** Effects of indoor pollution on acute respiratory infections among under-five children in India: Evidence from a nationally representative population-based study

**Step II:** hypothetical target experiment, including specific confounders and co-interventions from the study that will require consideration

1. target randomized trial specific to the study

| Design | Individual randomized trial |
| --- | --- |
| Participants | Nationally representative Indian children of 5 years or less old that were included in 2015-06 India NFHS). Their age is reported based on birth certificate records available with relevant civil authorities of India. |
| Experimental intervention | polluting fuels (kerosene, coal/lignite, charcoal, wood, straw/shrubs/grass, agricultural crop and animal dung) for cooking |
| Comparator | clean fuels (electricity, liquid petroleum gas (LPG), solar energy, natural gas and biogas) for cooking |

B1. Specify the outcome

Acute Respiratory Infection (ARI) in children

B2. If multiple outcomes presented, specify the numerical result being assessed

| **Confounding domains listed in step I** | | | |
| --- | --- | --- | --- |
| Confounding domain | Measured variable (s) | Is there evidence that  controlling for this  Variable was unnecessary? | Is the confounding domain measured validly and reliably by this Variable (or these variables)? |
| Ambient air pollutants, Vaccination status, child nutritional status, breast feeding status, existing health condition (infected with HIV and TB), smoking (active and/or passive), crowding, age of the child and low birth weight | None | No | No information |
| **Additional confounding domains relevant to the setting of this particular study, or which the study authors identified as important** | | | |
| sex of child, birth order, maternal: age education, ethnicity/caste, religion and TB contact, kitchen location, wealth | None | No | Yes |

**Step III: RoB across seven items**

| **Rec. No. EndNote_3524** | | | |
| --- | --- | --- | --- |
| **Bias items** | **Risk of bias** | **Direction of bias** | **Rationale** |
| Bias due to confounder | Moderate |  | Authors controlled age of child and sex of child, birth order, maternal age and education, ethnicity, religion, TB contact, persons per room, kitchen location, smoker in the house and wealth. However, some important variables indicated in step I of RoB assessment (**see step I**) not controlled. |
| Bias in selection of participants into the study | Low |  | DHS implements probability sampling technique (selection of subjects was unrelated to either exposure or outcome status)  There is no means to commit bias in selection of participants. |
| Bias in classification of exposures | Moderate |  | Authors classified exposed and non-exposed households as per standard: (1) exposed: kerosene, coal and lignite, charcoal, wood, straw/shrubs/grass, agricultural crop, animal dung, and others, and (2) non-exposed: electricity, LPG, natural gas, and biogas. However, there could be use of multiple fuels and could also impossible to count for past exposure or recent changes in cooking methods. |
| Bias due to deviations from intended exposures | Moderate |  | There is fuel staking and switch from polluting to clean fuel (but is very slow), and there could be a chance of infiltration from neighbourhood (especially in slum areas), but that might not be significant. |
| Bias due to missing data | Low |  | The analysis is based on a national survey which usually has a response rate of >90%. The probability of treating the two category of households in terms of missing information is almost nil. |
| Bias in measurement of the outcome | Serious |  | The method used to ascertain the outcome lacks robustness (**see step I**). But this bias could not be due to either differential or non-deferential error as a result of knowledge of exposure status |
| Bias in selection of the reported result | Low |  | It is unlikely to be selective in reporting of the result, because the contents in the data are pre-determined and available online in the DHS program website. Again, the descriptive result can be found in the final report of Pakistan DHS. Statistical methods reported in the methods section were used and presented in the results. |
| Overall bias | Moderate |  | The analysis is based on nationally representative data with large sample size. |

**Lubna Naz, Umesh Ghimire (2020):** Assessing the prevalence trend of childhood pneumonia associated with indoor air pollution in Pakistan

**Step II:** hypothetical target experiment, including specific confounders and co-interventions from the study that will require consideration

1. target randomized trial specific to the study

| Design | Individual randomized trial |
| --- | --- |
| Participants | Nationally representative Pakistan children of 5 years or less old that were included in three waves (2006–2018) of Pakistan DHS. Their age is reported based on birth certificate records available with relevant civil authorities of Pakistan. |
| Experimental intervention | Polluting fuels (kerosene, coal/lignite, charcoal, wood, straw/shrubs/grass, agricultural crop and animal dung) for cooking |
| Comparator | Clean fuels (electricity, liquid petroleum gas (LPG), solar energy, natural gas and biogas) for cooking |

B1. Specify the outcome

Acute Respiratory Infection (ARI) in children

B2. If multiple outcomes presented, specify the numerical result being assessed

| **Confounding domains listed in step I** | | | |
| --- | --- | --- | --- |
| Confounding domain | Measured variable (s) | Is there evidence that  controlling for this  Variable was unnecessary? | Is the confounding domain measured validly and reliably by this Variable (or these variables)? |
| Ambient air pollutants, Vaccination status, child nutritional status, breast feeding status, existing health condition (infected with HIV and TB), smoking (active and/or passive), crowding, age of the child and low birth weight | None | No | No information |
| **Additional confounding domains relevant to the setting of this particular study, or which the study authors identified as important** | | | |
| Child’s age child’s sex, birth size, mother’s age, location of kitchen, sanitation facility and place of residence | None | No | Yes |

**Step III: RoB across seven items**

| **Rec. No. EndNote_3525** | | | |
| --- | --- | --- | --- |
| **Bias items** | **Risk of bias** | **Direction of bias** | **Rationale** |
| Bias due to confounder | Moderate |  | Authors controlled Child’s age child’s sex, birth size, vaccination, mother’s age, location of kitchen, crowding, sanitation facility and place of residence. However, some important variables indicated in step I of RoB assessment (**see step I**) not controlled. |
| Bias in selection of participants into the study | Low |  | DHS implements probability sampling technique (selection of subjects was unrelated to either exposure or outcome status) |
| Bias in classification of exposures | Moderate |  | Some households could use a combination of polluting and clean fuels and DHS collects main cooking fuel item only. As is cross-sectional study, it is impossible to know past exposure or recent changes in cooking methods. But authors have no means to fix that |
| Bias due to deviations from intended exposures | Moderate |  | Even though the rate at which HHs switch from one form of fuel to another (e.g., from polluting to clean) is very slow, there could be probability. It is also difficult to control chance of infiltration from neighbourhood and from ambient air pollution. |
| Bias due to missing data | Low |  | Authors did not mention about missing data but, overall, this is national household survey with high response rate. |
| Bias in measurement of the outcome | Serious |  | The method used to ascertain the outcome is the least preferred one (less accurate) (**see step I of the RoB assessment**). Despite such a case ascertainment error, it is less likely to induce either differential or non-deferential error due to knowledge of exposure status |
| Bias in selection of the reported result | Low |  | It is unlikely to be selective in reporting of the result and the descriptive result can be found in the final report of Pakistan DHS. Also, statistical methods reported in the methods section were used and presented in the results. |
| Overall bias | Moderate |  | The analysis is based on nationally representative data with large sample size. |

**Katherine E. Woolley, Tusubira Bagambe (2020):** Investigating the Association between Wood and Charcoal Domestic Cooking, Respiratory Symptoms and Acute Respiratory Infections among Children Aged Under 5 Years in Uganda: A Cross-Sectional Analysis of the 2016 Demographic and Health Survey

**Step II:** hypothetical target experiment, including specific confounders and co-interventions from the study that will require consideration

1. target randomized trial specific to the study

| Design | Individual randomized trial |
| --- | --- |
| Participants | Nationally representative Uganda children of 5 years or less old that were included in 2016 Ugandan DHS. Their age is reported based on birth certificate records available with relevant civil authorities of Uganda. |
| Experimental intervention | Cooking with wood |
| Comparator | Cooking with charcoal |

B1. Specify the outcome

Acute Respiratory Infection (ARI) in children

B2. If multiple outcomes presented, specify the numerical result being assessed

| **Confounding domains listed in step I** | | | |
| --- | --- | --- | --- |
| Confounding domain | Measured variable (s) | Is there evidence that  controlling for this  Variable was unnecessary? | Is the confounding domain measured validly and reliably by this Variable (or these variables)? |
| Ambient air pollutants, Vaccination status, child nutritional status, breast feeding status, existing health condition (infected with HIV and TB), smoking (active and/or passive), crowding, age of the child and low birth weight | None | No | No information |
| **Additional confounding domains relevant to the setting of this particular study, or which the study authors identified as important** | | | |
| Place of cooking, season, child sex, birth order, educational status of the mother, mode of delivery, vit. A supplementation for the child and Iron supplementation for the mother, place of residence | None | No | Yes |

**Step III: RoB across seven items**

| **Rec. No. EndNote_3526** | | | |
| --- | --- | --- | --- |
| **Bias items** | **Risk of bias** | **Direction of bias** | **Rationale** |
| Bias due to confounder | Moderate |  | Authors controlled child’s age, sex, birth order, mode of delivery, vitamin A supplementation, breastfeeding, iron supplementation, maternal age, maternal education, wealth index, household smoking, cooking location, number of household remembers, season, place of residence, region. However, some important variables indicated in step I of RoB assessment (**see step I**) not controlled. |
| Bias in selection of participants into the study | Low |  | DHS implements probability sampling technique (selection of subjects was unrelated to either exposure or outcome status) |
| Bias in classification of exposures | Moderate |  | Authors compared two polluting fuels, wood and charcoal (both polluting) and some households could use a combination of these fuel types but DHS collects only one main fuel item used in the household. It is not possible to account past exposure or recent changes in cooking methods. |
| Bias due to deviations from intended exposures | Moderate |  | There is fuel staking and switch from polluting to clean fuel (but is very slow), and there could be a chance of infiltration from neighbourhood (especially in slum areas), but that might not be significant. |
| Bias due to missing data | Low |  | The total response rate of the study was high, 98 %. There could me a few missing on the variables of interest but minimal. |
| Bias in measurement of the outcome | Serious |  | The method used to ascertain the outcome is the least preferred one (less accurate) (**see step I of the RoB assessment**). Despite such a case ascertainment error, it is less likely to induce either differential or non-deferential error due to knowledge of exposure status |
| Bias in selection of the reported result | Low |  | It is unlikely to be selective in reporting of the result, because the contents in the data are pre-determined and available online in the DHS program website. Statistical methods reported in the methods section were used and presented in the results. |
| Overall bias | Moderate |  | Advanced method could be expensive in developing country so that these methods are currently used as best available option. |

**Shyam Sundar Budhathoki, Bhim Singh Tinkari (2020):** The Association of Childhood Pneumonia with Household Air Pollution in Nepal: Evidence from Nepal Demographic Health Surveys

**Step II:** hypothetical target experiment, including specific confounders and co-interventions from the study that will require consideration

1. target randomized trial specific to the study

| Design | Individual randomized trial |
| --- | --- |
| Participants | Nationally representative Nepali children of 5 years or less old that were included in three rounds (2006, 2011 and 2017) of Nepal DHS. Their age is reported based on birth certificate records available with relevant civil authorities of Nepal. |
| Experimental intervention | polluting fuels (kerosene, coal/lignite, charcoal, wood, straw/shrubs/grass, agricultural crop and animal dung) for cooking |
| Comparator | clean fuels (electricity, liquid petroleum gas (LPG), solar energy, natural gas and biogas) for cooking |

B1. Specify the outcome

Acute Respiratory Infection (ARI) in children

B2. If multiple outcomes presented, specify the numerical result being assessed

| **Confounding domains listed in step I** | | | |
| --- | --- | --- | --- |
| Confounding domain | Measured variable (s) | Is there evidence that  controlling for this  Variable was unnecessary? | Is the confounding domain measured validly and reliably by this Variable (or these variables)? |
| Ambient air pollutants, Vaccination status, child nutritional status, breast feeding status, existing health condition (infected with HIV and TB), smoking (active and/or passive), crowding, age of the child and low birth weight | None | No | No information |
| **Additional confounding domains relevant to the setting of this particular study, or which the study authors identified as important** | | | |
| Mother’s age, education and employment status, ecological region, sanitation facility, and presence of separate kitchen | None | No | Yes |

**Step III: RoB across seven items**

| **Rec. No. EndNote_3527** | | | |
| --- | --- | --- | --- |
| **Bias items** | **Risk of bias** | **Direction of bias** | **Rationale** |
| Bias due to confounder | Moderate |  | Authors controlled mother’s age, education and employment status, ecological region, sanitation facility, presence of smoker in the house, birth size, wasting status of the child and presence of separate kitchen. In addition, they considered some other covariates in their bivariate analysis level. However, some important variables indicated in step I of RoB assessment (**see step I**) not controlled. |
| Bias in selection of participants into the study | Low |  | DHS implements probability sampling technique (selection of subjects was unrelated to either exposure or outcome status) |
| Bias in classification of exposures | Moderate |  | Authors classified exposed and non-exposed group as per standard. However, some households could use a combination of polluting and clean fuels and information was only collected on primary fuel use in DHS survey. It is also impossible to count for past exposure or recent changes in cooking methods. |
| Bias due to deviations from intended exposures | Moderate |  | Even though there is high chance of exposure status change among participants, information was measured cross-sectional and analysed from single measurement/interview. Additionally, the rate at which HHs switch from polluting to clean fuel is very slow. |
| Bias due to missing data | Low |  | In all rounds, the total response rates were > 90%, and the probability of treating the two category of households differently in terms of missing information is almost nil. |
| Bias in measurement of the outcome | Serious |  | The method used to ascertain the outcome lacks robustness (**see step I**). But this bias could not be due to either differential or non-deferential error as a result of knowledge of exposure status |
| Bias in selection of the reported result | Low |  | This is secondary data analysis so that the descriptive results can be found in the national reports. In addition, statistical methods reported in the methods section were used and presented in the results. |
| Overall bias | Moderate |  | The analysis is based on nationally representative data with large sample size. |

**Juwel Rana , Jalal Uddin (2019):** Associations between Indoor Air Pollution and Acute Respiratory Infections among Under-Five Children in Afghanistan: Do SES and Sex Matter?

**Step II:** hypothetical target experiment, including specific confounders and co-interventions from the study that will require consideration

1. target randomized trial specific to the study

| Design | Individual randomized trial |
| --- | --- |
| Participants | Nationally representative Afghanistan children of 5 years or less old that were included in 2015 Afghanistan DHS. Their age is reported based on birth certificate records available with relevant civil authorities of Afghanistan. |
| Experimental intervention | polluting fuels (kerosene, coal/lignite, charcoal, wood, straw/shrubs/grass, agricultural crop and animal dung) for cooking |
| Comparator | clean fuels (electricity, liquid petroleum gas (LPG), solar energy, natural gas and biogas) for cooking |

B1. Specify the outcome

Acute Respiratory Infection (ARI) in children

B2. If multiple outcomes presented, specify the numerical result being assessed

| **Confounding domains listed in step I** | | | |
| --- | --- | --- | --- |
| Confounding domain | Measured variable (s) | Is there evidence that  controlling for this  Variable was unnecessary? | Is the confounding domain measured validly and reliably by this Variable (or these variables)? |
| Ambient air pollutants, Vaccination status, child nutritional status, breast feeding status, existing health condition (infected with HIV and TB), smoking (active and/or passive), crowding, age of the child and low birth weight | None | No | No information |
| **Additional confounding domains relevant to the setting of this particular study, or which the study authors identified as important** | | | |
| child age, child sex, maternal age at birth, maternal education, parental occupation, household wealth quintile, season, and region | None | No | Yes |

**Step III: RoB across seven items**

| **Rec. No. EndNote_3528** | | | |
| --- | --- | --- | --- |
| **Bias items** | **Risk of bias** | **Direction of bias** | **Rationale** |
| Bias due to confounder | Moderate |  | Authors controlled child age, child sex, maternal age at birth, maternal education, parental occupation, household wealth, season, and mother’s smoking status, breastfeeding status, and region. However, some risk factors/confounders (**see step I**) left uncontrolled. |
| Bias in selection of participants into the study | Low |  | DHS implements probability sampling technique (selection of subjects was unrelated to either exposure or outcome status. There is no means to commit bias in selection of participants. |
| Bias in classification of exposures | Moderate |  | Authors categorized exposed and non-exposed group as: solid fuel (e.g., kerosene, coal, lignite, charcoal, wood, animal dung, straw/shrubs/grass), and non-solid fuel (e.g., electricity, liquid petroleum gas, natural gas, biogas), respectively, which is in line with the standard. However, there could be use of multiple fuels and could also impossible to count for past exposure or recent changes in cooking methods. |
| Bias due to deviations from intended exposures | Moderate |  | Even though there is high chance of exposure status change among participants, information was measured cross-sectional and analysed from single measurement/interview. Additionally, the rate at which HHs switch from polluting to clean fuel is very slow. |
| Bias due to missing data | Low |  | The total response rate of the study was high, 98%. The probability of treating the two category of households in terms of missing information is almost nil. |
| Bias in measurement of the outcome | Serious |  | The method used to ascertain the outcome lacks robustness (**see step I**). But this bias could not be due to either differential or non-deferential error as a result of knowledge of exposure status |
| Bias in selection of the reported result | Low |  | It is unlikely to be selective in reporting of the result, because the descriptive result can be found in the final report of Pakistan DHS. Statistical methods reported in the methods section were used and presented in the results. |
| Overall bias | Moderate |  | The analysis is based on nationally representative data with large sample size. |

**Study title:** **Khan et al. (2018):** Household air pollution from cooking fuel and respiratory health risks for children in Pakistan

**Step II:** hypothetical target experiment, including specific confounders and co-interventions from the study that will require consideration

1. target randomized trial specific to the study

| Design | Individual randomized trial |
| --- | --- |
| Participants | Nationally representative Pakistan children of 5 years or less old that were included in 2012-13 Pakistan DHS. Their age is reported based on birth certificate records available with relevant civil authorities of Pakistan. |
| Experimental intervention | polluting fuels (kerosene, coal/lignite, charcoal, wood, straw/shrubs/grass, agricultural crop and animal dung) for cooking |
| Comparator | clean fuels (electricity, liquid petroleum gas (LPG), solar energy, natural gas and biogas) for cooking |

B1. Specify the outcome

Acute Respiratory Infection (ARI) in children

B2. If multiple outcomes presented, specify the numerical result being assessed

| **Confounding domains listed in step I** | | | |
| --- | --- | --- | --- |
| Confounding domain | Measured variable (s) | Is there evidence that  controlling for this  Variable was unnecessary? | Is the confounding domain measured validly and reliably by this Variable (or these variables)? |
| Ambient air pollutants, Vaccination status, child nutritional status, breast feeding status, existing health condition (infected with HIV and TB), smoking (active and/or passive), crowding, age of the child and low birth weight | None | No | No information |
| **Additional confounding domains relevant to the setting of this particular study, or which the study authors identified as important** | | | |
| separate kitchen in the house, child sex, birth order, household crowding, educational status and mother’s age at child birth | None | No | Yes |

**Step III: RoB across seven items**

| **Rec. No. EndNote_51** | | | |
| --- | --- | --- | --- |
| **Bias items** | **Risk of bias** | **Direction of bias** | **Rationale** |
| Bias due to confounder | Low |  | Authors controlled some important confounders, albeit some other important confounders not controlled. However, some important variables indicated in step I of RoB assessment (**see step I**) not controlled. |
| Bias in selection of participants into the study | Low |  | DHS implements probability sampling technique (selection of subjects was unrelated to either exposure or outcome status)  There is no means to commit bias in selection of participants. |
| Bias in classification of exposures | Moderate |  | There may be misclassification in categorizing participants into exposed to polluting fuel or not because some households could use a combination of polluting and clean fuels and information was only collected on primary fuel use in DHS survey. This study also did not account for past exposure to cooking fuel or recent changes in cooking methods. |
| Bias due to deviations from intended exposures | Moderate |  | Even though there is high chance of exposure status change among participants, information was measured cross-sectional and analyzed from single measurement/interview. Additionally, the rate at which HHs switch from polluting to clean fuel is very slow. However, there is a chance of neighborhood and ambient air pollution from a household using polluted fuel. |
| Bias due to missing data | Low |  | Even though the study authors fail to mention how they had managed missing information on some of the variables, the total response rate of the study was high, 96 %. The probability of treating the two category of households in terms of missing information is almost nil. |
| Bias in measurement of the outcome | Serious |  | The method used to ascertain the outcome lacks robustness (**see step I**). But this bias could not be due to either differential or non-deferential error as a result of knowledge of exposure status |
| Bias in selection of the reported result | Low |  | It is unlikely to be selective in reporting of the result, because the contents in the data are pre-determined and available online in the DHS program website. Again, the descriptive result can be found in the final report of Pakistan DHS. Statistical methods reported in the methods section were used and presented in the results. |
| Overall bias | Moderate |  | The analysis is based on nationally representative data with large sample size. |

**Study title:** **Capuno et al. (2018):** Cooking and coughing: Estimating the effects of clean fuel for cooking on the respiratory health of children in the Philippines

**Step II:** hypothetical target experiment, including specific confounders and co-interventions from the study that will require consideration

1. target randomized trial specific to the study

| Design | Individual randomized trial |
| --- | --- |
| Participants | Nationally representative Pilipino children aged 5 years or less old that were included in 2013 NDHS, Philippines. |
| Experimental intervention | polluting fuels (kerosene, coal/lignite, charcoal, wood, straw/shrubs/grass, agricultural crop and animal dung) for cooking |
| Comparator | clean fuels (electricity, liquid petroleum gas (LPG), solar energy, natural gas and biogas) for cooking |

B1. Specify the outcome

Acute Respiratory Infection (ARI) in children

B2. If multiple outcomes presented, specify the numerical result being assessed

| **Confounding domains listed in step I** | | | |
| --- | --- | --- | --- |
| Confounding domain | Measured variable (s) | Is there evidence that controlling for this Variable was unnecessary? | Is the confounding domain measured validly and reliably by this Variable (or these variables)? |
| Ambient air pollutants, Vaccination status, child nutritional status, breast feeding status, existing health condition (infected with HIV and TB), smoking (active and/or passive), crowding, age of the child and low birth weight | None | No | No information |
| **Additional confounding domains relevant to the setting of this particular study, or which the study authors identified as important** | | | |
| Sex of the child, Child’s birth order, Child lives with mother, Is child or grandchild, household income, having health insurance, ethnicity  family size, sex of head of the household, age of the mother, maternal education, marital status, father's age, father's education, father's employment status, place of residence, religion | None | No | Yes |

**Step III: RoB across seven items**

| **Rec. No. EndNote_98** | | | |
| --- | --- | --- | --- |
| **Bias items** | **Risk of bias** | **Direction of bias** | **Rationale** |
| Bias due to confounder | Critical |  | Most of important risk factors not controlled. Most of the variables controlled by authors could be important to specific setting but the known & identified risk factors not controlled |
| Bias in selection of participants into the study | low |  | DHS implements probability sampling technique (selection of subjects was unrelated to either exposure or outcome status)  There is no means to commit bias in selection of participants. |
| Bias in classification of exposures | Moderate |  | There may be misclassification in categorizing participants into exposed to polluting fuel or not because some households could use a combination of polluting and clean fuels and information was only collected on primary fuel use in DHS survey.  Authors also included households those who did not report any fuel type but did claim not to cook food at home in the polluted fuel category. This study also did not account for past exposure to cooking fuel or recent changes in cooking methods. |
| Bias due to deviations from intended exposures | Moderate |  | Even though there is high chance of exposure status change among participants, information was measured cross-sectional and analyzed from single measurement/interview. However, there is a chance of neighborhood and ambient air pollution from a household using polluted fuel. |
| Bias due to missing data | Low |  | The study had a total of 91.5% response rate. Authors considered in the analysis only children for whom there is complete information on health outcome status and other pertinent covariates. There is no indication that authors had treated the two group (households with polluted fuel and household with clean fuel) differently |
| Bias in measurement of the outcome | Serious |  | The method used to ascertain the outcome lacks robustness (**see step I**). |
| Bias in selection of the reported result | Low |  | The data are pre-determined and available online in the DHS program website in Pakistan DHS. Again, methods reported in the methods section were used and presented in the results. |
| Overall bias | Serious |  | Many important confounders of ARI were not controlled, but the study has large sample with nationally representative data |

**Study title:** **Akinyemi et al. (2018):** Household environment and symptoms of childhood acute respiratory tract infections in Nigeria, 2003–2013: a decade of progress and stagnation

**Step II:** hypothetical target experiment, including specific confounders and co-interventions from the study that will require consideration

1. target randomized trial specific to the study

| Design | Individual randomized trial |
| --- | --- |
| Participants | Nationally representative Nigerian children aged 5 years or less old that were included in 2003 to 2013 NDHS. |
| Experimental intervention | polluting fuels (kerosene, coal/lignite, charcoal, wood, straw/shrubs/grass, agricultural crop and animal dung) for cooking |
| Comparator | clean fuels (electricity, liquid petroleum gas (LPG), solar energy, natural gas and biogas) for cooking |

B1. Specify the outcome

Acute Respiratory Infection (ARI) in children

B2. If multiple outcomes presented, specify the numerical result being assessed

| **Confounding domains listed in step I** | | | |
| --- | --- | --- | --- |
| Confounding domain | Measured variable (s) | Is there evidence that  controlling for this Variable was unnecessary? | Is the confounding domain measured validly and reliably by this Variable (or these variables)? |
| Ambient air pollutants, Vaccination status, child nutritional status, breast feeding status, existing health condition (infected with HIV and TB), smoking (active and/or passive), crowding, low birth weight, and age of the child | Nutritional status | No | No information |
| **Additional confounding domains relevant to the setting of this particular study, or which the study authors identified as important** | | | |
| environment/household related: Smoking status by household members, source of drinking water, status of toilet, quality of housing material, wealth quintile, residence, region, season of interview, Mother related: age, education & occupation, Child related: sex, birth order and nutritional status | None | No | Yes |

**Step III: RoB across seven items**

| **Rec. No. EndNote_98** | | | |
| --- | --- | --- | --- |
| **Bias items** | **Risk of bias** | **Direction of bias** | **Rationale** |
| Bias due to confounder | Moderate |  | Authors declared that they were unable controlling vaccination status of the child because data on vaccination was available only for children less than 36 months. Though they mentioned this, other factors such as breast feeding status, birth weight and existing health status were not considered. Nutritional status is also considered chronically; only stunting. |
| Bias in selection of participants into the study | low |  | DHS implements probability sampling technique (selection of subjects was unrelated to either exposure or outcome status)  There is no means to commit bias in selection of participants. |
| Bias in classification of exposures | Moderate |  | Authors categorized fuel as clean (electricity, liquefied petroleum gas, natural gas or biogas) and unclean (coal, ignite, charcoal, wood, kerosene, animal dung, straw, shrubs, and grass). Even though the category had no problem, there could be misclassification in categorizing participants into exposed to unclean fuel or not because some households could use a combination of unclean and clean fuels and information was only collected on primary fuel use in DHS survey. |
| Bias due to deviations from intended exposures | Moderate |  | Even though there is high chance of exposure status change among participants, information was measured cross-sectional and analyzed from single measurement/interview. However, there is a chance of neighborhood and ambient air pollution from a household using polluted fuel. |
| Bias due to missing data | Low |  | Even though authors did not mentioned issues related to missing data, the result of the three waves report showed all more than 90% response rate. |
| Bias in measurement of the outcome | Serious |  | The method used to ascertain the outcome lacks robustness (**see step I**). But this bias could not be due to either differential or non-deferential error as a result of knowledge of exposure status |
| Bias in selection of the reported result | Low |  | It is less likely to report/present the report selectively, because the finding can easily be compared with NDHS final report. |
| Overall bias | Moderate |  |  |

**Title of the study et al Khan 2017:** Household air pollution from cooking and risk of adverse health and birth outcomes in Bangladesh: a nationwide population based study

**Step II:** hypothetical target experiment, including specific confounders and co-interventions from that study that will require consideration

1. target randomized trial specific to the study

| Design | Household randomized trial |
| --- | --- |
| Participants | Nationally representative Bangladesh under-five children included in BDHS collected in 2007, 2011 and 2014, live-born children within five years preceding the survey; only outcomes of the most recent births were considered |
| Experimental intervention | polluting fuels (kerosene, coal/lignite, charcoal, wood, straw/shrubs/grass, agricultural crop and animal dung) for cooking |
| Comparator | clean fuels (electricity, liquid petroleum gas (LPG), natural gas and biogas) for cooking |

B1. Specify the outcome

ARI

B2. If multiple outcomes presented, specify the numerical result being assessed

| **Confounding domains listed in step I** | | | |
| --- | --- | --- | --- |
| Confounding domain | Measured variable (s) | Is there evidence that controlling for this Variable was unnecessary? | Is the confounding domain measured validly and reliably by this Variable (or these variables)? |
| Ambient air pollutants, Vaccination status, child nutritional status, breast feeding status, existing health condition (infected with HIV and TB), smoking (active and/or passive), crowding, low birth weight, and age of the child | None | No | No information |
| **Additional confounding domains relevant to the setting of this particular study, or which the study authors identified as important** | | | |
| educational attainment, region of residence, place of residence and children’s gender | None | No | Yes |

Step III: RoB across seven items

| **Rec. No. EndNote_280** | | | |
| --- | --- | --- | --- |
| **Bias items** | **Risk of**  **bias** | **Direction of bias** | **Rationale** |
| Bias due to confounding | Critical |  | Authors controlled women’s age at birth, wealth quintiles, educational attainment, region of residence, place of residence and children’s gender. Other variables like children’s nutritional status, water and hygiene status were not considered as confounders. |
| Bias in selection of participants  into the study | low |  | DHS implements probability sampling technique (selection of subjects was unrelated to either exposure or outcome status)  There is no means to commit bias in selection of participants. |
| Bias in classification of  exposures | Serious |  | Authors classified cooking fuel in to two groups: solid and clean fuel. Even though kerosene is one of the fuel options in the category, authors have not mentioned what happened to kerosene; excluded or not.  In addition to this, some households could use a combination of solid and clean fuels, but this is not addressed in the survey. Furthermore, the study also did not account for past exposure to cooking fuel or recent changes in cooking methods. |
| Bias due to deviations from intended exposures | Moderate |  | The DHS is cross-sectional; there could be fuel use/choice pattern shift among households at some point in the past time. When households economically become stronger, they tend to shift to cleaner fuel. |
| Bias due to missing data | Low |  | The analysis is based on nationally representative sample, which 98% combined response rate. Yet again, missed information could be less likely to be specific to one category. |
| Bias in measurement of the outcome | Serious |  | The method used to ascertain the outcome lacks robustness (**see step I**). But this bias could not be due to either differential or non-deferential error as a result of knowledge of exposure status |
| Bias in selection of the  reported result | Low |  | All the variables considered in the analysis found in the dataset and the descriptive result can be found in the final report, so that it is less likely to commit bias in selection of the reported result. |
| Overall bias | Critical |  | Many risk factors of ARI missed to be controlled and one of the polluting fuels, kerosene not mentioned. |

**Study title**: **Daniel (2016):** Effects of Biomass Fuel on Child Acute Respiratory Infections in Rural Areas of Cameroon and Gabon

**Step II:** hypothetical target experiment, including specific confounders and co-interventions from the study that will require consideration

1. target randomized trial specific to the study

| Design | Individual randomized trial |
| --- | --- |
| Participants | Nationally representative rural Cameroon and Gabon children aged 5 years or less old that were included in 2011 CDHS and 2012 GDHS. |
| Experimental intervention | polluting fuels (kerosene, coal/lignite, charcoal, wood, straw/shrubs/grass, agricultural crop and animal dung) for cooking |
| Comparator | clean fuels (electricity, liquid petroleum gas (LPG), solar energy, natural gas and biogas) for cooking |

B1. Specify the outcome

Acute Respiratory Infection (ARI) in children

B2. If multiple outcomes presented, specify the numerical result being assessed

| **Confounding domains listed in step I** | | | |
| --- | --- | --- | --- |
| Confounding domain | Measured variable (s) | Is there evidence that  controlling for this Variable was unnecessary? | Is the confounding domain measured validly and reliably by this Variable (or these variables)? |
| Ambient air pollutants, Vaccination status, child nutritional status, breast feeding status, existing health condition (infected with HIV and TB), smoking (active and/or passive), crowding, low birth weight, and age of the child | Nutritional status | No | No information |
| **Additional confounding domains relevant to the setting of this particular study, or which the study authors identified as important** | | | |
| floor material, cooking place, region, mother's age at child birth, mother's level of education, birth order, use of health care service, wealth index and child's | None | No | Yes |

**Step III: RoB across seven items**

| **Rec. No. EndNote_2738** | | | |
| --- | --- | --- | --- |
| **Bias items** | **Risk of bias** | **Direction of bias** | **Rationale** |
| Bias due to confounder | Serious |  | Author controlled the following confounders: floor material, cooking place, region, child's age in month, mother's age at childbirth, mother's level of education, overcrowding, birth order, use of health care service, wealth index and child's nutritional status (stunted Vs not stunted). However, important variables indicated under step one were not controlled. In addition to this, nutritional status is also considered chronically; only stunting. |
| Bias in selection of participants into the study | low |  | DHS implements probability sampling technique (selection of subjects was unrelated to either exposure or outcome status)  There is no means to commit bias in selection of participants. |
| Bias in classification of exposures | Critical |  | Some households could use a combination of polluting and clean fuels and DHS collects main cooking fuel item only. As is cross-sectional study, it is impossible to know past exposure or recent changes in cooking methods. But authors have no means to fix that.  “Data show that very few households in rural areas used only cleaner energy. Rather, a mix of fuels were used by safer households, mainly gas and biomass fuel. Therefore, three main categories were finally obtained: “gas/electricity and/or biomass fuel”, “biomass fuel without gas/electricity” and “other fuels”. A lower likelihood of ARI among houses using gas and/or biomass fuel compared to those using only biomass fuel would show evidence of the good effect of using cleaner energy. |
| Bias due to deviations from intended exposures | Moderate |  | Even though there is high chance of exposure status change among participants, information was measured cross-sectional and analyzed from single measurement/interview. However, there is a chance of neighborhood and ambient air pollution from a household using polluted fuel. |
| Bias due to missing data | Low |  | Even though author did not mentioned issues related to missing data, the result of the report showed 99.0% for Cameroon and 97.6% for Gabon response rate. |
| Bias in measurement of the outcome | Serious |  | The method used to ascertain the outcome lacks robustness (**see step I**). But this bias could not be due to either differential or non-deferential error as a result of knowledge of exposure status |
| Bias in selection of the reported result | Low |  | It is no way to report/present the report selectively. Statistics described under the method also reported in the result. Full data can be found in the two countries final DHS report. |
| Overall bias | Critical |  |  |

**Study title:** **Wichmann et al. (2015):** Impact of cooking and heating fuel use on acute respiratory health of preschool children in South Africa

**Step II:** hypothetical target experiment, including specific confounders and co-interventions from the study that will require consideration

1. target randomized trial specific to the study

| Design | Individual randomized trial |
| --- | --- |
| Participants | Nationally representative South Africa children aged 5 years or less old that were included in 1998 SADHS, only singleton birth children |
| Experimental intervention | polluting fuels (kerosene, coal/lignite, charcoal, wood, straw/shrubs/grass, agricultural crop and animal dung) for cooking |
| Comparator | clean fuels (electricity, liquid petroleum gas (LPG), solar energy, natural gas and biogas) for cooking |

B1. Specify the outcome

Acute Respiratory Infection (ARI) in children

B2. If multiple outcomes presented, specify the numerical result being assessed

| **Confounding domains listed in step I** | | | |
| --- | --- | --- | --- |
| Confounding domain | Measured variable (s) | Is there evidence that controlling for this Variable was unnecessary? | Is the confounding domain measured validly and reliably by this Variable (or these variables)? |
| Ambient air pollutants, Vaccination status, child nutritional status, breast feeding status, existing health condition (infected with HIV and TB), smoking (active and/or passive), crowding, low birth weight, and age of the child | None | No | No information |
| **Additional confounding domains relevant to the setting of this particular study, or which the study authors identified as important** | | | |
| Child related: Age, sex and birth order, mother related: age, educational status, ethnicity  Household related: family size | None | No | Yes |

**Step III: RoB across seven items**

| **Rec. No. EndNote_2742** | | | |
| --- | --- | --- | --- |
| **Bias items** | **Risk of bias** | **Direction of bias** | **Rationale** |
| Bias due to confounder | Serious |  | Authors controlled the following confounders: Child related: Age, sex and birth order, mother related: age, educational status, ethnicity, Household related: family size. There were many important confounders missed to be controlled. |
| Bias in selection of participants into the study | low |  | DHS implements probability sampling technique (selection of subjects was unrelated to either exposure or outcome status)  There is no means to commit bias in selection of participants. |
| Bias in classification of exposures | Serious |  | There may be misclassification in categorizing participants into exposure to polluting fuel or not because some households could use a combination of polluting and clean fuels and information was only collected on primary fuel use in DHS survey. The survey also did not account for past exposure to cooking fuel or recent changes in cooking methods.  “a small residual category of other fuels used for cooking and heating (n=6=, 0.13% of the sample excluded from analysis due to unknown nature of the fuels in the category”. |
| Bias due to deviations from intended exposures | Moderate |  | Even though there is high chance of exposure status change among participants, information was measured cross-sectional and analyzed from single measurement/interview. However, there is a chance of neighborhood and ambient air pollution from a household using polluted fuel. |
| Bias due to missing data | Low |  | The survey has 97% response rate for HHs questionnaire and 92.3% for women's questionnaire. Very few data excluded because of incompleteness of information; “a small residual category of other fuels used for cooking and heating (n=6=, 0.13% of the sample excluded from analysis due to unknown nature of the fuels in the category”, but this is not specific to one group. |
| Bias in measurement of the outcome | Serious |  | it is subjective and ascertained by caregivers report and defined as “For children who had been ill during the last two weeks and breath faster than usual with short and rapid breath, at any time during the last two weeks, defined as having ALRI”. |
| Bias in selection of the reported result | Low |  | It is no way to report/present the report selectively. Statistics described under the method also reported in the result. Full data can be found in the two countries final DHS report. |
| Overall bias | Serious |  | Many important variables not controlled |

**Study title:** **Buchner et al. (2015):** Cooking and Season as Risk Factors for Acute Lower Respiratory Infections in African Children: A Cross-Sectional Multi-Country Analysis

**Step II:** hypothetical target experiment, including specific confounders and co-interventions from the study that will require consideration

1. target randomized trial specific to the study

| Design | Individual randomized trial |
| --- | --- |
| Participants | Nationally representative sub-Saharan Africa children aged 5 years or less old that were included in DHS conducted between 2000 and 2011. Child only from de jure residents were considered and only youngest child of every household included |
| Experimental intervention | polluting fuels (kerosene, coal/lignite, charcoal, wood, straw/shrubs/grass, agricultural crop and animal dung) for cooking |
| Comparator | clean fuels (electricity, liquid petroleum gas (LPG), solar energy, natural gas and biogas) for cooking |

B1. Specify the outcome

Acute Respiratory Infection (ARI) in children

B2. If multiple outcomes presented, specify the numerical result being assessed

| **Confounding domains listed in step I** | | | |
| --- | --- | --- | --- |
| Confounding domain | Measured variable (s) | Is there evidence that  controlling for this Variable was unnecessary? | Is the confounding domain measured  validly and reliably by this Variable (or these variables)? |
| Ambient air pollutants, Vaccination status, child nutritional status, breast feeding status, existing health condition (infected with HIV and TB), smoking (active and/or passive), crowding, low birth weight, and age of the child | Nutritional status | No | No information |
| **Additional confounding domains relevant to the setting of this particular study, or which the study authors identified as important** | | | |
| Birth order, Stove ventilation, Cooking location, Time to nearest water source, Shelter index, wealth index, maternal & paternal education, possession of health card, rainy season, geographic location, religion, sex & age of HH head | None | No | Yes |

**Step III: RoB across seven items**

| **Rec. No. EndNote_293** | | | |
| --- | --- | --- | --- |
| **Bias items** | **Risk of bias** | **Direction of bias** | **Rationale** |
| Bias due to confounder | Low |  | Authors controlled the following confounders: child age, birth order, maternal smoking, stove ventilation, cooking location, crowding, time to nearest water source, shelter index, wealth index, maternal & paternal education, possession of health card, breast feeding duration, stunting, vaccination index, rainy season, geographic location, religion, sex & age of HH head. Ambient air pollutants, birth weight and existing health condition were not controlled. Authors declared that some factors such as HIV status could not be assessed through proxies and the variable birth weight was not included due to poor quality, with mother’s recall of a child’s size at birth. |
| Bias in selection of participants into the study | low |  | DHS implements probability sampling technique (selection of subjects was unrelated to either exposure or outcome status)  There is no means to commit bias in selection of participants. |
| Bias in classification of exposures | Moderate |  | Authors categorized exposure status in to four groups, which could help to see the independent effect of each category. the exposure categories were: 1. Kerosene, 2. Coal, charcoal 3. Wood and 4. Lower-grade biomass (straw, shrubs, grass, crop residues, dung). Nevertheless, the two formerly mentioned points imposed exposure classification bias. But DHS does not account for past exposure to cooking fuel or recent changes in cooking methods. |
| Bias due to deviations from intended exposures | Moderate |  | Even though there is high chance of exposure status change among participants, information was measured cross-sectional and analyzed from single measurement/interview. However, there is a chance of neighborhood and ambient air pollution from a household using polluted fuel. |
| Bias due to missing data | Low |  | The following were considered by the authors in order to handle missing data: “Due to unavailability of key explanatory variables model building for the cooking fuel analysis was undertaken in a pooled dataset of 15 countries, and the final model re-run in a pooled dataset of18 countries. As detailed information on cooking practices was only available for selected countries, the cooking location (9 countries) and stove ventilation (6 countries) analyses were undertaken in distinct datasets”. The points mentioned above are not unique for specific group, so that bias due to missing data couldn’t significantly affect the study. |
| Bias in measurement of the outcome | Serious |  | It is subjective and ascertained by caregivers report and defined as “children with ALRI as those who had ‘cough‘ and ‘short rapid breath‘ or ‘problems in the chest or a blocked or running nose‘ in the two weeks preceding the survey”. |
| Bias in selection of the reported result | Low |  | It is no way to report/present the report selectively. Statistics described under the method also reported in the result. Full data can be found in the two countries final DHS report. |
| Overall bias | Moderate |  |  |

**Study title:** **Acharya et al. (2015):** Solid Fuel in Kitchen and Acute Respiratory Tract Infection Among Under Five Children: Evidence from Nepal Demographic and Health Survey 2011

**Step II:** hypothetical target experiment, including specific confounders and co-interventions from the study that will require consideration

1. target randomized trial specific to the study

| Design | Individual randomized trial |
| --- | --- |
| Participants | Nationally representative Nepali children aged 5 years or less old that were included in 2011 NDHS, children whose usual place of residence is Nepal only |
| Experimental intervention | polluting fuels (kerosene, coal/lignite, charcoal, wood, straw/shrubs/grass, agricultural crop and animal dung) for cooking |
| Comparator | clean fuels (electricity, liquid petroleum gas (LPG), solar energy, natural gas and biogas) for cooking |

B1. Specify the outcome

Acute Respiratory Infection (ARI) in children

B2. If multiple outcomes presented, specify the numerical result being assessed

| **Confounding domains listed in step I** | | | |
| --- | --- | --- | --- |
| Confounding domain | Measured variable (s) | Is there evidence that controlling for this Variable was unnecessary? | Is the confounding domain measured validly and reliably by this Variable (or these variables)? |
| Ambient air pollutants, Vaccination status, child nutritional status, breast feeding status, existing health condition (infected with HIV and TB), smoking (active and/or passive), crowding, low birth weight, and age of the child | None | No | No information |
| **Additional confounding domains relevant to the setting of this particular study, or which the study authors identified as important** | | | |
| Child related: sex, and birth order, geographic variables: urban/rural, ecological zone and development region and socio-economic variables: family economic status and mother’s education | None | No | Yes |

**Step III: RoB across seven items**

| **Rec. No. EndNote_298** | | | |
| --- | --- | --- | --- |
| **Bias items** | **Risk of bias** | **Direction of bias** | **Rationale** |
| Bias due to confounder | Serious |  | Authors controlled the following confounders: Child related: age, sex, and birth order, geographic variables: urban/rural, ecological zone and development region and socio-economic variables: family economic status mother’s education, family size and smoking status of mother. |
| Bias in selection of participants into the study | low |  | DHS implements probability sampling technique (selection of subjects was unrelated to either exposure or outcome status)  There is no means to commit bias in selection of participants. |
| Bias in classification of exposures | Serious |  | Authors categorized the primary cooking fuels used by household into solid fuel and cleaner fuel. Solid fuel includes wood, animal dung, straw, shrubs, grass, crop residue, coal, lignite and charcoal. Cleaner fuel comprises of liquid petroleum gas, biogas, electricity, natural gas and kerosene. They included kerosene in the clean fuel category, which could impose serious risk of bias in classification of exposure. |
| Bias due to deviations from intended exposures | Moderate |  | Even though there is high chance of exposure status change among participants, information was measured cross-sectional and analyzed from single measurement/interview. However, there is a chance of neighborhood and ambient air pollution from a household using polluted fuel and people might have used combination of solid fuels and cleaner fuel for cooking. |
| Bias due to missing data | Low |  | The study had 97.6% response rate for women questionnaire in which child related information were collected. |
| Bias in measurement of the outcome | Serious |  | It is subjective and ascertained by caregivers report and defined as “Children, who had cough accompanied by short/rapid breath and problem in chest were defined as symptoms of ARI and was categorized as a binary outcome variable”. |
| Bias in selection of the reported result | Low |  | It is no way to report/present the report selectively. Statistics described under the method also reported in the result. Full data can be found in the two countries final DHS report. |
| Overall bias | Serious |  | Authors missed many important factors in their analysis |

**Study title:** **Patel et al.** (2013): Childhood illness in households using biomass fuels in India: secondary data analysis of nationally representative national family health surveys

**Step II:** hypothetical target experiment, including specific confounders and co-interventions from the study that will require consideration

1. target randomized trial specific to the study

| Design | Individual randomized trial |
| --- | --- |
| Participants | Nationally representative Indian children aged 0-36 months old that were included in in INFHS conducted between 1992 to 2006, only last two children under 36 months of age for ever-married women included |
| Experimental intervention | polluting fuels (kerosene, coal/lignite, charcoal, wood, straw/shrubs/grass, agricultural crop and animal dung) for cooking |
| Comparator | clean fuels (electricity, liquid petroleum gas (LPG), solar energy, natural gas and biogas) for cooking |

B1. Specify the outcome

Acute Respiratory Infection (ARI) in children

B2. If multiple outcomes presented, specify the numerical result being assessed

| **Confounding domains listed in step I** | | | |
| --- | --- | --- | --- |
| Confounding domain | Measured variable (s) | Is there evidence that controlling for this Variable was unnecessary? | Is the confounding domain measured validly and reliably by this Variable (or these variables)? |
| Ambient air pollutants, Vaccination status, child nutritional status, breast feeding status, existing health condition (infected with HIV and TB), smoking (active and/or passive), crowding, low birth weight, and age of the child | None | No | No information |
| **Additional confounding domains relevant to the setting of this particular study, or which the study authors identified as important** | | | |
| Maternal age, education and working status, child gender, household standard of living and aware of media, ethnicity, region and residence | None | No | Yes |

**Step III: RoB across seven items**

| **Rec. No. EndNote_449** | | | |
| --- | --- | --- | --- |
| **Bias items** | **Risk of bias** | **Direction of bias** | **Rationale** |
| Bias due to confounder | Critical |  | Authors controlled the following confounders: child age, birth weight, sex, maternal age, education and working status, household standard of living and aware of media, ethnicity, region and residence. Other important risk factors like ambient air pollutants, vaccination status, child nutritional status, breast feeding status, existing health condition (infected with HIV and TB), smoking status (active and/or passive), and crowding index were not controlled. |
| Bias in selection of participants into the study | low |  | DHS implements probability sampling technique (selection of subjects was unrelated to either exposure or outcome status)  There is no means to commit bias in selection of participants. |
| Bias in classification of exposures | Moderate |  | There may be misclassification in categorizing participants into exposed to unclean fuel or not because some households could use a combination of unclean and clean fuels and information was only collected on primary fuel use in DHS survey. The survey also did not account for past exposure to cooking fuel or recent changes in cooking methods.  In their study, authors categorized fuel use as ‘high polluting fuel’ (HPF) for households using predominantly wood, agricultural waste, dung, or straw, ‘medium polluting fuel’ (MPF) for households using coal/lignite, charcoal, or kerosene, and low polluting fuel (LPF) for LPG/ natural gas or electricity. |
| Bias due to deviations from intended exposures | Moderate |  | Even though there is high chance of exposure status change among participants, information was measured cross-sectional and analyzed from single measurement/interview. However, there is a chance of neighborhood and ambient air pollution from a household using polluted fuel and people might have used combination of solid fuels and cleaner fuel for cooking. |
| Bias due to missing data | Low |  | It is nationally representative data with very few missing (all >90% response rate) information which were related to neither exposure nor outcome status of the subject. |
| Bias in measurement of the outcome | Serious |  | It is subjective and ascertained by caregivers report and defined as “ALRI if child had a cough accompanied by rapid breathing”. |
| Bias in selection of the reported result | Low |  | It is no way to report/present the report selectively. Statistics described under the method also reported in the result. Full data can be found in the two countries final NFHS report. |
| Overall bias | Critical |  | Confounding variables not well addressed |

**Study title**: **Kilabuko et al. (2007):** Effects of Cooking Fuels on Acute Respiratory Infections in Children in Tanzania

**Step II:** hypothetical target experiment, including specific confounders and co-interventions from the study that will require consideration

1. target randomized trial specific to the study

| Design | Individual randomized trial |
| --- | --- |
| Participants | Nationally representative Tanzanian children aged 5 years old that were included in in 2004-5 TDHS. |
| Experimental intervention | polluting fuels (kerosene, coal/lignite, charcoal, wood, straw/shrubs/grass, agricultural crop and animal dung) for cooking |
| Comparator | clean fuels (electricity, liquid petroleum gas (LPG), solar energy, natural gas and biogas) for cooking |

B1. Specify the outcome

Acute Respiratory Infection (ARI) in children

B2. If multiple outcomes presented, specify the numerical result being assessed

| **Confounding domains listed in step I** | | | |
| --- | --- | --- | --- |
| Confounding domain | Measured variable (s) | Is there evidence that  controlling for this  Variable was unnecessary? | Is the confounding domain measured validly and reliably by this Variable (or these variables)? |
| Ambient air pollutants, Vaccination status, child nutritional status, breast feeding status, existing health condition (infected with HIV and TB), smoking (active and/or passive), crowding, low birth weight, and age of the child | None | No | No information |
| **Additional confounding domains relevant to the setting of this particular study, or which the study authors identified as important** | | | |
| sex of the child, mother's educational status and her age at birth of the baby, region, residence (urban/rural) and living standard | None | No | Yes |

**Step III: RoB across seven items**

| **Rec. No. EndNote_2741** | | | |
| --- | --- | --- | --- |
| **Bias items** | **Risk of bias** | **Direction of bias** | **Rationale** |
| Bias due to confounder | Critical |  | Authors controlled the following confounders: sex and age of the child, mother's educational status and her age at birth of the baby, region and residence (urban/rural) and living standard. However, many variables mentioned under step I were missed. Authors also acknowledge this: “this study may have failed to incorporate into analysis all potential confounders. Some of these factors may include nutritional status of the child, birth order of the child, ventilation of the house or kitchen, child care practice, prenatal health, birth weight of the child, number of people living with the child in the same house (crowding) and environmental tobacco smoke”. |
| Bias in selection of participants into the study | low |  | DHS implements probability sampling technique (selection of subjects was unrelated to either exposure or outcome status)  There is no means to commit bias in selection of participants. |
| Bias in classification of exposures | Critical |  | There may be misclassification in categorizing participants into exposed to unclean fuel or not because some households could use a combination of unclean and clean fuels and information was only collected on primary fuel use in DHS survey. The survey also did not account for past exposure to cooking fuel or recent changes in cooking methods.  Authors categorized children in their analysis into two groups: group from homes using biomass fuels (firewood, straw, dung and crop residuals) and group using charcoal or kerosene. They excluded children from homes cooking on fuels other than biomass fuels, charcoal and kerosene. The source of bias here is author’s consideration of kerosene and charcoal as less polluted fuel. |
| Bias due to deviations from intended exposures | Critical |  | Even though there is high chance of exposure status change among participants, information was measured cross-sectional and analyzed from single measurement/interview. However, there is a chance of neighborhood and ambient air pollution from a household using polluted fuel and people might have used combination of solid fuels and cleaner fuel for cooking. Excluding clean fuel category and taking another polluting fuel types as a comparator is an indication of deviation from intended exposure. |
| Bias due to missing data | Low |  | It is nationally representative data with very few missing information which were related to neither exposure nor outcome status of the subject. |
| Bias in measurement of the outcome | Serious |  | It is subjective and ascertained by caregivers report and defined as “For children who had cough, the mother was additionally asked if the child, when sick with cough, breathed faster than usual with short, rapid breaths, any time during the last two weeks prior to the survey”. |
| Bias in selection of the reported result | Low |  | It is no way to report/present the report selectively. Statistics described under the method also reported in the result. Full data can be found in the two countries final DHS report. |
| Overall bias | Critical |  | Confounding factors not controlled |

**Study title:** **Mishra et al. (2005):** Effects of Cooking Smoke and Environmental Tobacco Smoke on Acute Respiratory Infections in Young Indian Children

**Step II:** hypothetical target experiment, including specific confounders and co-interventions from the study that will require consideration

1. target randomized trial specific to the study

| Design | Individual randomized trial |
| --- | --- |
| Participants | Nationally representative Indian children aged 0-35 months of age included in India’s 1998–1999 National Family Health Survey (NFHS-2). |
| Experimental intervention | polluting fuels (kerosene, coal/lignite, charcoal, wood, straw/shrubs/grass, agricultural crop and animal dung) for cooking |
| Comparator | clean fuels (electricity, liquid petroleum gas (LPG), solar energy, natural gas and biogas) for cooking |

B1. Specify the outcome

Acute Respiratory Infection (ARI) in children

B2. If multiple outcomes presented, specify the numerical result being assessed

| **Confounding domains listed in step I** | | | |
| --- | --- | --- | --- |
| Confounding domain | Measured variable (s) | Is there evidence that  controlling for this Variable was unnecessary? | Is the confounding domain measured validly and reliably by this Variable (or these variables)? |
| Ambient air pollutants, Vaccination status, child nutritional status, breast feeding status, existing health condition (infected with HIV and TB), smoking (active and/or passive), crowding, low birth weight, and age of the child | Nutritional status | No | No information |
| **Additional confounding domains relevant to the setting of this particular study, or which the study authors identified as important** | | | |
| Sex of child, Birth order, Mother’s (age, education, religion and tribe), house construction material, presence of separate kitchen, standard of living, residence, region and family size | None | No | Yes |

**Step III: RoB across seven items**

| **Rec. No. EndNote_761** | | | |
| --- | --- | --- | --- |
| **Bias items** | **Risk of bias** | **Direction of bias** | **Rationale** |
| Bias due to confounder | Moderate |  | Authors controlled the following confounders: Environmental tobacco smoke, Age of child (in months), Sex of child, Birth order, Nutritional status of child, Mother’s (age, education, religion and tribe), house construction material, presence of separate kitchen, crowding, standard of living, residence, region and family size. A few important confounders (Ambient air pollutants, Vaccination status, breast feeding status, existing health condition (infected with HIV and TB), and low birth weight) missed to be controlled |
| Bias in selection of participants into the study | low |  | DHS implements probability sampling technique (selection of subjects was unrelated to either exposure or outcome status)  There is no means to commit bias in selection of participants. |
| Bias in classification of exposures | Serious | Towards the null | Authors include kerosene in comparator group. Additionally, There may be misclassification in categorizing participants into exposed to unclean fuel or not because some households could use a combination of unclean and clean fuels and information was only collected on primary fuel use in NFHS survey. The survey also did not account for past exposure to cooking fuel or recent changes in cooking methods. On top of these, authors considered kerosene under cleaner fuel category. |
| Bias due to deviations from intended exposures | Moderate |  | Even though there is high chance of exposure status change among participants, information was measured cross-sectional and analyzed from single measurement/interview. However, there is a chance of neighborhood and ambient air pollution from a household using polluted fuel and people might have used combination of solid fuels and cleaner fuel for cooking. Even though it is not unique to specific group, authors excluded ‘‘other fuels’’ category from their analysis. Another big concern could be the category of kerosene; as a cleaner fuel. |
| Bias due to missing data | Low |  | It is nationally representative data with very few missing information (98% response rate for households & 96% for women). |
| Bias in measurement of the outcome | Serious |  | It is subjective and ascertained by caregivers report and defined as “For children who had cough, the mother was additionally asked if the child, when sick with cough, breathed faster than usual with short, rapid breaths, any time during the last two weeks prior to the survey”. |
| Bias in selection of the reported result | Low |  | It is no way to report/present the report selectively. Statistics described under the method also reported in the result. Full data can be found in the two countries final NFHS report. |
| Overall bias | Serious |  | Mix of polluting and clean fuel used together as comparator; kerosene categorized under clean fuel. However, the miss classification affects the finding towards the null and it is based on large sample size |

**Study title:** **Mishra (2003):** Indoor air pollution from biomass combustion and acute respiratory illness in preschool age children in Zimbabwe

**Step II:** hypothetical target experiment, including specific confounders and co-interventions from the study that will require consideration

1. target randomized trial specific to the study

| Design | Individual randomized trial |
| --- | --- |
| Participants | Nationally representative Zimbabwe children aged 0–59 months included in the 1999 Zimbabwe Demographic and Health Survey (ZDHS) |
| Experimental intervention | polluting fuels (kerosene, coal/lignite, charcoal, wood, straw/shrubs/grass, agricultural crop and animal dung) for cooking |
| Comparator | clean fuels (electricity, liquid petroleum gas (LPG), solar energy, natural gas and biogas) for cooking |

B1. Specify the outcome

Acute Respiratory Infection (ARI) in children

B2. If multiple outcomes presented, specify the numerical result being assessed

| **Confounding domains listed in step I** | | | |
| --- | --- | --- | --- |
| Confounding domain | Measured variable (s) | Is there evidence that  controlling for this Variable was unnecessary? | Is the confounding domain measured validly and reliably by this Variable (or these variables)? |
| Ambient air pollutants, Vaccination status, child nutritional status, breast feeding status, existing health condition (infected with HIV and TB), smoking (active and/or passive), crowding, low birth weight, and age of the child | Nutritional status | No | No information |
| **Additional confounding domains relevant to the setting of this particular study, or which the study authors identified as important** | | | |
| gender of the child, birth order, mother's age at birth, education and religion, HH living standard and region | None | No | Yes |

**Step III: RoB across seven items**

| **Rec. No. EndNote_809** | | | |
| --- | --- | --- | --- |
| **Bias items** | **Risk of bias** | **Direction of bias** | **Rationale** |
| Bias due to confounder | Critical |  | Authors controlled the following confounders: age, nutritional status & gender of the child, birth order, mother's age at birth, mother’s educational status and her religion, HH living standard and region. There are many risk factors, which were missed to be controlled. |
| Bias in selection of participants into the study | low |  | DHS implements probability sampling technique (selection of subjects was unrelated to either exposure or outcome status)  There is no means to commit bias in selection of participants. |
| Bias in classification of exposures | Moderate |  | Authors categorized the exposed households in to two groups: HHs using high pollution fuels (wood, dung, or straw) and HHs using medium pollution fuels (kerosene or charcoal). There may be misclassification in categorizing participants into exposed to high pollution or medium polluting fuels because some households could use a combination of unclean and clean fuels and information was only collected on primary fuel use in DHS survey. The survey also did not account for past exposure to cooking fuel or recent changes in cooking methods. |
| Bias due to deviations from intended exposures | Moderate |  | Even though there is high chance of exposure status change among participants, information was measured cross-sectional and analyzed from single measurement/interview. However, there is a chance of neighborhood and ambient air pollution from a household using polluted fuel and people might have used combination of solid fuels and cleaner fuel for cooking. Even though it is small number, (0.1% of the sample) and not unique for one category, authors excluded households with unknown fuel category from the analysis. |
| Bias due to missing data | Low |  | It is nationally representative data with very few missing information (97.8% response rate for households) |
| Bias in measurement of the outcome | Serious |  | It is subjective and ascertained by caregivers report and defined as “For children who had cough, the mother was additionally asked if the child, when sick with cough, breathed faster than usual with short, rapid breaths, any time during the last two weeks prior to the survey”. |
| Bias in selection of the reported result | Low |  | It is no way to report/present the report selectively. Statistics described under the method also reported in the result. Full data can be found in the two countries final DHS report. |
| Overall bias | Critical |  | Only nutritional status and age of the child were the risk factors controlled by the authors. |

1. **Studies of mortality (neonatal, infant, child and under-five)**

**Step I**: review question, potential confounders, co-interventions, and exposure and outcome measurement accuracy information

| **Step I items** | **Response** |
| --- | --- |
| PECO question | Is there a difference in under-five mortality (neonatal, infant, child or under-five) risks between HHs using polluting fuels (kerosene, coal/lignite, charcoal, wood, straw/shrubs/grass, agricultural crop and animal dung) vs. HHs using clean fuels (electricity, liquid petroleum gas (LPG), natural gas and biogas, solar energy) for cooking? |
| Confounding for HAP and child mortality | Maternal age (giving birth at younger age), inter-birth interval (short inter-birth interval between births), low birth weight, malnutrition (specifically wasting), non-breastfed children, overcrowded conditions, unsafe drinking water, poor hygiene practices and household income |
| Co-interventions | - None identified |
| Accuracy of the measurement of exposure to  HAP | The following techniques are placed in descending order of exposure assessment accuracy.   1. Biological monitoring (collecting a sample of biomarkers from blood or urine samples) 2. Personal monitoring (placing a pollution monitor on an individual for a specified period) 3. Micro-environmental area-based monitoring (placement of the monitor near household pollution source). 4. Questionnaire/self-report (fuel/stove type, ventilation, cooking place and behaviour, etc.) |
| Accuracy of the measurement of outcome (mortality) | - Vital registration systems, population censuses, household surveys, sample registration systems and demographic surveillance sites are methods used to measure child mortality. - Vital registration data is the preferred source of data for child mortality if the system is well functioning |

**Part 1a**

**Title of the study: Samuel et al. (2018):** Household use of solid fuel for cooking and under-five mortality in Nigeria

**Step II:** hypothetical target experiment, including specific confounders and co-interventions from that study that will require consideration

1. target randomized trial specific to the study

| Design | Household randomized trial |
| --- | --- |
| Participants | Nationally representative Nigerian under-five children selected from households that were their kitchen located within the house during 2013 NDHS. Five years preceding the survey is the time limit for the children to be included in the analysis. |
| Experimental intervention | polluting fuels (kerosene, coal/lignite, charcoal, wood, straw/shrubs/grass, agricultural crop and animal dung) for cooking |
| Comparator | clean fuels (electricity, liquid petroleum gas (LPG), natural gas and biogas) for cooking |

B1. Specify the outcome

Under-five mortality

B2. If multiple outcomes presented, specify the numerical result being assessed

| **Confounding domains listed in step I** | | | |
| --- | --- | --- | --- |
| Confounding domain | Measured variable (s) | Is there evidence that  controlling for this  Variable was unnecessary? | Is the confounding domain measured  validly and reliably by this  Variable (or these variables)? |
| Maternal age, inter-birth interval, low birth weight, malnutrition, non-breastfed children, overcrowded conditions, unsafe drinking water, poor hygiene practices, household income | None | No | No information |
| **Additional confounding domains relevant to the setting of this particular study, or which the study authors identified as important** | | | |
| Residence, region, wealth status and mother’s educational status | None | No | Yes |

**Step III**: RoB across seven items

| **Rec. No. EndNote_2744** | | | |
| --- | --- | --- | --- |
| **Bias items** | **Risk of**  **bias** | **Direction of bias** | **Rationale** |
| Bias due to confounding | Critical |  | Authors controlled residence (urban/rural), region (north/south), wealth status and mother’s educational status.  Factors such as maternal age, inter-birth interval, childbirth weight, child nutritional status, child breast feeding status, malnutrition, crowing index and hygiene were not controlled. |
| Bias in selection of participants  into the study | low |  | DHS implements probability sampling technique (selection of subjects was unrelated to either exposure or outcome status)  There is no means to commit bias in selection of participants. |
| Bias in classification of  exposures | Serious | Towards the null | In their categorization, authors included kerosene under comparator group. According to their analysis, households using non-solid fuel (electric, gas and kerosene) in the kitchen inside the house were considered as a reference group where kerosene, the polluting fuel type considered in the group. |
| Bias due to deviations from intended exposures | Serious |  | Two things imposed potential bias on this regard: first, the DHS captured information existing only at the time of the survey (cross-sectional); there could be fuel use/choice pattern shift among households. When households economically become stronger, they tend to shift to cleaner fuel |
| Bias due to missing data | Low |  | The study has response rate of 97.6% (for women). In addition, it is unlikely that missing information is vary between the two (HHs with polluting fuel and HHs with clean fuel) groups. |
| Bias in measurement of  the outcome | Moderate |  | The method used to ascertain the outcome was relatively good (**see step I**), but recall bias on date and months of events could be inevitable because of the nature of the data acquisition technique. Including all-cause mortality will also include mortality outcomes that were not associated with HAP. |
| Bias in selection of the  reported result | Low |  | All the variables considered in the analysis found in the dataset and the descriptive result can be found in the final report, so that it is less likely to commit selection of the reported result. |
| Overall bias | Critical |  | Authors missed many confounding factors and categorised kerosene under clean fuel |

**Study title: Naz et al. (2018):** Potential Impacts of Modifiable Behavioral and Environmental Exposures on Reducing Burden of Under-five Mortality Associated with Household Air Pollution in Nepal

**Step II: hypothetical target experiment, including specific confounders and co-interventions from that study that will require consideration**

1. target randomized trial specific to the study

| Design | Household randomized trial |
| --- | --- |
| Participants | Nationally representative 17,780 singleton live-born under-five children of Nepal. The analysis restricted to under-five children belonging to the households selected by DHS program. Five years presiding the survey is the time limit for the children to be included in the analysis. |
| Experimental intervention | polluting fuels (kerosene, coal/lignite, charcoal, wood, straw/shrubs/grass, agricultural crop and animal dung) for cooking |
| Comparator | clean fuels (electricity, liquid petroleum gas (LPG), natural gas and biogas) for cooking |

B1. Specify the outcome

Under-five mortality

B2. If multiple outcomes presented, specify the numerical result being assessed

| 1. **Confounding domains listed in step I** | | | |
| --- | --- | --- | --- |
| Confounding domain | Measured variable (s) | Is there evidence that controlling for this variable was unnecessary? | Is the confounding domain measured validly and reliably by this variable (or these variables)? |
| Maternal age, inter-birth interval, low birth weight, malnutrition, non-breastfed children, overcrowded conditions, unsafe drinking water, poor hygiene practices, household income | None | No | No information |
| 1. **Additional confounding domains relevant to the setting of this particular study, or which the study authors identified as important** | | | |
| Place of residence, ecological zone, mother’s education, mother’s working status, floor material of household, parity, sex of the child and location of the kitchen | None | No | Yes |

Step III: RoB across seven items

| **Rec. No. EndNote_114** | | | |
| --- | --- | --- | --- |
| **Bias items** | **Risk of**  **bias** | **Direction of bias** | **Rationale** |
| Bias due to confounding | Serious |  | Critical confounders of HAP and under-five mortality such as inter-birth interval, low birth weight, malnutrition, overcrowding, unsafe drinking water and poor hygienic practices were not controlled.  Some of these variables collected in the DHS but with considerable number of missing information. The authors did not mentioned this. |
| Bias in selection of participants  into the study | low |  | DHS implements probability sampling technique (selection of subjects was unrelated to either exposure or outcome status)  There is no means to commit bias in selection of participants. |
| Bias in classification of  exposures | Moderate |  | There may be misclassification in categorising participants into exposed to polluting fuel or not because some households could use a combination of polluting and clean fuels and information was only collected on primary fuel use in DHS survey.  This study also did not account for past exposure to cooking fuel or recent changes in cooking methods. |
| Bias due to deviations from intended exposures | Moderate |  | The DHS captured information existing only at the time of the survey (cross-sectional); there could be fuel use/choice pattern shift among households. When households economically become stronger, they tend to shift to cleaner fuel. However, there is slow rate of shift to cleaner fuel |
| Bias due to missing data | Low |  | The study has response rate of 98.2%. In addition, it is unlikely that missing information is vary between the two (HHs with polluting fuel and HHs with clean fuel) groups. |
| Bias in measurement of the outcome | Moderate |  | The method used to ascertain the outcome was relatively good (**see step I**), but recall bias on date and months of events could be inevitable because of the nature of the data acquisition technique. Including all-cause mortality will also include mortality outcomes that were not associated with HAP. |
| Bias in selection of the  reported result | Low |  | All the variables considered in the analysis found in the dataset and the descriptive result can be found in the final report, so that it is less likely to commit selection of the reported result. Authors also assessed mortality at several different periods of children’s age and presented all the finds accordingly. Statistical methods reported in the methods section also presented in the results section. |
| Overall bias | Moderate |  | Some important confounders not controlled but large sample size and larger effect size minimize risk of bias |

**Title of the study: Owili et al. (2017):** Cooking fuel and risk of under-five mortality in 23 Sub-Saharan African countries: a population-based study

**Step II:** hypothetical target experiment, including specific confounders and co-interventions from that study that will require consideration

1. target randomized trial specific to the study

| Design | Household randomized trial |
| --- | --- |
| Participants | Nationally representative 23 Sub-Saharan African under-five children included in Demographic and Health Surveys collected between 2010 and 2014 DHS. |
| Experimental intervention | polluting fuels (kerosene, coal/lignite, charcoal, wood, straw/shrubs/grass, agricultural crop and animal dung) for cooking |
| Comparator | clean fuels (electricity, liquid petroleum gas (LPG), natural gas and biogas) for cooking |

B1. Specify the outcome

Under-five mortality

B2. If multiple outcomes presented, specify the numerical result being assessed

| **Confounding domains listed in step I** | | | |
| --- | --- | --- | --- |
| Confounding domain | Measured variable (s) | Is there evidence that  controlling for this  Variable was unnecessary? | Is the confounding domain measured  validly and reliably by this  Variable (or these variables)? |
| Maternal age, inter-birth interval, low birth weight, malnutrition, non-breastfed children, overcrowded conditions, unsafe drinking water, poor hygiene practices, household income | None | No | No information |
| **Additional confounding domains relevant to the setting of this particular study, or which the study authors identified as important** | | | |
| kitchen location, country, residence, child's gender, breast feeding status, mother's education and occupation, family size, number of U5, wealth index, HH member smoking status and father's occupation | None | No | Yes |

Step III: RoB across seven items

| **Rec. No. EndNote_152** | | | |
| --- | --- | --- | --- |
| **Bias items** | **Risk of**  **bias** | **Direction of bias** | **Rationale** |
| Bias due to confounding | Moderate |  | Authors controlled kitchen location, country, residence, child's gender, breast feeding status, mother's age, education and occupation, family size, number of U5, wealth index, HH member smoking status and father's occupation. Family size and number of under-five children in the house could represent the inter-birth interval and household crowding index. |
| Bias in selection of participants  into the study | low |  | DHS implements probability sampling technique (selection of subjects was unrelated to either exposure or outcome status)  There is no means to commit bias in selection of participants. |
| Bias in classification of  exposures | Moderate |  | Authors classified exposure variable, which are groups of polluting fuels in to three groups. These are 1. fuel used mainly indoor (charcoal) 2. fuel used mainly outdoor (wood, straw/shrubs/grass, agricultural crops, or animal dung) and 3. other polluting cooking fuel (coal, lignite or paraffin/kerosene). Such category could help to see the independent effect of each fuel.  However, some households could use a combination of polluting and clean fuels and information was only collected on primary fuel use in DHS survey.  This study also did not account for past exposure to cooking fuel or recent changes in cooking methods. |
| Bias due to deviations from intended exposures | Moderate |  | Authors assessed the effect of polluting fuels by categorizing in to three groups. However, the DHS captured information existing only at the time of the survey (cross-sectional); there could be fuel use/choice pattern shift among households. |
| Bias due to missing data | Low |  | The DHS is nationally representative survey, usually with >90% response rate. In addition this this, it is unlikely that missing information is vary between the two (HHs with polluting fuel and HHs with clean fuel) groups. |
| Bias in measurement of  the outcome | Moderate |  | The method used to ascertain the outcome was relatively good (**see step I**), but recall bias on date and months of events could be inevitable because of the nature of the data acquisition technique. Including all-cause mortality will also include mortality outcomes that were not associated with HAP. |
| Bias in selection of the  reported result | Low |  | All the variables considered in the analysis found in the dataset and the descriptive result can be found in the final report, so that it is less likely to commit selection of the reported result. |
| Overall bias | Moderate |  | The high sample size and fuel categories considered by authors are considerable strengths of the study |

**Title of the study: Naz et al. (2017):** Household air pollution from use of cooking fuel and under-five mortality: The role of breastfeeding status and kitchen location in Pakistan

**Step II:** hypothetical target experiment, including specific confounders and co-interventions from that study that will require consideration

1. target randomized trial specific to the study

| Design | Household randomized trial |
| --- | --- |
| Participants | Nationally representative Pakistan under-five children born singleton and included in Pakistan Demographic and Health Surveys collected in 2013. |
| Experimental intervention | polluting fuels (kerosene, coal/lignite, charcoal, wood, straw/shrubs/grass, agricultural crop and animal dung) for cooking |
| Comparator | clean fuels (electricity, liquid petroleum gas (LPG), natural gas and biogas) for cooking |

B1. Specify the outcome

Under-five mortality

B2. If multiple outcomes presented, specify the numerical result being assessed

| **Confounding domains listed in step I** | | | |
| --- | --- | --- | --- |
| Confounding domain | Measured variable (s) | Is there evidence that  controlling for this  Variable was unnecessary? | Is the confounding domain measured  validly and reliably by this  Variable (or these variables)? |
| Maternal age, inter-birth interval, low birth weight, malnutrition, non-breastfed children, overcrowded conditions, unsafe drinking water, poor hygiene practices, household income | None | No | No information |
| **Additional confounding domains relevant to the setting of this particular study, or which the study authors identified as important** | | | |
| place of residence, mother’s education, working and smoking status, floor & wall material of the house, sex of the child, location of the kitchen, age of the child | None | No | Yes |

Step III: RoB across seven items

| **Rec. No. EndNote_164** | | | |
| --- | --- | --- | --- |
| **Bias items** | **Risk of**  **bias** | **Direction of bias** | **Rationale** |
| Bias due to confounding | Serious |  | Authors controlled place of residence, household wealth index, mother’s education, working and smoking status, and her age, floor & wall material of the house, sex of the child, breast feeding status, location of the kitchen, age of the child. Some of these variables could be a risk factor for under-five mortality but authors missed a few important confounders such as wash and child’s nutritional status were not controlled. |
| Bias in selection of participants  into the study | low |  | DHS implements probability sampling technique (selection of subjects was unrelated to either exposure or outcome status)  There is no means to commit bias in selection of participants. |
| Bias in classification of  exposures | Moderate |  | Authors made correct classification of fuels in to clean and polluted sources. In addition, they also conducted sub-analysis to investigate the association between different fuel types with under-five mortality, which is helpful to see the independent effect of separate fuel while minimizing exposure classification bias. Notwithstanding of this, some households could use a combination of polluting and clean fuels and information was only collected on primary fuel use in DHS survey. This study also did not account for past exposure to cooking fuel or recent changes in cooking methods. |
| Bias due to deviations from intended exposures | Moderate |  | The DHS captured information existing only at the time of the survey (cross-sectional); there could be fuel use/choice pattern shift among households. When households economically become stronger, they tend to shift to cleaner fuel. |
| Bias due to missing data | Low |  | The analysis is based on 93.1% of response rate for women so that it has minimal missing data. In addition to this, the missed information could not be specific to one group. |
| Bias in measurement of  the outcome | Moderate |  | The method used to ascertain the outcome was relatively good (**see step I**), but recall bias on date and months of events could be inevitable because of the nature of the data acquisition technique. Including all-cause mortality will also include mortality outcomes that were not associated with HAP. |
| Bias in selection of the  reported result | Low |  | All the variables considered in the analysis found in the dataset and the descriptive result can be found in the final report, so that it is less likely to commit bias in selection of the reported result. |
| Overall bias | Moderate |  | Large sample size and |

**Title of the study: Khan et al. (2017):** Household air pollution from cooking and risk of adverse health and birth outcomes in Bangladesh: a nationwide population based study

**Step II:** hypothetical target experiment, including specific confounders and co-interventions from that study that will require consideration

1. target randomized trial specific to the study

| Design | Household randomized trial |
| --- | --- |
| Participants | Nationally representative Bangladesh under-five children included in BDHS collected in 2007, 2011 and 2014, live-born children within five years preceding the survey; only outcomes of the most recent births were considered |
| Experimental intervention | polluting fuels (kerosene, coal/lignite, charcoal, wood, straw/shrubs/grass, agricultural crop and animal dung) for cooking |
| Comparator | clean fuels (electricity, liquid petroleum gas (LPG), natural gas and biogas) for cooking |

B1. Specify the outcome

Under-five mortality

B2. If multiple outcomes presented, specify the numerical result being assessed

| **Confounding domains listed in step I** | | | |
| --- | --- | --- | --- |
| Confounding domain | Measured variable (s) | Is there evidence that  controlling for this  Variable was unnecessary? | Is the confounding domain measured  validly and reliably by this  Variable (or these variables)? |
| Maternal age, inter-birth interval, low birth weight, malnutrition, non-breastfed children, overcrowded conditions, unsafe drinking water, poor hygiene practices, household income | None | No | No information |
| **Additional confounding domains relevant to the setting of this particular study, or which the study authors identified as important** | | | |
| educational attainment, region of residence, place of residence and children’s gender | None | No | Yes |

Step III: RoB across seven items

| **Rec. No. EndNote_280** | | | |
| --- | --- | --- | --- |
| **Bias items** | **Risk of**  **bias** | **Direction of bias** | **Rationale** |
| Bias due to confounding | Serious |  | Authors controlled women’s age at birth, wealth quintiles, educational attainment, region of residence, place of residence and children’s gender. Other variables like children’s nutritional status, water and hygiene status were not considered as a confounders. |
| Bias in selection of participants  into the study | low |  | DHS implements probability sampling technique (selection of subjects was unrelated to either exposure or outcome status)  There is no means to commit bias in selection of participants. |
| Bias in classification of  exposures | Serious |  | Authors classified cooking fuel in to two groups: solid and clean fuel. Even though kerosene is one of the fuel options in the category, authors have not mentioned what happened to kerosene; excluded or not.  In addition to this, some households could use a combination of solid and clean fuels, but this is not addressed in the survey. Furthermore, the study also did not account for past exposure to cooking fuel or recent changes in cooking methods. |
| Bias due to deviations from intended exposures | Moderate |  | The DHS is cross-sectional; there could be fuel use/choice pattern shift among households at some point in the past time. When households economically become stronger, they tend to shift to cleaner fuel. |
| Bias due to missing data | Low |  | The analysis is based on nationally representative sample, which 98% combined response rate. Yet again, missed information could be less likely to be specific to one category. |
| Bias in measurement of  the outcome | Moderate |  | The method used to ascertain the outcome was relatively good (**see step I**), but recall bias on date and months of events could be inevitable because of the nature of the data acquisition technique. Including all-cause mortality will also include mortality outcomes that were not associated with HAP. |
| Bias in selection of the  reported result | Low |  | All the variables considered in the analysis found in the dataset and the descriptive result can be found in the final report, so that it is less likely to commit bias in selection of the reported result. |
| Overall bias | Serious |  | Many risk factors of child mortality missed to be controlled and even though kerosene is found in the fuel type, it is not mentioned |

**Title of the study: Naz et al. (2016):** Household air pollution and under-five mortality in India (1992–2006)

**Step II:** hypothetical target experiment, including specific confounders and co-interventions from that study that will require consideration

1. target randomized trial specific to the study

| Design | Household randomized trial |
| --- | --- |
| Participants | Nationally representative under-five Indian children that were included in India’s National Family and Health Survey (NFHS), which were conducted in the years 1992–93 (NFHS-1), 1998–99 (NFHS-2) and 2005–06 (NFHS-3). |
| Experimental intervention | polluting fuels (kerosene, coal/lignite, charcoal, wood, straw/shrubs/grass, agricultural crop and animal dung) for cooking |
| Comparator | clean fuels (electricity, liquid petroleum gas (LPG), natural gas and biogas) for cooking |

B1. Specify the outcome

Under-five mortality

B2. If multiple outcomes presented, specify the numerical result being assessed

| **Confounding domains listed in step I** | | | |
| --- | --- | --- | --- |
| Confounding domain | Measured variable (s) | Is there evidence that  controlling for this  Variable was unnecessary? | Is the confounding domain measured  validly and reliably by this  Variable (or these variables)? |
| Maternal age  Inter-birth interval  Low birth weight Malnutrition Non-breastfed children  Overcrowded conditions  Unsafe drinking water  Poor hygiene practices  Household income | None | No | No information |
| **Additional confounding domains relevant to the setting of this particular study, or which the study authors identified as important** | | | |
| place of residence, household wealth index, mother’s education, working and smoking status, and her age, type of house, breast feeding status, presence of separate kitchen, age of the child, survey year | None | No | Yes |

Step III: RoB across seven items

| **Rec. No. EndNote_265** | | | |
| --- | --- | --- | --- |
| **Bias items** | **Risk of**  **bias** | **Direction of bias** | **Rationale** |
| Bias due to confounding | Serious |  | Authors controlled place of residence, household wealth index, mother’s education, working and smoking status, and her age, floor & wall material of the house, sex of the child, breast feeding status, presence of separate kitchen, age of the child. Some of these variables could be a risk factor for under-five mortality but authors missed a few important confounders such as wash and child’s nutritional status were not controlled. |
| Bias in selection of participants  into the study | low |  | DHS implements probability sampling technique (selection of subjects was unrelated to either exposure or outcome status)  There is no means to commit bias in selection of participants. |
| Bias in classification of  exposures | Moderate |  | Authors made correct classification of fuels in to clan and polluted sources. In addition, they also conducted sub-analysis to investigate the association between different fuel types with under-five mortality, which is helpful to see the independent effect of separate fuel while minimizing exposure classification bias. Furthermore, they conducted sensitivity analysis by including kerosene in a clean fuel group; they found no change in the result. Notwithstanding of this, some households could use a combination of polluting and clean fuels and information was only collected on primary fuel use in Indian NFHS. This study also did not account for past exposure to cooking fuel or recent changes in cooking methods. |
| Bias due to deviations from intended exposures | Moderate |  | The Indian NFHS captured information existing only at the time of the survey (cross-sectional); there could be fuel use/choice pattern shift among households. When households economically become stronger, they tend to shift to cleaner fuel. |
| Bias due to missing data | Low |  | The analysis is based on 95.4% of composite response rate for women in the three surveys so that it has minimal missing data. In addition to this, the missed information could not be specific to one group. |
| Bias in measurement of  the outcome | Moderate |  | The method used to ascertain the outcome was relatively good (**see step I**), but recall bias on date and months of events could be inevitable because of the nature of the data acquisition technique. Including all-cause mortality will also include mortality outcomes that were not associated with HAP. |
| Bias in selection of the  reported result | Low |  | All the variables considered in the analysis found in the dataset and the descriptive result can be found in the final report, so that it is less likely to commit bias in selection of the reported result. |
| Overall bias | Moderate |  | Despite these methodological concerns, the study used large-scale nationally representative DHS data with a very high response rate of 95.4 %, and high sample size. The fuel categories considered by authors is also strengths of the study |

**Title of the study: Akinyemi et al. (2016):** Independent and combined effects of maternal smoking and solid fuel on infant and child mortality in sub-Saharan Africa

**Step II:** hypothetical target experiment, including specific confounders and co-interventions from that study that will require consideration

1. target randomized trial specific to the study

| Design | Household randomized trial |
| --- | --- |
| Participants | Nationally representative infants and children from 15 Sub-Saharan African Countries born prior to Demographic and Health Surveys (DHS) conducted between 2010 to 2014 |
| Experimental intervention | polluting fuels (kerosene, coal/lignite, charcoal, wood, straw/shrubs/grass, agricultural crop and animal dung) for cooking |
| Comparator | clean fuels (electricity, liquid petroleum gas (LPG), natural gas and biogas) for cooking |

B1. Specify the outcome

Under-five mortality

B2. If multiple outcomes presented, specify the numerical result being assessed

| **Confounding domains listed in step I** | | | |
| --- | --- | --- | --- |
| Confounding domain | Measured variable (s) | Is there evidence that  controlling for this  Variable was unnecessary? | Is the confounding domain measured  validly and reliably by this  Variable (or these variables)? |
| Maternal age, inter-birth interval, low birth weight, malnutrition, non-breastfed children, overcrowded conditions, unsafe drinking water, poor hygiene practices, household income | None | No | No information |
| **Additional confounding domains relevant to the setting of this particular study, or which the study authors identified as important** | | | |
| mother's education, occupation, smoking satus & access to media, other family smoking status, place of residence, birth order, sex of child & wealth index | None | No | Yes |

Step III: RoB across seven items

| **Rec. No. EndNote_2387** | | | |
| --- | --- | --- | --- |
| **Bias items** | **Risk of**  **bias** | **Direction of bias** | **Rationale** |
| Bias due to confounding | Low |  | Authors controlled variables related to mother (age, education, occupation smoking status and access to media), other family smoking status, place of residence, birth order, sex of child, child's size at birth, birth interval, wealth index and HH size. Child’s size at birth and HH size could replace weight at birth and household crowding index, respectively. Other variables such as child’s nutritional status and wash status are not considered. |
| Bias in selection of participants  into the study | low |  | DHS implements probability sampling technique (selection of subjects was unrelated to either exposure or outcome status)  There is no means to commit bias in selection of participants. |
| Bias in classification of  exposures | Serious |  | Authors classified cooking fuel in to two groups; solid and non-solid fuel. They considered non-solid fuel as less exposed group where kerosene considered under this group. What is more, some households could use a combination of polluting and clean fuels and information was only collected on primary fuel use in DHS survey. This study also did not account for past exposure to cooking fuel or recent changes in cooking methods. |
| Bias due to deviations from intended exposures | Moderate |  | The DHS captured information existing only at the time of the survey (cross-sectional); there could be fuel use/choice pattern shift among households. When households economically become stronger, they tend to shift to cleaner fuel. |
| Bias due to missing data | Low |  | The analysis is based on nationally representative sample, which usually yields >90% response rate. Yet again, missed information could be less likely to be specific to one category. |
| Bias in measurement of  the outcome | Moderate |  | The method used to ascertain the outcome was relatively good (**see step I**), but recall bias on date and months of events could be inevitable because of the nature of the data acquisition technique. Including all-cause mortality will also include mortality outcomes that were not associated with HAP. |
| Bias in selection of the  reported result | Low |  | All the variables considered in the analysis found in the dataset and the descriptive result can be found in the final report, so that it is less likely to commit bias in selection of the reported result. |
| Overall bias | Serious |  | Authors categorised kerosene as a clean fuel |

**Title of the study: Naz et al. (2015):** Household Air Pollution and Under-Five Mortality in Bangladesh (2004–2011)

**Step II:** hypothetical target experiment, including specific confounders and co-interventions from that study that will require consideration

1. target randomized trial specific to the study

| Design | Household randomized trial |
| --- | --- |
| Participants | Nationally representative singleton under-five Bangladesh children included in Bangladesh Demographic and Health Surveys (DHS) conducted during 2004, 2007 & 2011 |
| Experimental intervention | polluting fuels (kerosene, coal/lignite, charcoal, wood, straw/shrubs/grass, agricultural crop and animal dung) for cooking |
| Comparator | clean fuels (electricity, liquid petroleum gas (LPG), natural gas and biogas) for cooking |

B1. Specify the outcome

Under-five mortality

B2. If multiple outcomes presented, specify the numerical result being assessed

| **Confounding domains listed in step I** | | | |
| --- | --- | --- | --- |
| Confounding domain | Measured variable (s) | Is there evidence that  controlling for this  Variable was unnecessary? | Is the confounding domain measured  validly and reliably by this  Variable (or these variables)? |
| Maternal age, inter-birth interval, low birth weight, malnutrition, non-breastfed children, overcrowded conditions, unsafe drinking water, poor hygiene practices, household income | None | No | No information |
| **Additional confounding domains relevant to the setting of this particular study, or which the study authors identified as important** | | | |
| place of residence, mother’s education, mother’s working status, wall material of house and survey year | None | No | Yes |

Step III: RoB across seven items

| **Rec. No. EndNote_280** | | | |
| --- | --- | --- | --- |
| **Bias items** | **Risk of**  **bias** | **Direction of bias** | **Rationale** |
| Bias due to confounding | Serious |  | Authors controlled household wealth status, place of residence, mother’s age, mother’s education and her working status, breastfeeding status, wall material of house, household wealth and survey year. Under-five risk factors such as inter-birth interval, birth weight, nutritional status of the child and wash status were not controlled. |
| Bias in selection of participants  into the study | low |  | DHS implements probability sampling technique (selection of subjects was unrelated to either exposure or outcome status)  There is no means to commit bias in selection of participants. |
| Bias in classification of  exposures | Moderate |  | Authors classified cooking fuel in to two groups; polluting and clean. The polluted fuel category included kerosene, wood, animal dung, charcoal, coal, and shrubs/ grass/straw. Natural gas, LPG, biogas and electricity are the elements of cleaner fuel group. Even though the categories are plausible, some households could use a combination of polluting and clean fuels and information was only collected on primary fuel use in DHS surveys. This study also did not account for past exposure to cooking fuel or recent changes in cooking methods. |
| Bias due to deviations from intended exposures | Moderate |  | The DHS captured information existing only at the time of the survey (cross-sectional); there could be fuel use/choice pattern shift among households. When households economically become stronger, they tend to shift to cleaner fuel. |
| Bias due to missing data | Low |  | The analysis is based on nationally representative sample, which 98.3% combined response rate. Yet again, missed information could be less likely to be specific to one category. |
| Bias in measurement of  the outcome | Moderate |  | The method used to ascertain the outcome was relatively good (**see step I**), but recall bias on date and months of events could be inevitable because of the nature of the data acquisition technique. Including all-cause mortality will also include mortality outcomes that were not associated with HAP. |
| Bias in selection of the  reported result | Low |  | All the variables considered in the analysis found in the dataset and the descriptive result can be found in the final report, so that it is less likely to commit bias in selection of the reported result. |
| Overall bias | Moderate |  | Even though authors missed many risk factors in their analysis, large sample size minimize the risk resalted from confounding effect. |

**Title of the study: Kleimola et al. (2015):** Consequences of household air pollution on child survival: evidence from demographic and health surveys in 47 countries

**Step II:** hypothetical target experiment, including specific confounders and co-interventions from that study that will require consideration

1. target randomized trial specific to the study

| Design | Household randomized trial |
| --- | --- |
| Participants | Nationally representative under-five children from 47 countries (from four global regions: Americas, Asia, North Africa and Europe, and SSA) included in Demographic and Health Surveys (DHS) conducted between 2001 and 2012, only singleton births living with their birth mothers at the house surveyed. |
| Experimental intervention | polluting fuels (kerosene, coal/lignite, charcoal, wood, straw/shrubs/grass, agricultural crop and animal dung) for cooking |
| Comparator | clean fuels (electricity, liquid petroleum gas (LPG), natural gas and biogas) for cooking |

B1. Specify the outcome

Under-five mortality

B2. If multiple outcomes presented, specify the numerical result being assessed

| **Confounding domains listed in step I** | | | |
| --- | --- | --- | --- |
| Confounding domain | Measured variable (s) | Is there evidence that  controlling for this  Variable was unnecessary? | Is the confounding domain measured  validly and reliably by this  Variable (or these variables)? |
| Maternal age, inter-birth interval, low birth weight, malnutrition, non-breastfed children, overcrowded conditions, unsafe drinking water, poor hygiene practices, household income | None | No | No information |
| **Additional confounding domains relevant to the setting of this particular study, or which the study authors identified as important** | | | |
| sex, birth order, mother’s education, whether or not she currently smokes cigarettes, urban versus rural residence, and country | None | No | Yes |

Step III: RoB across seven items

| **Rec. No. EndNote_281** | | | |
| --- | --- | --- | --- |
| **Bias items** | **Risk of**  **bias** | **Direction of bias** | **Rationale** |
| Bias due to confounding | Critical |  | sex, birth order, mother’s education, mother’s age at birth, whether or not she currently smokes cigarettes, household wealth index, urban versus rural residence, and country are variables controlled by authors as a confounder. Authors excluded birth weight as a covariate they mentioned because it is likely on the causal pathway between exposure to HAP and mortality. |
| Bias in selection of participants  into the study | low |  | DHS implements probability sampling technique (selection of subjects was unrelated to either exposure or outcome status)  There is no means to commit bias in selection of participants. |
| Bias in classification of  exposures | Moderate |  | Authors analysed the impact of kerosene and other solid fuel separately. This could be important to appreciate the independent effects caused due to these fuels. On the other hand, some households could use a combination of polluting and clean fuels and information collected only on primary fuel use in DHS surveys so that it is impossible to overcome all exposure classification biases. This study also did not account for past exposure to cooking fuel or recent changes in cooking methods. |
| Bias due to deviations from intended exposures | Moderate |  | The DHS captured information existing only at the time of the survey (cross-sectional); there could be fuel use/choice pattern shift among households. When households economically become stronger, they tend to shift to cleaner fuel. |
| Bias due to missing data | Low |  | The analysis is based on nationally representative data with more than 95% response rate for HHs and more than 90% for women respondents. Again, missed information could be less likely to be specific to one category. |
| Bias in measurement of  the outcome | Moderate |  | The method used to ascertain the outcome was relatively good (**see step I**), but recall bias on date and months of events could be inevitable because of the nature of the data acquisition technique. Including all-cause mortality will also include mortality outcomes that were not associated with HAP. |
| Bias in selection of the  reported result | Low |  | All the variables considered in the analysis found in the dataset and the descriptive result can be found in the final report, so that it is less likely to commit bias in selection of the reported result. |
| Overall bias | Critical |  | Despite the analysis was based on large sample size, many risk factors of child mortality missed to be controlled. However, authors considered other confounders, which could be a risk in specific area/country |

**Title of the study: Ezeh et al (2014):** The effect of solid fuel use on childhood mortality in Nigeria: evidence from the 2013 cross-sectional household survey

**Step II:** hypothetical target experiment, including specific confounders and co-interventions from that study that will require consideration

1. target randomized trial specific to the study

| Design | Household randomized trial |
| --- | --- |
| Participants | Nationally representative Nigerian under-five children, singleton livebirths reported for the 5-year period prior to the interview date in 2013 NDHS included in the cohort. |
| Experimental intervention | polluting fuels (kerosene, coal/lignite, charcoal, wood, straw/shrubs/grass, agricultural crop and animal dung) for cooking |
| Comparator | clean fuels (electricity, liquid petroleum gas (LPG), natural gas and biogas) for cooking |

B1. Specify the outcome

Under-five mortality

B2. If multiple outcomes presented, specify the numerical result being assessed

| **Confounding domains listed in step I** | | | |
| --- | --- | --- | --- |
| Confounding domain | Measured variable (s) | Is there evidence that  controlling for this  Variable was unnecessary? | Is the confounding domain measured  validly and reliably by this  Variable (or these variables)? |
| Maternal age, inter-birth interval, low birth weight, malnutrition, non-breastfed children, overcrowded conditions, unsafe drinking water, poor hygiene practices, household income | None | No | No information |
| **Additional confounding domains relevant to the setting of this particular study, or which the study authors identified as important** | | | |
| Gender of the child, educational status, mother’s working status, residence (urban/rural), and kitchen location | None | No | Yes |

Step III: RoB across seven items

| **Rec. No. EndNote_394** | | | |
| --- | --- | --- | --- |
| **Bias items** | **Risk of**  **bias** | **Direction of bias** | **Rationale** |
| Bias due to confounding | Moderate |  | Authors missed controlling a few important confounders (inter-birth interval, child’s nutritional status, crowding index and hygiene). Instead, they considered additional variables such as gender of the child, mother’s educational & working status, her residence (urban/rural), and kitchen location, which could be contextually a risk for child mortality. Authors also excluded multiple births because “compared with singletons, multiple births are associated with higher childhood mortality”. |
| Bias in selection of participants into the study | low |  | DHS implements probability sampling technique (selection of subjects was unrelated to either exposure or outcome status)  There is no means to commit bias in selection of participants. |
| Bias in classification of  exposures | Serious | Towards the null | There may be misclassification in categorising participants into exposed to polluting fuel or not because some households could use a combination of polluting and clean fuels and information was only collected on primary fuel use in DHS survey. Authors also stated that “data on households that use a combination of solid and non-solid fuels were not available from the NDHS database, and misclassification of use of cooking fuels may have occurred”  This study also did not account for past exposure to cooking fuel or recent changes in cooking methods.  Authors also categorized HHs in to polluting Vs clean fuel group. According to their analysis, households using non-solid fuel (electric, gas and kerosene) in the kitchen inside the house were considered as a reference group where kerosene, the polluted fuel type considered in the group. |
| Bias due to deviations from intended exposures | Serious |  | Two things imposed potential bias on this regard: first, the DHS captured information existing only at the time of the survey (cross-sectional); there could be fuel use/choice pattern shift among households. When households economically become stronger, they tend to shift to cleaner fuel |
| Bias due to missing data | Low |  | The study has response rate of 97.6% (for women). In addition, it is unlikely that missing information is vary between the two (HHs with polluting fuel and HHs with clean fuel) groups. |
| Bias in measurement of  the outcome | Moderate |  | The method used to ascertain the outcome was relatively good (**see step I**), but recall bias on date and months of events could be inevitable because of the nature of the data acquisition technique. Including all-cause mortality will also include mortality outcomes that were not associated with HAP. |
| Bias in selection of the  reported result | Low |  | All the variables considered in the analysis found in the dataset and the descriptive result can be found in the final report, so that it is less likely to commit selection of the reported result. |
| Overall bias | Critical |  | Authors classified kerosene, which is polluting fuel under clean fuel category |

**Title of the study: Pandey et al. (2013):** Adjusted Effects of Domestic Violence, Tobacco use, and Indoor Air Pollution from Use of Solid Fuel on Child Mortality

**Step II:** hypothetical target experiment, including specific confounders and co-interventions from that study that will require consideration

1. target randomized trial specific to the study

| Design | Household randomized trial |
| --- | --- |
| Participants | Nationally representative under-five Indian children that were included in India’s National Family and Health Survey (NFHS), conducted in the years 2005–06 (NFHS), included in the couples file where couples experienced pregnancy or child birth. |
| Experimental intervention | polluting fuels (kerosene, coal/lignite, charcoal, wood, straw/shrubs/grass, agricultural crop and animal dung) for cooking |
| Comparator | clean fuels (electricity, liquid petroleum gas (LPG), natural gas and biogas) for cooking |

B1. Specify the outcome

Under-five mortality

B2. If multiple outcomes presented, specify the numerical result being assessed

| **Confounding domains listed in step I** | | | |
| --- | --- | --- | --- |
| Confounding domain | Measured variable (s) | Is there evidence that  controlling for this  Variable was unnecessary? | Is the confounding domain measured  validly and reliably by this  Variable (or these variables)? |
| Maternal age, inter-birth interval, low birth weight, malnutrition, non-breastfed children, overcrowded conditions, unsafe drinking water, poor hygiene practices, household income | None | No | No information |
| **Additional confounding domains relevant to the setting of this particular study, or which the study authors identified as important** | | | |
| tobacco use (mother & father), husband control, husband humiliates, husband physical abuse, mother's & father's education, mother's working status, religion, residence, presence of window, cook under chimney | None | No | Yes |

Step III: RoB across seven items

| **Rec. No. EndNote_468** | | | |
| --- | --- | --- | --- |
| **Bias items** | **Risk of**  **bias** | **Direction of bias** | **Rationale** |
| Bias due to confounding | Critical |  | Authors controlled for the following variables: tobacco use (mother & father), husband control, husband humiliates, husband physical abuse, mother's & father's education, mother's working status, mother’s age at first birth, religion, wealth index, residence, presence of window, and cook under chimney. It was justified that these variables are determinants of child mortality in the country. However, other important risk factors left uncontrolled. |
| Bias in selection of participants  into the study | low |  | DHS implements probability sampling technique (selection of subjects was unrelated to either exposure or outcome status)  There is no means to commit bias in selection of participants. |
| Bias in classification of  exposures | Serious | Towards the null | Authors categorised fuel type in to two groups: HHs using solid fuel (coal/ lignite, charcoal, wood, straw, shrubs, grass, agricultural crop waste, dung cake) and HHs using other fuels (electricity, LPG/Natural gas, biogas or Kerosene). Here, authors grouped kerosene under clean fuel category, which is miss classification. In addition, some households could use a combination of polluting and clean fuels and information collected only on primary fuel use in DHS surveys so that it is impossible to overcome all exposure classification biases. This study also did not account for past exposure to cooking fuel or recent changes in cooking methods. |
| Bias due to deviations from intended exposures | Moderate |  | The DHS captured information existing only at the time of the survey (cross-sectional); there could be fuel use/choice pattern shift among households. When households economically become stronger, they tend to shift to cleaner fuel. |
| Bias due to missing data | Low |  | It is unlikely to commit bias due to missing data because the analysis is based on nationally representative data and again, missed information could be less likely to be specific to one category. |
| Bias in measurement of  the outcome | Moderate |  | The method used to ascertain the outcome was relatively good (**see step I**), but recall bias on date and months of events could be inevitable because of the nature of the data acquisition technique. Including all-cause mortality will also include mortality outcomes that were not associated with HAP. |
| Bias in selection of the  reported result | Low |  | All the variables considered in the analysis found in the dataset and the descriptive result can be found in the final report, so that it is less likely to commit bias in selection of the reported result. |
| Overall bias | Critical |  | Authors missed controlling some important confounders and they categorised kerosene under clean fuel group. |

**Title of the study: Epstein et al. (2013):** Household fuels, low birth weight, and neonatal death in India: The separate impacts of biomass, kerosene, and coal

**Step II:** hypothetical target experiment, including specific confounders and co-interventions from that study that will require consideration

1. target randomized trial specific to the study

| Design | Household randomized trial |
| --- | --- |
| Participants | Nationally representative Indian mother-infant pair of singleton birth included in Indian National Family Health Survey conducted in 2005-6 NFHS. |
| Experimental intervention | HHs using polluting fuels (kerosene, coal/lignite, charcoal, wood, straw/shrubs/grass, agricultural crop and animal dung) |
| Comparator | HHs clean fuels (electricity, liquid petroleum gas (LPG), natural gas and biogas) |

B1. Specify the outcome

Birth weight

B2. If multiple outcomes presented, specify the numerical result being assessed

| **Confounding domains listed in step I** | | | |
| --- | --- | --- | --- |
| Confounding domain | Measured variable (s) | Is there evidence that  controlling for this  Variable was unnecessary? | Is the confounding domain measured  validly and reliably by this  Variable (or these variables)? |
| Maternal age, inter-birth interval, low birth weight, malnutrition, non-breastfed children, overcrowded conditions, unsafe drinking water, poor hygiene practices, household income | Maternal BMI & and anaemia | No | No information |
| **Additional confounding domains relevant to the setting of this particular study, or which the study authors identified as important** | | | |
| Infant gender, mother’s religion, occupation, Region, residence, HH access to electricity, Type of house, HH water source, mother’s perception for medical care and parity | None | No | Yes |

Step III: RoB across seven items

| **Rec. No. EndNote_429** | | | |
| --- | --- | --- | --- |
| **Bias items** | **Risk of**  **bias** | **Direction of bias** | **Rationale** |
| Bias due to confounding | Moderate |  | Authors controlled Infant gender, mother's age, education, religion, smoking status, occupation, BMI, region, residence, HH access to electricity, type of house, HH water source, mother’s perception for medical care, No. of ANC, parity and prior inter-birth interval. Additionally, they considered only singleton birth. |
| Bias in selection of participants into the study | low |  | DHS implements probability sampling technique (selection of subjects was unrelated to either exposure or outcome status)  There is no means to commit bias in selection of participants. |
| Bias in classification of  exposures | Moderate |  | Authors analysed the effect of coal, kerosene, charcoal and biomass fuel on birth weight, independently. They excluded electricity from comparator; used only LPG, biogas and natural gas. However, there may be misclassification in categorising participants into exposed to polluting fuel or not because some households could use a combination of polluting and clean fuels and information was only collected on primary fuel use in the survey.  This study also did not account for past exposure to cooking fuel or recent changes in cooking methods. |
| Bias due to deviations from intended exposures | Moderate |  | Survey captured information present only at the time of data collection (cross-sectional); there could be fuel use/choice pattern shift among households. Different studies portrayed that when households economically become stronger; they tend to shift to cleaner fuel.  However, the trend to shift in to cleaner fuel such as clean gases and electricity is minimal and very slow. |
| Bias due to missing data | Low |  | The study is based on nationally representative sample with 94.5% response rate. Furthermore, it is unlikely that missing information is vary between the two (HHs with polluting fuel and HHs with clean fuel) groups. |
| Bias in measurement of the outcome | Moderate |  | The method used to ascertain the outcome was relatively good (**see step I**), but recall bias on date and months of events could be inevitable because of the nature of the data acquisition technique. Including all-cause mortality will also include mortality outcomes that were not associated with HAP. |
| Bias in selection of the  reported result | Low |  | Authors reported all what was planned in the method, including the statistics mentioned. |
| Overall bias | Moderate |  | Authors controlled most of risk factors for child mortality. |

**Title of the study: Wichmann et al (2006):** Influence of Cooking and Heating Fuel Use on 1–59 Month Old Mortality in South Africa

**Step II:** hypothetical target experiment, including specific confounders and co-interventions from that study that will require consideration

1. target randomized trial specific to the study

| Design | Household randomized trial |
| --- | --- |
| Participants | Nationally representative South African 1-59 months of age children, born single, who live with their mother, born from African/Black ethnic group mothers included in SADHS Conducted in 1998. |
| Experimental intervention | HHs using polluting fuels (kerosene, coal/lignite, charcoal, wood, straw/shrubs/grass, agricultural crop and animal dung) |
| Comparator | HHs clean fuels (electricity, liquid petroleum gas (LPG), natural gas and biogas) |

B1. Specify the outcome

Birth weight

B2. If multiple outcomes presented, specify the numerical result being assessed

| **Confounding domains listed in step I** | | | |
| --- | --- | --- | --- |
| Confounding domain | Measured variable (s) | Is there evidence that  controlling for this  Variable was unnecessary? | Is the confounding domain measured  validly and reliably by this  Variable (or these variables)? |
| Maternal age, inter-birth interval, low birth weight, malnutrition, non-breastfed children, overcrowded conditions, unsafe drinking water, poor hygiene practices, household income | Child nutritional status | No | No information |
| **Additional confounding domains relevant to the setting of this particular study, or which the study authors identified as important** | | | |
| Child (Age (months), sex, year of birth | None | No | Yes |

Step III: RoB across seven items

| **Rec. No. EndNote_429** | | | |
| --- | --- | --- | --- |
| **Bias items** | **Risk of**  **bias** | **Direction of bias** | **Rationale** |
| Bias due to confounding | Low |  | Authors controlled Child (Age (months), sex, BF status, nutritional status & birth order), maternal age, preceding birth interval, type of toilet, water source, asset index, crowding & year of birth |
| Bias in selection of participants into the study | low |  | DHS implements probability sampling technique (selection of subjects was unrelated to either exposure or outcome status)  There is no means to commit bias in selection of participants. |
| Bias in classification of exposures | Moderate |  | Authors considered HHs using polluting fuels (wood, dung, coal or paraffin was used in the fuel combination without using liquid petroleum gas (LPG)/natural gas or electricity) as exposed group and HHs using clean fuels (LPG/natural gas or electricity, exclusively) as comparator group. However, there may be misclassification in categorising participants into exposed to polluting fuel or not because some households could use a combination of polluting and clean fuels and information was only collected on primary fuel use in the survey.  This study also did not account for past exposure to cooking fuel or recent changes in cooking methods. |
| Bias due to deviations from intended exposures | Moderate |  | Survey captured information present only at the time of data collection (cross-sectional); there could be fuel use/choice pattern shift among households. Different studies portrayed that when households economically become stronger; they tend to shift to cleaner fuel.  However, the trend to shift in to cleaner fuel such as clean gases and electricity is minimal and very slow. |
| Bias due to missing data | Low |  | The study is based on nationally representative sample with 97% and 92% response rate for HH and women interviewed, respectively. Furthermore, it is unlikely that missing information is vary between the two (HHs with polluting fuel and HHs with clean fuel) groups. |
| Bias in measurement of  the outcome | Moderate |  | The method used to ascertain the outcome was relatively good (**see step I**), but recall bias on date and months of events could be inevitable because of the nature of the data acquisition technique. Including all-cause mortality will also include mortality outcomes that were not associated with HAP. |
| Bias in selection of the  reported result | Low |  | Authors reported all what was planned in the method, including the statistics mentioned. |
| Overall bias | Moderate |  | Authors controlled most of risk factors for child mortality |

**Title of the study: Nisha et al. (2018):** Variations in perinatal mortality associated with different polluting fuel types and kitchen location in Bangladesh

**Step II:** hypothetical target experiment, including specific confounders and co-interventions from that study that will require consideration

1. target randomized trial specific to the study

| Design | Household randomized trial |
| --- | --- |
| Participants | Nationally representative 7 months and above singleton pregnancies and early neonates (ages of 0 and 6 days) sampled in Bangladesh DHS, which were conducted between 2004 to 2014. |
| Experimental intervention | polluting fuels (kerosene, coal/lignite, charcoal, wood, straw/shrubs/grass, agricultural crop and animal dung) for cooking |
| Comparator | clean fuels (electricity, liquid petroleum gas (LPG), natural gas and biogas) for cooking |

B1. Specify the outcome

Perinatal mortality

B2. If multiple outcomes presented, specify the numerical result being assessed

| **Confounding domains listed in step I** | | | |
| --- | --- | --- | --- |
| Confounding domain | Measured variable (s) | Is there evidence that  controlling for this  Variable was unnecessary? | Is the confounding domain measured  validly and reliably by this  Variable (or these variables)? |
| **Risk factors for early neonatal death**  Preterm birth, intrapartum complications, congenital disorders, sepsis, pneumonia and injuries | None | No | No information |
| **Additional confounding domains relevant to the setting of this particular study, or which the study authors identified as important** | | | |
| maternal education, birth order, place of residence, maternal working status during survey, kitchen location and year of survey | None | No | Yes |

Step III: RoB across seven items

| **Rec. No. EndNote_74** | | | |
| --- | --- | --- | --- |
| **Bias items** | **Risk of**  **bias** | **Direction of bias** | **Rationale** |
| Bias due to confounding | Critical |  | Authors considered the following variables as a covariate: Maternal age at birth, maternal education, birth order, maternal body mass index (BMI), place of residence, wealth index, maternal working status during survey, kitchen location and year of survey. Some of these variables could be risk for still birth/early neonatal death. They keep these covariates identical for both outcomes. Myriads of variables that could affect both outcomes were not controlled. |
| Bias in selection of participants into the study | low |  | DHS implements probability sampling technique (selection of subjects was unrelated to either exposure or outcome status)  There is no means to commit bias in selection of participants. |
| Bias in classification of  exposures | Moderate |  | There may be misclassification in categorising participants into exposed to polluting fuel or not because some households could use a combination of polluting and clean fuels and information was only collected on primary fuel use in DHS survey.  This study also did not account for past exposure to cooking fuel or recent changes in cooking methods.  Authors categorized HHs using polluting fuels (kerosene, coal/lignite, charcoal, wood, straw/shrubs/grass, agricultural crop, and animal dung) under polluting fuel group, which is acceptable. |
| Bias due to deviations from intended exposures | Moderate |  | Two things imposed potential bias on this regard: first, the DHS captured information existing only at the time of the survey (cross-sectional); there could be fuel use/choice pattern shift among households. When households economically become stronger, they tend to shift to cleaner fuel. However, the observed rate of shift towards clean fuel is very slow |
| Bias due to missing data | Low |  | The study has response rate of 97.6% (for women). In addition, it is unlikely that missing information is vary between the two (HHs with polluting fuel and HHs with clean fuel) groups. |
| Bias in measurement of  the outcome | Moderate |  | The method used to ascertain the outcome was relatively good (**see step I**), but recall bias on date and months of events could be inevitable because of the nature of the data acquisition technique. Including all-cause mortality will also include mortality outcomes that were not associated with HAP. |
| Bias in selection of the  reported result | Low |  | All the variables considered in the analysis found in the dataset and the descriptive result can be found in the final report, so that it is less likely to commit selection of the reported result. |
| Overall bias | Critical |  | Most of confounding variables not controlled in the analysis |

1. RoB assessment for Stillbirth

**Step I: review question, potential confounders, co-interventions, and exposure and outcome measurement accuracy information**

| **Step I items** | **Response** |
| --- | --- |
| PECO question | Is there a difference in stillbirth risks between HHs using polluting fuels (kerosene, coal/lignite, charcoal, wood, straw/shrubs/grass, agricultural crop and animal dung) Vs HHs using clean fuels (electricity, liquid petroleum gas (LPG), natural gas and biogas, solar energy) for cooking? |
| Confounding for HAP and perinatal mortality | **Risk factors for Stillbirth**  **Maternal related**  Maternal age, infection status (HIV, syphilis, malaria and rubella), nutritional status (overweight/obesity), diabetes (re-existing or pregnancy induced), hypertensive disorders (pre-existing, pregnancy induced, preeclampsia, eclampsia), substance abuse (tobacco, alcohol and illicit drugs), violence against women  **Fetal factors**  Male sex, post-term pregnancy and Rhesus disease |
| Co-interventions | - None identified |
| Accuracy of the measurement of exposure to  HAP | The following techniques are placed in descending order of exposure assessment accuracy.   - Biological monitoring - Personal monitoring - Micro-environmental area-based monitoring - Questionnaire/self-report |
| Accuracy of the measurement of outcome (perinatal mortality) | - Vital registration systems, population censuses, household surveys, sample registration systems and demographic surveillance sites are methods used to measure child mortality. - Vital registration data is the preferred source of data for early neonatal mortality if the system is well functioning. Other methods suffer from sampling errors, under-reporting of deaths, and misreporting age at death are common issues. |

**Title of the study: Nisha et al. (2018):** Variations in perinatal mortality associated with different polluting fuel types and kitchen location in Bangladesh

**Step II:** hypothetical target experiment, including specific confounders and co-interventions from that study that will require consideration

1. target randomized trial specific to the study

| Design | Household randomized trial |
| --- | --- |
| Participants | Nationally representative 7 months and above singleton pregnancies and early neonates (ages of 0 and 6 days) sampled in Bangladesh DHS, which were conducted between 2004 to 2014. |
| Experimental intervention | polluting fuels (kerosene, coal/lignite, charcoal, wood, straw/shrubs/grass, agricultural crop and animal dung) for cooking |
| Comparator | clean fuels (electricity, liquid petroleum gas (LPG), natural gas and biogas) for cooking |

B1. Specify the outcome

Perinatal mortality

B2. If multiple outcomes presented, specify the numerical result being assessed

| **Confounding domains listed in step I** | | | |
| --- | --- | --- | --- |
| Confounding domain | Measured variable (s) | Is there evidence that  controlling for this  Variable was unnecessary? | Is the confounding domain measured  validly and reliably by this  Variable (or these variables)? |
| **Risk factors for Stillbirth**  **Maternal related:** Maternal age, infection status (HIV, syphilis, malaria and rubella), nutritional status (overweight/obesity), diabetes (re-existing or pregnancy induced), hypertensive disorders (pre-existing, pregnancy induced, preeclampsia, eclampsia), substance abuse (tobacco, alcohol and illicit drugs), violence against women  **Fetal factors:** Male sex, post-term pregnancy, Rhesus disease | None | No | No information |
| **Additional confounding domains relevant to the setting of this particular study, or which the study authors identified as important** | | | |
| maternal education, birth order, place of residence, maternal working status during survey, kitchen location and year of survey | None | No | Yes |

Step III: RoB across seven items

| **Rec. No. EndNote_74** | | | |
| --- | --- | --- | --- |
| **Bias items** | **Risk of**  **bias** | **Direction of bias** | **Rationale** |
| Bias due to confounding | Critical |  | Authors considered the following variables as a covariate: Maternal age at birth, maternal education, birth order, maternal body mass index (BMI), place of residence, wealth index, maternal working status during survey, kitchen location and year of survey. Some of these variables could be risk for still birth/early neonatal death. They keep these covariates identical for both outcomes. Myriads of variables that could affect both outcomes were not controlled. |
| Bias in selection of participants into the study | low |  | DHS implements probability sampling technique (selection of subjects was unrelated to either exposure or outcome status)  There is no means to commit bias in selection of participants. |
| Bias in classification of  exposures | Moderate |  | There may be misclassification in categorising participants into exposed to polluting fuel or not because some households could use a combination of polluting and clean fuels and information was only collected on primary fuel use in DHS survey.  This study also did not account for past exposure to cooking fuel or recent changes in cooking methods.  Authors categorized HHs using polluting fuels (kerosene, coal/lignite, charcoal, wood, straw/shrubs/grass, agricultural crop, and animal dung) under polluting fuel group, which is acceptable. |
| Bias due to deviations from intended exposures | Moderate |  | Two things imposed potential bias on this regard: first, the DHS captured information existing only at the time of the survey (cross-sectional); there could be fuel use/choice pattern shift among households. When households economically become stronger, they tend to shift to cleaner fuel. However, the observed rate of shift towards clean fuel is very slow |
| Bias due to missing data | Low |  | The study has response rate of 97.6% (for women). In addition, it is unlikely that missing information is vary between the two (HHs with polluting fuel and HHs with clean fuel) groups. |
| Bias in measurement of  the outcome | Moderate |  | The method used to ascertain the outcome was relatively good (**see step I**), but recall bias on date and months of events could be inevitable because of the nature of the data acquisition technique. Including all-cause mortality will also include mortality outcomes that were not associated with HAP. |
| Bias in selection of the  reported result | Low |  | All the variables considered in the analysis found in the dataset and the descriptive result can be found in the final report, so that it is less likely to commit selection of the reported result. |
| Overall bias | Critical |  | Most of confounding variables not controlled in the analysis |

**Title of the study: Khan et al. (2017):** Household air pollution from cooking and risk of adverse health and birth outcomes in Bangladesh: a nationwide population-based study

**Step II:** hypothetical target experiment, including specific confounders and co-interventions from that study that will require consideration

1. target randomized trial specific to the study

| Design | Household randomized trial |
| --- | --- |
| Participants | Nationally representative >=7 months pregnant women in Bangladesh included in BDHS collected in 2007, 2011 and 2014, within five years preceding the survey. |
| Experimental intervention | polluting fuels (kerosene, coal/lignite, charcoal, wood, straw/shrubs/grass, agricultural crop and animal dung) for cooking |
| Comparator | clean fuels (electricity, liquid petroleum gas (LPG), natural gas and biogas) for cooking |

B1. Specify the outcome

Stillbirth

B2. If multiple outcomes presented, specify the numerical result being assessed

| **Confounding domains listed in step I** | | | |
| --- | --- | --- | --- |
| Confounding domain | Measured variable (s) | Is there evidence that controlling for this variable was unnecessary? | Is the confounding domain measured validly and reliably by this Variable (or these variables)? |
| **Risk factors for Stillbirth**  **Maternal related**  Maternal age, infection status (HIV, syphilis, malaria and rubella), nutritional status (overweight/obesity), diabetes (re-existing or pregnancy induced), hypertensive disorders (pre-existing, pregnancy induced, preeclampsia, eclampsia), substance abuse (tobacco, alcohol and illicit drugs), violence against women  **Fetal factors**  Male sex, post-term pregnancy and Rhesus disease | None | No | No information |
| **Additional confounding domains relevant to the setting of this particular study, or which the study authors identified as important** | | | |
| education, place of residence, region, socio-economic status, breastfeeding and child sex | None | No | Yes |

Step III: RoB across seven items

| **Rec. No. EndNote_280** | | | |
| --- | --- | --- | --- |
| **Bias items** | **Risk of**  **bias** | **Direction of bias** | **Rationale** |
| Bias due to confounding | Critical |  | Authors controlled women’s age at birth, wealth quintiles, educational attainment, region of residence, place of residence and children’s gender. Most of the risk factors were not considered |
| Bias in selection of participants  into the study | low |  | DHS implements probability sampling technique (selection of subjects was unrelated to either exposure or outcome status)  There is no means to commit bias in selection of participants. |
| Bias in classification of  exposures | Serious |  | Authors classified cooking fuel in to two groups: solid and clean fuel. Even though kerosene is one of the fuel options in the category, authors have not mentioned what happened to kerosene; excluded or not.  In addition to this, some households could use a combination of solid and clean fuels, but this is not addressed in the survey. Furthermore, the study also did not account for past exposure to cooking fuel or recent changes in cooking methods. |
| Bias due to deviations from intended exposures | Moderate |  | The DHS is cross-sectional; there could be fuel use/choice pattern shift among households at some point in the past time. When households economically become stronger, they tend to shift to cleaner fuel. |
| Bias due to missing data | Low |  | The analysis is based on nationally representative sample, which 98% combined response rate. Yet again, missed information could be less likely to be specific to one category. |
| Bias in measurement of  the outcome | Moderate |  | The method used to ascertain the outcome was relatively good (**see step I**), but recall bias on date and months of events could be inevitable because of the nature of the data acquisition technique. Including all-cause mortality will also include mortality outcomes that were not associated with HAP. |
| Bias in selection of the  reported result | Low |  | All the variables considered in the analysis found in the dataset and the descriptive result can be found in the final report, so that it is less likely to commit bias in selection of the reported result. |
| Overall bias | Critical |  | Many risk factors of child mortality missed to be controlled and even though kerosene is found in the fuel type, it is not mentioned |

**Study title:** **Mishra et al. (2005):** Cooking smoke and tobacco smoke as risk factors for stillbirth

**Step II:** hypothetical target experiment, including specific confounders and co-interventions from that study that will require consideration

1. target randomized trial specific to the study

| Design | Household randomized trial |
| --- | --- |
| Participants | Nationally representative 40 – 49 years old Indian ever-married women with complete birth histories who were included in 1998-99 Indian’s NFHS. |
| Experimental intervention | polluting fuels (kerosene, coal/lignite, charcoal, wood, straw/shrubs/grass, agricultural crop and animal dung) for cooking |
| Comparator | clean fuels (electricity, liquid petroleum gas (LPG), natural gas and biogas) for cooking |

B1. Specify the outcome

Stillbirth

B2. If multiple outcomes presented, specify the numerical result being assessed

| **Confounding domains listed in step I** | | | |
| --- | --- | --- | --- |
| Confounding domain | Measured variable (s) | Is there evidence that controlling for this variable was unnecessary? | Is the confounding domain measured validly and reliably by this variable (or these variables)? |
| **Risk factors for Stillbirth**  **Maternal related**  Maternal age, infection status (HIV, syphilis, malaria and rubella), nutritional status (overweight/obesity), diabetes (re-existing or pregnancy induced), hypertensive disorders (pre-existing, pregnancy induced, preeclampsia, eclampsia), substance abuse (tobacco, alcohol and illicit drugs), violence against women  **Fetal factors**  Male sex, post-term pregnancy and Rhesus disease | BMI | No | No information |
| **Additional confounding domains relevant to the setting of this particular study, or which the study authors identified as important** | | | |
| Tobacco smoke, education, religion, ethnicity, house type, separate kitchen, crowding, standard of living, region & number of children | None | No | Yes |

Step III: RoB across seven items

| **Rec. No. EndNote_742** | | | |
| --- | --- | --- | --- |
| **Bias items** | **Risk of**  **bias** | **Direction of bias** | **Rationale** |
| Bias due to confounding | Critical |  | Authors considered the following variables as a confounder in their analysis: Tobacco smoke, anemia, BMI, education, religion, ethnicity, house type, separate kitchen, crowding, standard of living, region & number of children. However, some variables such as diabetes (re-existing or pregnancy induced), hypertensive disorders (pre-existing, pregnancy induced, preeclampsia, eclampsia), maternal age and presence of some infectious diseases were not considered. |
| Bias in selection of participants into the study | low |  | DHS implements probability sampling technique (selection of subjects was unrelated to either exposure or outcome status)  There is no means to commit bias in selection of participants. |
| Bias in classification of  exposures | Critical |  | Authors classified HHs using fuels such as electricity, liquid petroleum gas, biogas, or kerosene under cleaner fuel user category where they included the polluted fuel, kerosene. In addition, still there is a chance for some households to use a combination of polluting and clean fuels.  This study also did not account for past exposure to cooking fuel or recent changes in cooking methods. |
| Bias due to deviations from intended exposures | Serious |  | In addition to the mix of cleaner and polluted fuels made by authors, there could be shift in fuel use/choice pattern among households through time. When households economically become stronger, they tend to shift to cleaner fuel. |
| Bias due to missing data | Low |  | The study has response rate of 98.% for HH & 96% for women. In addition, it is unlikely that missing information is vary between the two (HHs with polluting fuel and HHs with clean fuel) groups. |
| Bias in measurement of  the outcome | Moderate |  | It is cross-sectional so that clear temporal associations between the exposure (cooking fuel) and outcome (mortality) cannot be established. |
| Bias in selection of the  reported result | Low |  | Authors analysed only a single outcome and reported it with all statistics mentioned in the method section. |
| Overall bias | Critical |  | Authors classified kerosene, which is polluting fuel under clean fuel category and many variables left uncontrolled. |

1. **RoB assessment: Nutritional Problem**

**Step I: review question, potential confounders, co-interventions, and exposure and outcome measurement accuracy information**

| **Step I items** | **Response** |
| --- | --- |
| PECO question | Is there a difference in nutritional defect (anaemia and stunting) risks between HHs using polluting fuels (kerosene, coal/lignite, charcoal, wood, straw/shrubs/grass, agricultural crop and animal dung) Vs HHs using clean fuels (electricity, liquid petroleum gas (LPG), natural gas and biogas, solar energy) for cooking? |
| Confounding for HAP and nutritional problem (anaemia & stunting) | **Risk factors for childhood malnutrition**  **Maternal nutrition and infection**  Maternal nutritional status (short stature, maternal underweight and maternal anemia status), maternal malaria, maternal age and short birth intervals  **Fetal growth restriction and preterm birth**  Preterm birth and Low birth weight  **Child nutrition and infection**  Childhood zinc deficiency, childhood diarrhea, breastfeeding status, exposed to infectious disease (HIV infection) and unimproved water & sanitation |
| Co-interventions | - None identified |
| Accuracy of the measurement of exposure to  HAP | The following techniques are placed in descending order of exposure assessment accuracy.   - Biological monitoring - Personal monitoring - Micro-environmental area-based monitoring - Questionnaire/self-report |
| Accuracy of the measurement of outcome (perinatal mortality) | - Anthropometric (measurement of the size, weight, and proportions of the body). Common anthropometric measurements include weight, height, MUAC, head circumference, and skinfold. Body mass index (BMI) and weight-for-height are anthropometric measurements presented as indexes) - Biochemical (means checking levels of nutrients in a person’s blood, urine, or stools) - Clinical (checking for visible signs of nutritional deficiencies such as bilateral pitting edema, emaciation (a sign of wasting, which is loss of muscle and fat tissue as a result of low energy intake and/or nutrient loss from infection), hair loss, and changes in hair color) - Dietary (Assessing food and fluid intake) |

**Title of the study:** **Dadras et al. (2017):** Biomass fuel smoke and stunting in early childhood: finding from a national survey in Nepal

**Step II:** hypothetical target experiment, including specific confounders and co-interventions from that study that will require consideration

1. target randomized trial specific to the study

| Design | Household randomized trial |
| --- | --- |
| Participants | Nationally representative Nepalese under-five children included in 2007 NDHS |
| Experimental intervention | HHs using polluting fuels (kerosene, coal/lignite, charcoal, wood, straw/shrubs/grass, agricultural crop and animal dung) |
| Comparator | HHs clean fuels (electricity, liquid petroleum gas (LPG), natural gas and biogas) |

B1. Specify the outcome

Stunting

B2. If multiple outcomes presented, specify the numerical result being assessed

| **Confounding domains listed in step I** | | | |
| --- | --- | --- | --- |
| Confounding domain | Measured variable (s) | Is there evidence that controlling for this Variable was unnecessary? | Is the confounding domain measured validly and reliably by this Variable (or these variables)? |
| **Risk factors for anaemia & stunting**  **Maternal nutrition and infection**  Maternal nutritional status, maternal malaria, maternal age, short birth intervals  **Fetal status**  Preterm birth and low birth weight  **Child nutrition and infection**  Childhood zinc deficiency, childhood diarrhea, breastfeeding status, exposed to infectious disease, unimproved water & sanitation | Maternal BMI | No | No information |
| **Additional confounding domains relevant to the setting of this particular study, or which the study authors identified as important** | | | |
| cooking lace, child age in months, birth order, mother's education, mother's smoking status, food security, region, ethnicity & wealth index | None | No | Yes |

Step III: RoB across seven items

| **Rec. No. EndNote_2724** | | | |
| --- | --- | --- | --- |
| **Bias items** | **Risk of**  **bias** | **Direction of bias** | **Rationale** |
| Bias due to confounding | Critical |  | Authors controlled cooking lace, child age in months, Birth size, birth order, mother's BMI, mother's education, mother's smoking status, availability of improved water source, food security, region, ethnicity & wealth index. Some child and maternal related confounders were not controlled. |
| Bias in selection of participants into the study | low |  | DHS implements probability sampling technique (selection of subjects was unrelated to either exposure or outcome status)  There is no means to commit bias in selection of participants. |
| Bias in classification of  exposures | Critical |  | As per the authors, HHs using LPG, natural gas, biogas, electricity, kerosene, coal or charcoal define the clean fuel users. They considered biomass fuels such as wood, dung, straw or agricultural crop users as exposed group. Many of the polluted fuel were categorised under clean fuel. Further exposure misclassification bias could be some households might use a combination of polluting and clean fuels and information was only collected on primary fuel use in the survey.  This study also did not account for past exposure to cooking fuel or recent changes in cooking methods. |
| Bias due to deviations from intended exposures | Moderate |  | Survey captured information present only at the time of data collection (cross-sectional); there could be fuel use/choice pattern shift among households. Different studies portrayed that when households economically become stronger, they tend to shift to cleaner fuel.  On the other way round, the progress in shift towards cleaner fuel is slow and small. |
| Bias due to missing data | Low |  | The study is based on nationally representative sample with 98% for women response rate. Furthermore, it is unlikely that missing information is vary between the two (HHs with polluting fuel and HHs with clean fuel) groups. |
| Bias in measurement of  the outcome | low |  | Weight was measured using a solar-powered digital scale with an accuracy of 100 g, and height was measured using an adjustable wooden measuring board to the nearest 0.1 cm. For maternal and child anaemia status, blood haemoglobin levels were measured using the portable HemoCue system. The haemoglobin measurements were adjusted for altitude. Then, all interpretation followed the WHO Child Growth Standards. Notwithstanding these efforts, it is cross-sectional so that clear temporal associations between the exposure and outcome cannot be established. |
| Bias in selection of the reported result | Low |  | Authors reported the outcomes they proposed in the method including the statistics mentioned. |
| Overall bias | Critical |  | Authors classified kerosene, coal and charcoal under comparator group (unexposed group). |

**Title of the study: Machisa et al. (2013):** Biomass fuel use for household cooking in Swaziland: is there an association with anaemia and stunting in children aged 6–36 months?

**Step II:** hypothetical target experiment, including specific confounders and co-interventions from that study that will require consideration

1. target randomized trial specific to the study

| Design | Household randomized trial |
| --- | --- |
| Participants | Nationally representative Swaziland children aged 6–36 months, which were conducted in 2006-7 SDHS |
| Experimental intervention | HHs using polluting fuels (kerosene, coal/lignite, charcoal, wood, straw/shrubs/grass, agricultural crop and animal dung) |
| Comparator | HHs clean fuels (electricity, liquid petroleum gas (LPG), natural gas and biogas) |

B1. Specify the outcome

Anaemia and stunting

B2. If multiple outcomes presented, specify the numerical result being assessed

| **Confounding domains listed in step I** | | | |
| --- | --- | --- | --- |
| Confounding domain | Measured variable (s) | Is there evidence that controlling for this Variable was unnecessary? | Is the confounding domain measured  validly and reliably by this  Variable (or these variables)? |
| **Risk factors for anaemia & stunting**  **Maternal nutrition and infection**  Maternal nutritional status, maternal malaria, maternal age, short birth intervals  **Fetal status**  Preterm birth and low birth weight  **Child nutrition and infection**  Childhood zinc deficiency, childhood diarrhea, breastfeeding status, exposed to infectious disease, unimproved water & sanitation | Maternal anaemia status | No | No information |
| **Additional confounding domains relevant to the setting of this particular study, or which the study authors identified as important** | | | |
| child related:  gender, age, birth order, ARI status, fever status  maternal related:  BMI, educational status, iron during pregnancy  household related:  urban/rural, region, crowding, wealth index | None | No | Yes |

Step III: RoB across seven items

| **Rec. No. EndNote_425** | | | |
| --- | --- | --- | --- |
| **Bias items** | **Risk of**  **bias** | **Direction of bias** | **Rationale** |
| Bias due to confounding | Low |  | Authors controlled the following child, maternal and HH related factors: Child related: gender, age, birth order, birth interval, birth weight, ARI status, fever status and diarrhea episodes. Maternal related: age at child birth, BMI, educational status, iron during pregnancy and anaemia status. Household related: urban/rural, region, crowding and wealth index. A few important confounders left to be controlled such as breast feeding status of the child and water and sanitation status of the household. |
| Bias in selection of participants into the study | low |  | DHS implements probability sampling technique (selection of subjects was unrelated to either exposure or outcome status)  There is no means to commit bias in selection of participants. |
| Bias in classification of  exposures | Serious | Towards the null | Authors categorised HHs using kerosene/paraffin with other cleaner fuels such as liquid petroleum gas/natural gas and electricity. In addition to this misclassification, there may be another misclassification in categorising participants into exposed to polluting fuel or not because some households could use a combination of polluting and clean fuels and information was only collected on primary fuel use in the survey.  This study also did not account for past exposure to cooking fuel or recent changes in cooking methods. |
| Bias due to deviations from intended exposures | Moderate |  | Survey captured information present only at the time of data collection (cross-sectional); there could be fuel use/choice pattern shift among households. different studies portrayed that when households economically become stronger, they tend to shift to cleaner fuel.  However, the trend to shift in to cleaner fuel such as clean gases and electricity is minimal and very slow. |
| Bias due to missing data | Low |  | The study is based on nationally representative sample, which usually is large adequate. Furthermore, it is unlikely that missing information is vary between the two (HHs with polluting fuel and HHs with clean fuel) groups. |
| Bias in measurement of  the outcome | Low |  | The anaemia status of children and their biological mothers was determined with the portable HemoCue system. Height measurements were conducted with a measuring board produced by Shorr Productions (Olney, MD, USA). Children aged, 24 months were measured while lying down (recumbent length) on the board and children aged ≥24 months while standing up then, the category was based on WHO classification for anaemia & stunting.  Notwithstanding these efforts, it is cross-sectional so that clear temporal associations between the exposure and outcome cannot be established. |
| Bias in selection of the  reported result | Low |  | Authors reported all what is planned in the method, including the statistics mentioned. |
| Overall bias | Serious |  | Authors classified kerosene/paraffin, which is polluting fuel under clean fuel category. However, the study is based on nationally representative data and the classification affects the outcome towards the null. |

**Title of the study:** **Kyu et al. (2010):** Biofuel Smoke and Child Anemia in 29 Developing Countries: A Multilevel Analysis

**Step II:** hypothetical target experiment, including specific confounders and co-interventions from that study that will require consideration

1. target randomized trial specific to the study

| Design | Household randomized trial |
| --- | --- |
| Participants | Nationally representative children (0-59 months) from 29 developing countries included in the DHS conducted between 2003 and 2007 |
| Experimental intervention | HHs using polluting fuels (kerosene, coal/lignite, charcoal, wood, straw/shrubs/grass, agricultural crop and animal dung), exclusively |
| Comparator | HHs clean fuels (electricity, liquid petroleum gas (LPG), natural gas and biogas), exclusively |

B1. Specify the outcome

Anemia

B2. If multiple outcomes presented, specify the numerical result being assessed

| **Confounding domains listed in step I** | | | |
| --- | --- | --- | --- |
| Confounding domain | Measured variable (s) | Is there evidence that controlling for this Variable was unnecessary? | Is the confounding domain measured validly and reliably by this Variable (or these variables)? |
| **Risk factors for anaemia & stunting**  **Maternal nutrition and infection**  **Maternal nutrition and infection**  Maternal nutritional status, maternal malaria, maternal age, short birth intervals  **Fetal status**  Preterm birth and low birth weight  **Child nutrition and infection**  Childhood zinc deficiency, childhood diarrhea, breastfeeding status, exposed to infectious disease, unimproved water & sanitation | Maternal haemoglobin (Hg (g/dl) | No | No information |
| **Additional confounding domains relevant to the setting of this particular study, or which the study authors identified as important** | | | |
| maternal related (education, smoking status and iron during pregnancy)  child related (age, sex, wealth index, total children, country and cluster | None | No | Yes |

Step III: RoB across seven items

| **Rec. No. EndNote_2740** | | | |
| --- | --- | --- | --- |
| **Bias items** | **Risk of**  **bias** | **Direction of bias** | **Rationale** |
| Bias due to confounding | Moderate |  | Authors controlled maternal related factors such as age, education, smoking status, Hg (g/dl) and iron during pregnancy), child related (age, sex, height-for-age, weight-for-height, diarrhea & fever status) and other variables such as wealth index, total children, country and cluster. Child’s breast-feeding status, weight at birth and birth intervals were few of the confounders left to be controlled. |
| Bias in selection of participants into the study | low |  | DHS implements probability sampling technique (selection of subjects was unrelated to either exposure or outcome status)  There is no means to commit bias in selection of participants. |
| Bias in classification of  exposures | Serious | Towards the null | As per the authors considered HHs using Electricity, natural gas, biogas, and kerosene as unexposed group. Kerosene is type of polluted fuel, so that including it in the analysis with other clean fuel is an introduction of exposure misclassification bias. Similarly, exposure misclassification bias could be some households might use a combination of polluting and clean fuels and information was only collected on primary fuel use in the survey.  This study also did not account for past exposure to cooking fuel or recent changes in cooking methods. |
| Bias due to deviations from intended exposures | Moderate |  | Survey captured information present only at the time of data collection (cross-sectional); there could be fuel use/choice pattern shift among households. Different studies portrayed that when households economically become stronger, they tend to shift to cleaner fuel.  On the other way round, the progress in shift towards cleaner fuel is slow and small, which can be seen as lower chance of households to shift from one form of fuel to another (cleaner). |
| Bias due to missing data | Low |  | The study is based on nationally representative sample with >90% for women response rate. Furthermore, it is unlikely that missing information is vary between the two (HHs with polluting fuel and HHs with clean fuel) groups. |
| Bias in measurement of  the outcome | Low |  | Trained data collectors used HemoCue machines to measure the hemoglobin levels of women and children and the hemoglobin levels were adjusted by altitude. The measured value was categorised as no anemia (Hb >= 11.0 g/dL), mild anemia (Hb between 10.0 g/dL and 10.9 g/dL), and moderate/severe anemia (Hb<10 g/dL). Authors combined moderate anemia and severe anemia into one category.  However, it is cross-sectional so that clear temporal associations between the exposure and outcome cannot be established. |
| Bias in selection of the reported result | Low |  | Authors reported the outcomes they proposed in the method including the statistics mentioned. |
| Overall bias | Serious |  | Authors classified kerosene under comparator group (unexposed group). The classification of exposure variable affects the outcome towards the null and the large sample size could reduce risk of bias, in general. |

**Title of the study:** **Kyu et al. (2009):** Maternal smoking, biofuel smoke exposure and child height-for-age in seven developing countries

**Step II:** hypothetical target experiment, including specific confounders and co-interventions from that study that will require consideration

1. target randomized trial specific to the study

| Design | Household randomized trial |
| --- | --- |
| Participants | Nationally representative children (0–59 months) from seven resource poor countries included in respective country’s DHS, which was conducted between 2005 and 2007 |
| Experimental intervention | HHs using polluting fuels (kerosene, coal/lignite, charcoal, wood, straw/shrubs/grass, agricultural crop and animal dung) |
| Comparator | HHs clean fuels (electricity, liquid petroleum gas (LPG), natural gas and biogas) |

B1. Specify the outcome

Height-for-age

B2. If multiple outcomes presented, specify the numerical result being assessed

| **Confounding domains listed in step I** | | | |
| --- | --- | --- | --- |
| Confounding domain | Measured variable (s) | Is there evidence that  controlling for this Variable was unnecessary? | Is the confounding domain measured  validly and reliably by this Variable (or these variables)? |
| **Risk factors for anaemia & stunting**  **Maternal nutrition and infection**  Maternal nutritional status, maternal malaria, maternal age, short birth intervals  **Fetal status**  Preterm birth and low birth weight  **Child nutrition and infection**  Childhood zinc deficiency, childhood diarrhea, breastfeeding status, exposed to infectious disease, unimproved water & sanitation | None | No | No information |
| **Additional confounding domains relevant to the setting of this particular study, or which the study authors identified as important** | | | |
| Maternal smoking, Men smoking, gender & age of the child, mother’s education, number of total children, Wealth index and country of data | None | No | Yes |

Step III: RoB across seven items

| **Rec. No. EndNote_1765** | | | |
| --- | --- | --- | --- |
| **Bias items** | **Risk of**  **bias** | **Direction of bias** | **Rationale** |
| Bias due to confounding | Serious |  | Authors controlled mother’s & father’s smoking status, gender & age of the child, birth size, early breastfeeding, mother’s age & education, number of total children, wealth index and country of data. Some of these variables could be determining factors in specific place, but some known determinant variables were not controlled. |
| Bias in selection of participants into the study | low |  | DHS implements probability sampling technique (selection of subjects was unrelated to either exposure or outcome status)  There is no means to commit bias in selection of participants. |
| Bias in classification of  exposures | Serious | Towards the null | Authors categorised HHs using kerosene with other cleaner fuels such as liquid petroleum gas/natural gas and electricity and considered as comparator group. Another potential source of misclassification bias could be some households might use a combination of polluting and clean fuels and information was only collected on primary fuel use in the survey.  This study also did not account for past exposure to cooking fuel or recent changes in cooking methods. |
| Bias due to deviations from intended exposures | Moderate |  | Survey captured information present only at the time of data collection (cross-sectional); there could be fuel use/choice pattern shift among households. different studies portrayed that when households economically become stronger, they tend to shift to cleaner fuel. Regardless of these realities, the progress towards cleaner fuel shift is slow and minimal. |
| Bias due to missing data | Low |  | The study is based on nationally representative sample with >90% response rate. Furthermore, it is unlikely that missing information is vary between the two (HHs with polluting fuel and HHs with clean fuel) groups. |
| Bias in measurement of  the outcome | Low |  | Standardized height measuring board were used to measure the height of children (0–59 months). Children younger than 24 months were measured lying down on the board, whereas standing height was measured for older children. The height-for-age measures interpreted according to WHO Child Growth Standards. Notwithstanding these efforts, it is cross-sectional so that clear temporal associations between the exposure and outcome cannot be established. |
| Bias in selection of the  reported result | Low |  | Authors reported all what is planned in the method, including the statistics mentioned. |
| Overall bias | Serious |  | Authors classified kerosene under comparator group, while it is polluting fuel. However,, this classification tends the result toward the null. On the other hand, the analysis is based on large sample size, which is one way to minimize bias. |

**Title of the study:** **Mishra et al. (2007):** Does biofuel smoke contribute to anaemia and stunting in early childhood?

**Step II:** hypothetical target experiment, including specific confounders and co-interventions from that study that will require consideration

1. target randomized trial specific to the study

| Design | Household randomized trial |
| --- | --- |
| Participants | Nationally representative Indian children (aged 0–35 months) that were included in Indian 1998–99 NFHS. |
| Experimental intervention | HHs using polluting fuels (kerosene, coal/lignite, charcoal, wood, straw/shrubs/grass, agricultural crop and animal dung) |
| Comparator | HHs clean fuels (electricity, liquid petroleum gas (LPG), natural gas and biogas) |

B1. Specify the outcome

Anaemia & stunting

B2. If multiple outcomes presented, specify the numerical result being assessed

| **Confounding domains listed in step I** | | | |
| --- | --- | --- | --- |
| Confounding domain | Measured variable (s) | Is there evidence that controlling for this Variable was unnecessary? | Is the confounding domain measured validly and reliably by this Variable (or these variables)? |
| **Risk factors for anaemia & stunting**  **Maternal nutrition and infection**  Maternal nutritional status, maternal malaria, maternal age, short birth intervals  **Fetal status**  Preterm birth and low birth weight  **Child nutrition and infection**  Childhood zinc deficiency, childhood diarrhea, breastfeeding status, exposed to infectious disease, unimproved water & sanitation | Maternal BMI & anaemia status | No | No information |
| **Additional confounding domains relevant to the setting of this particular study, or which the study authors identified as important** | | | |
| Iron during pregnancy, ARI in past 2 weeks, malaria in past 3 months, Mother’s education, Age of child, Sex of child, birth order, religion, ethnicity, house type, residence, crowding, living standard, region & Environmental tobacco smoke | None | No | Yes |

Step III: RoB across seven items

| **Rec. No. EndNote_1904** | | | |
| --- | --- | --- | --- |
| **Bias items** | **Risk of**  **bias** | **Direction of bias** | **Rationale** |
| Bias due to confounding | Moderate |  | Authors controlled maternal factors (Iron during pregnancy, Mother’s age at childbirth, Mother’s BMI, Mother’s anaemia status, Mother’s education, religion, ethnicity), child factors (ARI in past 2 weeks, diarrhoea in past 2 weeks, malaria in past 3 months, Age of child, Sex of child, birth order) & environmental factors (house type, residence, crowding, living standard, region & Environmental tobacco smoke). |
| Bias in selection of participants into the study | low |  | DHS implements probability sampling technique (selection of subjects was unrelated to either exposure or outcome status)  There is no means to commit bias in selection of participants. |
| Bias in classification of  exposures | Serious | Towards the null | Authors categorised HHs using kerosene with other cleaner fuels such as liquid petroleum gas/natural gas and electricity and considered as comparator group. The other two groups formed by the authors are highly exposed-HHs using only biofuels (wood, crop residues, or dung cakes) and medium exposed- HHs using a mix of biofuels and cleaner fuels or coal/coke/lignite/charcoal. Here the problem is, kerosene, which is polluted fuel is categorized under clean fuel. Another potential source of misclassification bias could be some households might use a combination of polluting and clean fuels and information was only collected on primary fuel use in the survey.  This study also did not account for past exposure to cooking fuel or recent changes in cooking methods. |
| Bias due to deviations from intended exposures | Moderate |  | Survey captured information present only at the time of data collection (cross-sectional); there could be fuel use/choice pattern shift among households. different studies portrayed that when households economically become stronger, they tend to shift to cleaner fuel.  On the other way round, the progress in shift towards cleaner fuel is slow and small. |
| Bias due to missing data | Low |  | The study is based on nationally representative sample with 98% for HHs and 96% for women response rate. Furthermore, it is unlikely that missing information is vary between the two (HHs with polluting fuel and HHs with clean fuel) groups. |
| Bias in measurement of  the outcome | Low |  | Weight was measured using a solar-powered digital scale with an accuracy of 100 g, and height was measured using an adjustable wooden measuring board to the nearest 0.1 cm. For maternal and child anaemia status, blood haemoglobin levels were measured using the portable HemoCue system. The haemoglobin measurements were adjusted for altitude. Then, all interpretation followed the WHO Child Growth Standards. Notwithstanding these efforts, it is cross-sectional so that clear temporal associations between the exposure and outcome cannot be established. |
| Bias in selection of the reported result | Low |  | Authors reported the outcomes they proposed in the method including the statistics mentioned. |
| Overall bias | Serious |  | Authors classified kerosene under comparator group, while it is polluting fuel. However, the classification affects the result towards the null |

1. **RoB assessment for Birth weight**

**Step I: review question, potential confounders, co-interventions, and exposure and outcome measurement accuracy information**

| **Step I items** | **Response** |
| --- | --- |
| PECO question | Is there a difference in reduced birth weight risks between HHs using polluting fuels (kerosene, coal/lignite, charcoal, wood, straw/shrubs/grass, agricultural crop and animal dung) Vs HHs using clean fuels (electricity, liquid petroleum gas (LPG), natural gas and biogas, solar energy) for cooking? |
| Confounding for HAP and nutritional problem (birth weight) | **Risk factors for low birth weight**  Preterm birth, mother's age (teen-age mothers), multiple gestation/birth, narrow child spacing, substance use (illicit drugs, tobacco and alcohol), mothers of lower socioeconomic status, poorer pregnancy nutrition and low body mass index, infectious diseases (including HIV and malaria), emotional and physical stress, low maternal education, inadequate prenatal care and pregnancy complications |
| Co-interventions | - None identified |
| Accuracy of the measurement of exposure to  HAP | The following techniques are placed in descending order of exposure assessment accuracy.   - Biological monitoring - Personal monitoring - Micro-environmental area-based monitoring - Questionnaire/self-report |
| Accuracy of the measurement of outcome (weight at birth) | - Newborn infant weighed within 24 h of birth using electronic/digital weight scale - Newborn infant weighed within 24 h of birth using Scale (electronic/spring) - Using proxy measure of birth weight (maternal assessments of infant birth size). It is poor measure of birth weight. High chance of over or under estimation. |

**Title of the study: Milanzi et al. (2017):** Maternal biomass smoke exposure and birth weight in Malawi: Analysis of data from the 2010 Malawi Demographic and Health Survey

**Step II:** hypothetical target experiment, including specific confounders and co-interventions from that study that will require consideration

1. target randomized trial specific to the study

| Design | Household randomized trial |
| --- | --- |
| Participants | Nationally representative under-five Malawi children included in Malawi DHS which were conducted during 2010 (2010 MDHS) |
| Experimental intervention | HHs using polluting fuels (kerosene, coal/lignite, charcoal, wood, straw/shrubs/grass, agricultural crop and animal dung) |
| Comparator | HHs clean fuels (electricity, liquid petroleum gas (LPG), natural gas and biogas) |

B1. Specify the outcome

Birth weight

B2. If multiple outcomes presented, specify the numerical result being assessed

| **Confounding domains listed in step I** | | | |
| --- | --- | --- | --- |
| Confounding domain | Measured variable (s) | Is there evidence that controlling for this Variable was unnecessary? | Is the confounding domain measured  validly and reliably by this Variable (or these variables)? |
| - Preterm birth, mother's age (teen-age mothers), multiple gestation/birth, narrow child spacing, substance use (illicit drugs, tobacco and alcohol), mothers of lower socioeconomic status, poorer pregnancy nutrition and low body mass index, infectious diseases (including HIV and malaria), emotional and physical stress, low maternal education, inadequate prenatal care and pregnancy complications | Maternal BMI | No | No information |
| **Additional confounding domains relevant to the setting of this particular study, or which the study authors identified as important** | | | |
| gender and birth order of the child, maternal religion and place of residence | None | No | Yes |

Step III: RoB across seven items

| **Rec. No. EndNote_2723** | | | |
| --- | --- | --- | --- |
| **Bias items** | **Risk of**  **bias** | **Direction of bias** | **Rationale** |
| Bias due to confounding | Low |  | Authors controlled gender and birth order of the child, maternal age at delivery, maternal education, maternal body mass index (BMI), maternal religion, wealth index and place of residence. In addition to this, they excluded multiple births, (twins) children from their analysis. |
| Bias in selection of participants into the study | low |  | DHS implements probability sampling technique (selection of subjects was unrelated to either exposure or outcome status)  There is no means to commit bias in selection of participants. |
| Bias in classification of  exposures | Serious |  | Authors used HHs using charcoal, wood, crops, straw and dung as exposed group and HHs using electricity, LPG and biogas as comparator group. Even though Kerosene was one of the choices in the fuel type, authors did not say anything what measure they took on it. Again, there may be another misclassification in categorising participants into exposed to polluting fuel or not because some households could use a combination of polluting and clean fuels and information was only collected on primary fuel use in the survey.  This study also did not account for past exposure to cooking fuel or recent changes in cooking methods. |
| Bias due to deviations from intended exposures | Moderate |  | Survey captured information present only at the time of data collection (cross-sectional); there could be fuel use/choice pattern shift among households. Different studies portrayed that when households economically become stronger; they tend to shift to cleaner fuel.  However, the trend to shift in to cleaner fuel such as clean gases and electricity is minimal and very slow. |
| Bias due to missing data | Low |  | The study is based on nationally representative sample, which usually is large adequate. Furthermore, it is unlikely that missing information is vary between the two (HHs with polluting fuel and HHs with clean fuel) groups. |
| Bias in measurement of  the outcome | Serious |  | Birth weight is recorded in two ways during survey: one from card and the other from maternal recall. Additionally, mothers interrogated to judge the size of their babies at birth. This might imposes poor quality of birth weight determination. Furthermore, it is cross-sectional so that clear temporal associations between the exposure and outcome cannot be established. |
| Bias in selection of the  reported result | Low |  | Authors reported all what was planned in the method, including the statistics mentioned. |
| Overall bias | Serious |  | Maternal recall of her under-five child weight at birth induced bias in determining exact weight at birth. Additionally, authors did not describe the fate of kerosene; in which group it was considered. |

**Title of the study: Khan et al. (2017):** Household air pollution from cooking and risk of adverse health and birth outcomes in Bangladesh: a nationwide population based study

**Step II:** hypothetical target experiment, including specific confounders and co-interventions from that study that will require consideration

1. target randomized trial specific to the study

| Design | Household randomized trial |
| --- | --- |
| Participants | Nationally representative Bangladesh under-five children included in BDHS collected in 2007, 2011 and 2014, live-born children within five years preceding the survey; only outcomes of the most recent births were considered |
| Experimental intervention | polluting fuels (kerosene, coal/lignite, charcoal, wood, straw/shrubs/grass, agricultural crop and animal dung) for cooking |
| Comparator | clean fuels (electricity, liquid petroleum gas (LPG), natural gas and biogas) for cooking |

B1. Specify the outcome

Birth weight

B2. If multiple outcomes presented, specify the numerical result being assessed

| **Confounding domains listed in step I** | | | |
| --- | --- | --- | --- |
| Confounding domain | Measured variable (s) | Is there evidence that  controlling for this Variable was unnecessary? | Is the confounding domain measured validly and reliably by this Variable (or these variables)? |
| Preterm birth, mother's age (teen-age mothers), multiple gestation/birth, narrow child spacing, substance use (illicit drugs, tobacco and alcohol), mothers of lower socioeconomic status, poorer pregnancy nutrition and low body mass index, infectious diseases (including HIV and malaria), emotional and physical stress, low maternal education, inadequate prenatal care and pregnancy complications | None | No | No information |
| **Additional confounding domains relevant to the setting of this particular study, or which the study authors identified as important** | | | |
| region of residence, place of residence and children’s gender | None | No | Yes |

Step III: RoB across seven items

| **Rec. No. EndNote_280** | | | |
| --- | --- | --- | --- |
| **Bias items** | **Risk of**  **bias** | **Direction of bias** | **Rationale** |
| Bias due to confounding | Serious |  | Authors controlled women’s age at birth, wealth quintiles, educational attainment, region of residence, place of residence and children’s gender. |
| Bias in selection of participants  into the study | low |  | DHS implements probability sampling technique (selection of subjects was unrelated to either exposure or outcome status)  There is no means to commit bias in selection of participants. |
| Bias in classification of  exposures | Serious |  | Authors classified cooking fuel in to two groups: solid and clean fuel. Even though kerosene is one of the fuel options in the category, authors have not mentioned what happened to kerosene; excluded or not.  In addition to this, some households could use a combination of solid and clean fuels, but this is not addressed in the survey. Furthermore, the study also did not account for past exposure to cooking fuel or recent changes in cooking methods. |
| Bias due to deviations from intended exposures | Moderate |  | The DHS is cross-sectional; there could be fuel use/choice pattern shift among households at some point in the past time. When households economically become stronger, they tend to shift to cleaner fuel. |
| Bias due to missing data | Low |  | The analysis is based on nationally representative sample, which 98% combined response rate. Yet again, missed information could be less likely to be specific to one category. |
| Bias in measurement of  the outcome | Serious |  | Authors sought information only on the most recent birth, which could serve as a recall bias reduction mechanism. Especially, when the vital registration of the countries is weak, it is the preferred method. However, authors did not elaborate source of birth weight data; either from health card or from maternal recall. |
| Bias in selection of the  reported result | Low |  | All the variables considered in the analysis found in the dataset and the descriptive result can be found in the final report, so that it is less likely to commit bias in selection of the reported result. |
| Overall bias | Serious |  | Many risk factors of child mortality missed to be controlled and even though kerosene is found in the fuel type, it is not mentioned |

**Title of the study: Epstein et al. (2013):** Household fuels, low birth weight, and neonatal death in India: The separate impacts of biomass, kerosene, and coal

**Step II:** hypothetical target experiment, including specific confounders and co-interventions from that study that will require consideration

1. target randomized trial specific to the study

| Design | Household randomized trial |
| --- | --- |
| Participants | Nationally representative Indian mother-infant pair of singleton birth included in Indian National Family Health Survey conducted in 2005-6 NFHS. |
| Experimental intervention | HHs using polluting fuels (kerosene, coal/lignite, charcoal, wood, straw/shrubs/grass, agricultural crop and animal dung) |
| Comparator | HHs clean fuels (electricity, liquid petroleum gas (LPG), natural gas and biogas) |

B1. Specify the outcome

Birth weight

B2. If multiple outcomes presented, specify the numerical result being assessed

| **Confounding domains listed in step I** | | | |
| --- | --- | --- | --- |
| Confounding domain | Measured variable (s) | Is there evidence that controlling for this Variable was unnecessary? | Is the confounding domain measured validly and reliably by this Variable (or these variables)? |
| Preterm birth, mother's age (teen-age mothers), multiple gestation/birth, narrow child spacing, substance use (illicit drugs, tobacco and alcohol), mothers of lower socioeconomic status, poorer pregnancy nutrition and low body mass index, infectious diseases (including HIV and malaria), emotional and physical stress, low maternal education, inadequate prenatal care and pregnancy complications | Maternal BMI & and anaemia | No | No information |
| **Additional confounding domains relevant to the setting of this particular study, or which the study authors identified as important** | | | |
| Infant gender, mother’s religion, occupation, Region, residence, HH access to electricity, Type of house, HH water source, mother’s perception for medical care and parity | None | No | Yes |

Step III: RoB across seven items

| **Rec. No. EndNote_429** | | | |
| --- | --- | --- | --- |
| **Bias items** | **Risk of**  **bias** | **Direction of bias** | **Rationale** |
| Bias due to confounding | Low |  | Authors controlled Infant gender, mother's age, education, religion, smoking status, occupation, BMI, region, residence, HH access to electricity, type of house, HH water source, mother’s perception for medical care, No. of ANC, parity and prior inter-birth interval. Additionally, they considered only singleton birth. |
| Bias in selection of participants into the study | low |  | DHS implements probability sampling technique (selection of subjects was unrelated to either exposure or outcome status)  There is no means to commit bias in selection of participants. |
| Bias in classification of  exposures | Moderate |  | Authors analysed the effect of coal, kerosene, charcoal and biomass fuel on birth weight, independently. They excluded electricity from comparator; used only LPG, biogas and natural gas. However, there may be misclassification in categorising participants into exposed to polluting fuel or not because some households could use a combination of polluting and clean fuels and information was only collected on primary fuel use in the survey.  This study also did not account for past exposure to cooking fuel or recent changes in cooking methods. |
| Bias due to deviations from intended exposures | Moderate |  | Survey captured information present only at the time of data collection (cross-sectional); there could be fuel use/choice pattern shift among households. Different studies portrayed that when households economically become stronger; they tend to shift to cleaner fuel.  However, the trend to shift in to cleaner fuel such as clean gases and electricity is minimal and very slow. |
| Bias due to missing data | Low |  | The study is based on nationally representative sample with 94.5% response rate. Furthermore, it is unlikely that missing information is vary between the two (HHs with polluting fuel and HHs with clean fuel) groups. |
| Bias in measurement of  the outcome | Moderate |  | Birth weight was obtained from card. The quality of the record is depend on the trends in health facilities (weight scale used and appropriate recording). In addition, it might not properly kept at home, which could lead to poor reading during survey). Furthermore, it is cross-sectional so that clear temporal associations between the exposure and outcome cannot be established. |
| Bias in selection of the  reported result | Low |  | Authors reported all what was planned in the method, including the statistics mentioned. |
| Overall bias | Moderate |  | Authors controlled most of risk factors for weight at birth and analysed the independent effect of polluting fuels. Additionally, they only considered infants with birth weight on card; they did not consider maternal recall. |

**Title of the study: Sreeramareddy et al. (2011):** Association between biomass fuel use and maternal report of child size at birth - an analysis of 2005-06 India Demographic Health Survey data

**Step II:** hypothetical target experiment, including specific confounders and co-interventions from that study that will require consideration

1. target randomized trial specific to the study

| Design | Household randomized trial |
| --- | --- |
| Participants | Nationally representative Indian mother-infant pair of singleton birth included in Indian National Family Health Survey conducted in 2005-6 NFHS. |
| Experimental intervention | HHs using polluting fuels (kerosene, coal/lignite, charcoal, wood, straw/shrubs/grass, agricultural crop and animal dung) |
| Comparator | HHs clean fuels (electricity, liquid petroleum gas (LPG), natural gas and biogas) |

B1. Specify the outcome

Child size at birth (from maternal report)

B2. If multiple outcomes presented, specify the numerical result being assessed

| **Confounding domains listed in step I** | | | |
| --- | --- | --- | --- |
| Confounding domain | Measured variable (s) | Is there evidence that controlling for this Variable was unnecessary? | Is the confounding domain measured validly and reliably by this Variable (or these variables)? |
| Preterm birth, mother's age (teen-age mothers), multiple gestation/birth, narrow child spacing, substance use (illicit drugs, tobacco and alcohol), mothers of lower socioeconomic status, poorer pregnancy nutrition and low body mass index, infectious diseases (including HIV and malaria), emotional and physical stress, low maternal education, inadequate prenatal care and pregnancy complications | Maternal BMI & anaemia | No | No information |
| **Additional confounding domains relevant to the setting of this particular study, or which the study authors identified as important** | | | |
| Child’s gender, and birth order, mother’s religion, residence (urban/rural) | None | No | Yes |

Step III: RoB across seven items

| **Rec. No. EndNote_2569** | | | |
| --- | --- | --- | --- |
| **Bias items** | **Risk of**  **bias** | **Direction of bias** | **Rationale** |
| Bias due to confounding | Low |  | Authors controlled child factors (gender, and birth order), maternal factors (anaemia, BMI, age at childbirth, smoking, education) and socio-demographic factors (religion, wealth index, residence urban/rural). Additionally, they excluded multiple birth and births with missing information about size at birth. Weight at birth, gestational age, pregnancy type, complications during pregnancy, mother’s condition of infection from diseases like HIV and ANC follow up status were a few variables not controlled. |
| Bias in selection of participants into the study | low |  | DHS implements probability sampling technique (selection of subjects was unrelated to either exposure or outcome status)  There is no means to commit bias in selection of participants. |
| Bias in classification of  exposures | Moderate |  | HHs using high pollution fuels (wood, straw, animal dung, and crop residues, kerosene, coal and charcoal) were considered as exposed group whereas HHs using low pollution fuels (electricity, liquid petroleum gas (LPG), natural gas and biogas) grouped as comparator. However, there may be misclassification in categorising participants into exposed to polluting fuel or not because some households could use a combination of polluting and clean fuels and information was only collected on primary fuel use in the survey.  This study also did not account for past exposure to cooking fuel or recent changes in cooking methods. |
| Bias due to deviations from intended exposures | Moderate |  | Survey captured information present only at the time of data collection (cross-sectional); there could be fuel use/choice pattern shift among households. Different studies portrayed that when households economically become stronger; they tend to shift to cleaner fuel.  However, the trend to shift in to cleaner fuel such as clean gases and electricity is minimal and very slow. |
| Bias due to missing data | Low |  | The study is based on nationally representative sample with 94.5% response rate. Furthermore, it is unlikely that missing information is vary between the two (HHs with polluting fuel and HHs with clean fuel) groups. |
| Bias in measurement of  the outcome | Serious |  | Birth weight was obtained from three sources: card, maternal recall and judgement from mothers. Authors stated that about 60% of the births had weight at birth neither from card nor from mother’s recall. Therefore, mother’s judgement about size at birth was used as a proxy for birth weight to classify children as low birth weight and normal birth weight. This potentially led to bias. Furthermore, it is cross-sectional so that clear temporal associations between the exposure and outcome cannot be established. |
| Bias in selection of the  reported result | Low |  | Authors reported all what was planned in the method, including the statistics mentioned. |
| Overall bias | Serious |  | Outcome measurement method and a few variables missed to be controlled could lead to bias. |

**Title of the study: Mishra et al. (2004):** Maternal Exposure to Biomass Smoke and Reduced Birth Weight in Zimbabwe

**Step II:** hypothetical target experiment, including specific confounders and co-interventions from that study that will require consideration

1. target randomized trial specific to the study

| Design | Household randomized trial |
| --- | --- |
| Participants | Nationally representative singleton births in Zimbabwe included in Zimbabwe Demographic and Health Survey conducted in 1999 ZDHS. |
| Experimental intervention | HHs using polluting fuels (kerosene, coal/lignite, charcoal, wood, straw/shrubs/grass, agricultural crop and animal dung) |
| Comparator | HHs clean fuels (electricity, liquid petroleum gas (LPG), natural gas and biogas) |

B1. Specify the outcome

Birth weight from card & from mother’s recall

B2. If multiple outcomes presented, specify the numerical result being assessed

| **Confounding domains listed in step I** | | | |
| --- | --- | --- | --- |
| Confounding domain | Measured variable (s) | Is there evidence that controlling for this Variable was unnecessary? | Is the confounding domain measured validly and reliably by this Variable (or these variables)? |
| Preterm birth, mother's age (teen-age mothers), multiple gestation/birth, narrow child spacing, substance use (illicit drugs, tobacco and alcohol), mothers of lower socioeconomic status, poorer pregnancy nutrition and low body mass index, infectious diseases (including HIV and malaria), emotional and physical stress, low maternal education, inadequate prenatal care and pregnancy complications | Maternal BMI | No | No information |
| **Additional confounding domains relevant to the setting of this particular study, or which the study authors identified as important** | | | |
| Sex of child, birth order of child, Iron supplement during pregnancy, religion of mother & region | None | No | Yes |

Step III: RoB across seven items

| **Rec. No. EndNote_2661** | | | |
| --- | --- | --- | --- |
| **Bias items** | **Risk of**  **bias** | **Direction of bias** | **Rationale** |
| Bias due to confounding | Moderate |  | Sex of child, Birth order of child, Mother’s age at child birth, Body mass index (BMI), Iron supplement during pregnancy, Malaria drug during pregnancy, Education of mother, Religion of mother, Standard of living index & region were variables considered as a confounders. There are other variables missed to be controlled. |
| Bias in selection of participants into the study | low |  | DHS implements probability sampling technique (selection of subjects was unrelated to either exposure or outcome status)  There is no means to commit bias in selection of participants. |
| Bias in classification of  exposures | Moderate |  | Authors classified exposed group in to two: HHs using  1. high pollution fuels (wood, dung, or straw) &  2. Medium pollution fuels (kerosene or charcoal). However, they only present the descriptive result of the second category. This could resulted the effect of polluting cooking fuel towards the null. There might me a case in which households could use a combination of polluting and clean fuels and information was only collected on primary fuel use in the survey. This study also did not account for past exposure to cooking fuel or recent changes in cooking methods. |
| Bias due to deviations from intended exposures | Moderate |  | Survey captured information present only at the time of data collection (cross-sectional); there could be fuel use/choice pattern shift among households. Different studies portrayed that when households economically become stronger; they tend to shift to cleaner fuel.  However, the trend to shift in to cleaner fuel such as clean gases and electricity is minimal and very slow. |
| Bias due to missing data | Low |  | The study is based on nationally representative sample with 97.8% response rate. Furthermore, it is unlikely that missing information is vary between the two (HHs with polluting fuel and HHs with clean fuel) groups. |
| Bias in measurement of  the outcome | Serious |  | Birth weight is obtained from two sources: card and maternal recall. Authors also stated that birth weight heaping could happen both from health workers and from mother’s recall side. Exceptionally, the information about birth weight obtained from recall impose bias. Furthermore, it is cross-sectional so that clear temporal associations between the exposure and outcome cannot be established. |
| Bias in selection of the  reported result | Low |  | Authors reported all what was planned in the method, including the statistics mentioned. |
| Overall bias | Serious |  | Outcome ascertainment method and a few confounding could impose bias in outcome measurement and confounding, respectively. |

1. Other health outcomes

**Body weight of adult women**

**Title of the study: Amegah et al. (2019):** Association of biomass fuel use with reduced body weight of adult Ghanaian women

**Step I: review question, potential confounders, co-interventions, and exposure and outcome measurement accuracy information**

| **Step I items** | **Response** |
| --- | --- |
| PECO question | Is there a difference in reduced birth weight risks between HHs using polluting fuels (kerosene, coal/lignite, charcoal, wood, straw/shrubs/grass, agricultural crop and animal dung) Vs HHs using clean fuels (electricity, liquid petroleum gas (LPG), natural gas and biogas, solar energy) for cooking? |
| Confounding for HAP and nutritional problem (birth weight) | **Risk factors for reduced body weight in women**  Height, age, education , marital status, economic status, number of children delivered, use of oral contraceptives, Years of fertile life and Physical activity |
| Co-interventions | - None identified |
| Accuracy of the measurement of exposure to  HAP | The following techniques are placed in descending order of exposure assessment accuracy.   - Biological monitoring - Personal monitoring - Micro-environmental area-based monitoring - Questionnaire/self-report |
| Accuracy of the measurement of outcome (weight) | - Weight scale to the nearest 0.1 Kg to 0.2 Kg, with lighter cloth and no heavy stuffs in the pocket or hand. |

**Step II:** hypothetical target experiment, including specific confounders and co-interventions from that study that will require consideration

1. target randomized trial specific to the study

| Design | Household randomized trial |
| --- | --- |
| Participants | Adult Ghanaian women who were included in Ghana Demographic and Health Survey sample conducted in 2014, and those who were illegible for anthropometric measurements and measured for weight and height. |
| Experimental intervention | HHs using polluting fuels (kerosene, coal/lignite, charcoal, wood, straw/shrubs/grass, agricultural crop and animal dung) |
| Comparator | HHs clean fuels (electricity, liquid petroleum gas (LPG), natural gas and biogas) |

B1. Specify the outcome

Body weight

B2. If multiple outcomes presented, specify the numerical result being assessed

| **Confounding domains listed in step I** | | | |
| --- | --- | --- | --- |
| Confounding domain | Measured variable (s) | Is there evidence that controlling for this Variable was unnecessary? | Is the confounding domain measured validly and reliably by this Variable (or these variables)? |
| Height, age, education , marital status, economic status, number of children delivered, use of oral contraceptives, Years of fertile life and Physical activity | Height | No | No information |
| **Additional confounding domains relevant to the setting of this particular study, or which the study authors identified as important** | | | |
| Palce of residence, religion, ethnicity, and occupation | None | No | Yes |

Step III: RoB across seven items

| **Rec. No. EndNote_17** | | | |
| --- | --- | --- | --- |
| **Bias items** | **Risk of**  **bias** | **Direction of bias** | **Rationale** |
| Bias due to confounding | Moderate |  | Authors controlled place of residence, age, marital status, religion, ethnicity, education level, wealth status and occupation of the mother. Reproductive factors were not considered in the confounding domain. |
| Bias in selection of participants into the study | low |  | DHS implements probability sampling technique (selection of subjects was unrelated to either exposure or outcome status)  There is no means to commit bias in selection of participants. |
| Bias in classification of  exposures | Moderate |  | Authors analysed the independent effect of charcoal, firewood and other biomass (straw/shrubs/grass/agricultural crop) on adult women’s weight.  There might me a case in which households could use a combination of polluting and clean fuels and information was only collected on primary fuel use in the survey. This study also did not account for past exposure to cooking fuel or recent changes in cooking methods. |
| Bias due to deviations from intended exposures | Moderate |  | Survey captured information present only at the time of data collection (cross-sectional); there could be fuel use/choice pattern shift among households. Different studies portrayed that when households economically become stronger; they tend to shift to cleaner fuel.  However, the trend to shift in to cleaner fuel such as clean gases and electricity is minimal and very slow. |
| Bias due to missing data | Low |  | The study is based on nationally representative sample with 97.8% response rate. Furthermore, it is unlikely that missing information is vary between the two (HHs with polluting fuel and HHs with clean fuel) groups. |
| Bias in measurement of  the outcome | Moderate |  | Authors measured weight using a SECA 878 digital scale. The weighing scale has a 200 kg capacity and weighs in 0.01 kg increments. In addition, BMI was calculated from the height measured to the nearest 0.1 cm and weight.  Furthermore, it is cross-sectional so that clear temporal associations between the exposure and outcome cannot be established. |
| Bias in selection of the  reported result | Low |  | Authors reported all what was planned in the method, including the statistics mentioned. |
| Overall bias | Moderate |  | Exposure category and outcome measurement techniques made with lower risk of bias and authors controlled some important variables in their analysis |

**RoB assessment table: Pregnancy and Birth complication**

**Step I: review question, potential confounders, co-interventions, and exposure and outcome measurement accuracy information**

| **Step I items** | **Response** |
| --- | --- |
| PECO question | Is there a difference in reduced birth weight risks between HHs using polluting fuels (kerosene, coal/lignite, charcoal, wood, straw/shrubs/grass, agricultural crop and animal dung) Vs HHs using clean fuels (electricity, liquid petroleum gas (LPG), natural gas and biogas, solar energy) for cooking? |
| Confounding for HAP and nutritional problem (birth weight) | **Risk factors for elevated blood pressure in women**  **Family history, age, race, chronic kidney disease**, diabetes, lack of physical activity, an unhealthy diet (especially high sodium content food), being overweight or obese, drinking too much alcohol, high cholesterol, smoking and tobacco use and stress |
| Co-interventions | - None identified |
| Accuracy of the measurement of exposure to  HAP | The following techniques are placed in descending order of exposure assessment accuracy.   - Biological monitoring - Personal monitoring - Micro-environmental area-based monitoring - Questionnaire/self-report |
| Accuracy of the measurement of outcome (weight at birth) | - Systolic BP (SBP) and diastolic BP (DBP). The skills of the person measuring BP is determinant for accurate result (positioning the patient, selecting the appropriate size cuff, obtaining a valid and reliable measurement and recording the measurement accurately) |

Title of the study: **Arku et al. (2018):** Elevated blood pressure and household solid fuel use in premenopausal women: Analysis of 12 Demographic and Health Surveys (DHS) from 10 countries

**Step II:** hypothetical target experiment, including specific confounders and co-interventions from that study that will require consideration

1. target randomized trial specific to the study

| Design | Household randomized trial |
| --- | --- |
| Participants | Nationally representative premenopausal women from Albania, Armenia, Azerbaijan, Bangladesh, Benin, Ghana, Kyrgyzstan, Lesotho, Namibia, and Peru that were included in DHS conducted between 2005 to 2014 |
| Experimental intervention | HHs using polluting fuels (kerosene, coal/lignite, charcoal, wood, straw/shrubs/grass, agricultural crop and animal dung) |
| Comparator | HHs clean fuels (electricity, liquid petroleum gas (LPG), natural gas and biogas) |

B1. Specify the outcome

Average SBP & DBP and hypertension

B2. If multiple outcomes presented, specify the numerical result being assessed

| **Confounding domains listed in step I** | | | |
| --- | --- | --- | --- |
| Confounding domain | Measured variable (s) | Is there evidence that controlling for this Variable was unnecessary? | Is the confounding domain measured  validly and reliably by this Variable (or these variables)? |
| **Family history, age, race, chronic kidney disease**, diabetes, lack of physical activity, an unhealthy diet (especially high sodium content food), being overweight or obese, drinking too much alcohol, high cholesterol, smoking and tobacco use and stress | Maternal BMI | No | No information |
| **Additional confounding domains relevant to the setting of this particular study, or which the study authors identified as important** | | | |
| education, occupation, wealth index, place of residence (urban, rural), and month of interview | None | No | Yes |

Step III: RoB across seven items

| **Rec. No. EndNote_110** | | | |
| --- | --- | --- | --- |
| **Bias items** | **Risk of**  **bias** | **Direction of bias** | **Rationale** |
| Bias due to confounding | Moderate |  | Authors controlled the following variables in their analysis: individual level factors: age (in years; continuous), body mass index [(BMI) kg/m2; continuous), ethnicity, education, occupation, wealth index, place of residence (urban, rural), and calendar month of interview. They excluded tobacco smoking and alcohol consumption because it is too small frequency in 8 countries and information not exist for two countries. |
| Bias in selection of participants into the study | low |  | DHS implements probability sampling technique (selection of subjects was unrelated to either exposure or outcome status)  There is no means to commit bias in selection of participants. |
| Bias in classification of  exposures | Moderate |  | Authors considered HHs using Solid fuels (coal, charcoal, wood, animal dung, agriculture/crop products, and shrub/grass) as exposed group and excluded households that relied primarily on kerosene for  cooking (< 1%), out of their analysis. However, there may be another misclassification in categorising participants into exposed and non-exposed because some households could use a combination of polluting and clean fuels and information was only collected on primary fuel use in the survey.  This study also did not account for past exposure to cooking fuel or recent changes in cooking methods. |
| Bias due to deviations from intended exposures | Moderate |  | Survey captured information present only at the time of data collection (cross-sectional); there could be fuel use/choice pattern shift among households. Different studies portrayed that when households economically become stronger; they tend to shift to cleaner fuel.  However, the trend to shift in to cleaner fuel such as clean gases and electricity is minimal and very slow. |
| Bias due to missing data | Low |  | The study is based on nationally representative sample of 10 countries, which usually is large adequate. Furthermore, it is unlikely that missing information is vary between the two (HHs with polluting fuel and HHs with clean fuel) groups. |
| Bias in measurement of  the outcome | Moderate |  | Authors reported that trained interviewers collected three BP measurements using fully automatic digital BP measuring devices with automatic upper-arm inflation and pressure release. The average of the last two measurements was used to estimate participant's systolic and diastolic BP.  On the other hand, it is cross-sectional so that clear temporal associations between the exposure and outcome cannot be established. |
| Bias in selection of the  reported result | Low |  | Authors reported all what was planned in the method, including the statistics mentioned. |
| Overall bias | Moderate |  | The analysis is based on large sample and the finding showed large effect size |

**Title of the study: Agrawal et al. (2015):** Effect of Indoor air pollution from biomass and solid fuel combustion on symptoms of preeclampsia/eclampsia in Indian women

**Step I: review question, potential confounders, co-interventions, and exposure and outcome measurement accuracy information**

| **Step I items** | **Response** |
| --- | --- |
| PECO question | Is there a difference in reduced birth weight risks between HHs using polluting fuels (kerosene, coal/lignite, charcoal, wood, straw/shrubs/grass, agricultural crop and animal dung) Vs HHs using clean fuels (electricity, liquid petroleum gas (LPG), natural gas and biogas, solar energy) for cooking? |
| Confounding for HAP and nutritional problem (birth weight) | **Risk factors for eclampsia/preeclampsia**  Family history of preeclampsia, **age (being <15 and >35), chronic kidney/cv disease,** history of chronic hypertension, being overweight or obese, diabetes (gestational), parity (nulliparity), urinary tract infection, severe anaemia, income, multiple gestations and ethnicity |
| Co-interventions | - None identified |
| Accuracy of the measurement of exposure to  HAP | The following techniques are placed in descending order of exposure assessment accuracy.   - Biological monitoring - Personal monitoring - Micro-environmental area-based monitoring - Questionnaire/self-report |
| Accuracy of the measurement of outcome (weight at birth) | - Measuring blood pressure and test urine for protein - Sign and symptom (Swelling, particularly of the arms, hands, or face that is reflected in greater than expected weight gain, which is a result of retaining fluid. **Eclampsia** is diagnosed when a woman with preeclampsia has seizures |

**Step II:** hypothetical target experiment, including specific confounders and co-interventions from that study that will require consideration

1. target randomized trial specific to the study

| Design | Household randomized trial |
| --- | --- |
| Participants | Nationally representative Indian women aged 15-49 years and who had live birth in the last five years presiding Indian’s 2005–2006 NFHS |
| Experimental intervention | HHs using polluting fuels (kerosene, coal/lignite, charcoal, wood, straw/shrubs/grass, agricultural crop and animal dung) |
| Comparator | HHs clean fuels (electricity, liquid petroleum gas (LPG), natural gas and biogas) |

B1. Specify the outcome

Average SBP & DBP and hypertension

B2. If multiple outcomes presented, specify the numerical result being assessed

| **Confounding domains listed in step I** | | | |
| --- | --- | --- | --- |
| Confounding domain | Measured variable (s) | Is there evidence that controlling for this Variable was unnecessary? | Is the confounding domain measured  validly and reliably by this Variable (or these variables)? |
| Family history of preeclampsia, **age (being <15 and >35), chronic kidney/cv disease,** history of chronic hypertension, being overweight or obese, diabetes (gestational), parity (nulliparity), urinary tract infection, severe anaemia, income, multiple gestations and ethnicity | Maternal BMI | No | No information |
| **Additional confounding domains relevant to the setting of this particular study, or which the study authors identified as important** | | | |
| Total children ever born, pregnancy termination history, tobacco & alcohol consumption status, asthma, education, religion, place of residence & region | None | No | Yes |

Step III: RoB across seven items

| **Rec. No. EndNote_323** | | | |
| --- | --- | --- | --- |
| **Bias items** | **Risk of**  **bias** | **Direction of bias** | **Rationale** |
| Bias due to confounding | Low |  | Authors controlled total children ever born, type of pregnancy (twin or singleton), pregnancy termination history, BMI, tobacco & alcohol consumption status, diabetes, asthma, anaemia level, age, education, religion, caste/tribe, wealth index, place of residence & region. |
| Bias in selection of participants into the study | low |  | DHS implements probability sampling technique (selection of subjects was unrelated to either exposure or outcome status)  There is no means to commit bias in selection of participants. |
| Bias in classification of  exposures | Serious | Towards the null | Low-exposure group or HHs using fuels such as kerosene, LPG/natural gas, biogas, or electricity were considered as a comparator group for HHs using either biomass fuels such as wood, straw/shrubs/grass, agricultural crop waste, dung cakes, or other solid fuels such as coal/ lignite and charcoal. Here kerosene categorised as comparator or cleaner fuel group. On top of this, there may be another misclassification in categorising participants into exposed and non-exposed because some households could use a combination of polluting and clean fuels and information was only collected on primary fuel use in the survey.  This study also did not account for past exposure to cooking fuel or recent changes in cooking methods. |
| Bias due to deviations from intended exposures | Moderate |  | Survey captured information present only at the time of data collection (cross-sectional); there could be fuel use/choice pattern shift among households. Different studies portrayed that when households economically become stronger; they tend to shift to cleaner fuel.  However, the trend to shift in to cleaner fuel such as clean gases and electricity is minimal and very slow. |
| Bias due to missing data | Low |  | The study is based on nationally representative sample with 98% response rate.  Furthermore, it is unlikely that missing information is vary between the two (HHs with polluting fuel and HHs with clean fuel) groups. |
| Bias in measurement of  the outcome | Serious |  | Authors used the response obtained from mothers on their last pregnancy whether they experienced symptoms of convulsions (not from fever), swelling of the legs, body or face, excessive fatigue, have difficulty with vision during daylight, or vaginal bleeding. There could be bias either due to recall or due to incorrect characterization of the case. In addition, it is cross-sectional so that clear temporal associations between the exposure and outcome cannot be established. |
| Bias in selection of the  reported result | Low |  | Authors reported all what was planned in the method, including the statistics mentioned. |
| Overall bias | Serious |  | Exposure category and outcome ascertainment are sources of bias. |

Tuberculosis

**Title of the study:** **Mishra et al. (1999):** Biomass Cooking Fuels and Prevalence of Tuberculosis in India

**Step I:** review question, potential confounders, co-interventions, and exposure and outcome measurement accuracy information

| **Step I items** | **Response** |
| --- | --- |
| PECO question | Is there a difference in risk of acquiring TB for 20 and above years age persons living in households practicing indoor combustion of polluting fuel (wood, charcoal, coal, animal dung, plant residue, crop waste and kerosene) vs. the counterparts from households using clean fuel (Liquefied Petroleum Gas, electricity, biogas and solar energy?) |
| Confounding for HAP and child TB | - HIV infection status - Nutritional status (under nourished, low body weight) - People with chronic conditions such as diabetes or kidney disease - Substance abuse (including alcohol consumption, cigarette smoking and other drugs that can hinder abuser’s immune system) - crowding (number of occupants per house/room) - contact with TB case - age - occupation - other medical conditions (diabetes mellitus, silicosis, prolonged therapy with corticosteroids, immunosuppressive therapy, head and neck cancers, severe kidney disease and certain intestinal conditions) |
| Co-interventions | - None identified |
| Accuracy of the measurement of exposure to  HAP | The following techniques are placed in descending order of exposure assessment accuracy.   - Biological monitoring - Personal monitoring - Micro-environmental area-based monitoring - Questionnaire/self-report |
| Accuracy of the measurement of outcome (TB) | The following diagnostic method can be used to identify TB cases:   - [Symptoms](https://www.nationaljewish.org/conditions/tuberculosis-tb/symptoms) - [History of exposure](https://www.nationaljewish.org/conditions/tuberculosis-tb/diagnosis#history) - [Tuberculin skin test](https://www.nationaljewish.org/conditions/tuberculosis-tb/diagnosis#tuberculin_skin_test) or [blood test](https://www.nationaljewish.org/conditions/tuberculosis-tb/diagnosis#blood) (a positive TB skin test or TB blood test only tells that a person has been infected with TB bacteria. Other tests, such as a chest x-ray and a sample of sputum, are needed to see whether the person has TB disease) - [Chest X-ray or chest CT scan](https://www.nationaljewish.org/conditions/tuberculosis-tb/diagnosis#chest_x-ray) - [Sputum test](https://www.nationaljewish.org/conditions/tuberculosis-tb/diagnosis#sputum_test) |

**Step II:** hypothetical target experiment, including specific confounders and co-interventions from the study that will require consideration

1. target randomized trial specific to the study

| Design | Individual randomized trial |
| --- | --- |
| Participants | Nationally representative Indian people aged 20 years old and above included in India’s 1992–1993 National Family Health Survey (NFHS). |
| Experimental intervention | polluting fuels (kerosene, coal/lignite, charcoal, wood, straw/shrubs/grass, agricultural crop and animal dung) for cooking |
| Comparator | clean fuels (electricity, liquid petroleum gas (LPG), solar energy, natural gas and biogas) for cooking |

B1. Specify the outcome

TB in adults

B2. If multiple outcomes presented, specify the numerical result being assessed

| **Confounding domains listed in step I** | | | |
| --- | --- | --- | --- |
| Confounding domain | Measured variable (s) | Is there evidence that controlling for this Variable was unnecessary? | Is the confounding domain measured validly and reliably by this Variable (or these variables)? |
| HIV infection status, nutritional status (being low body weight), existing health condition (chronic diseases such as diabete, kidney and cancer), smoking, crowding, occupation and age | None | No | No information |
| **Additional confounding domains relevant to the setting of this particular study, or which the study authors identified as important** | | | |
| separate kitchen, house type (construction material), gender, education, religion, tribe, residence and geographic region | None | No | Yes |

**Step III: RoB across seven items**

| **Rec. No. EndNote_2725** | | | |
| --- | --- | --- | --- |
| **Bias items** | **Risk of bias** | **Direction of bias** | **Rationale** |
| Bias due to confounder | critical |  | HIV infection status, nutritional status (being low body weight), existing health condition (chronic diseases such as diabete, kidney and cancer), smoking, crowding, occupation and age are risk factors for TB. However, of these variables, authors controlled only age of the respondents and crowding status. |
| Bias in selection of participants into the study | low |  | DHS implements probability sampling technique (selection of subjects was unrelated to either exposure or outcome status)  There is no means to commit bias in selection of participants. |
| Bias in classification of exposures | Critical |  | Authors classify HHs in to two groups: HHs using biomass fuel (wood and dung) as polluting fuel users or exposed group and HHs using fuels such as coal/coke/lignite, charcoal, kerosene, electricity, liquefied petroleum gas, or biogas as cleaner fuel users. They considered polluting fuels like kerosene, charcoal and coal under clean fuel category.  Again, there may be misclassification in categorizing participants into exposed to unclean fuel or not because some households could use a combination of unclean and clean fuels and information was only collected on primary fuel use in NFHS survey. The survey also did not account for past exposure to cooking fuel or recent changes in cooking methods. On the other hand, authors stated that households in India typically use a combination of biomass and cleaner fuels so that the estimated effects of biomass fuels are likely to be reduced |
| Bias due to deviations from intended exposures | Serious |  | Even though there is high chance of exposure status change among participants, information was measured cross-sectional and analyzed from single measurement/interview. However, there is a chance of neighborhood and ambient air pollution from a household using polluted fuel and people might have used combination of solid fuels and cleaner fuel for cooking. Authors excluded “other fuels” category that was 2% of the total HHs, but it might not be unique to specific group. |
| Bias due to missing data | Low |  | It is nationally representative data with very few missing information |
| Bias in measurement of the outcome | Serious |  | It is subjective and ascertained based on the response by the household head or other knowledgeable adult in the household to the question “Does anyone listed suffer from tuberculosis?”. Then, authors in their analysis included a dummy variable with value 1 if a person suffers from active tuberculosis and 0 otherwise. |
| Bias in selection of the reported result | Low |  | It is no way to report/present the report selectively. Statistics described under the method also reported in the result. Full data can be found in the two countries final NFHS report. |
| Overall bias | Critical |  | Because authors miss classified the exposure and failed to control critical confounders. |

**Asthma**

**Title of the study:** **Mishra (2003):** Effect of Indoor Air Pollution from Biomass Combustion on Prevalence of Asthma in the Elderly

**Step I:** review question, potential confounders, co-interventions, and exposure and outcome measurement accuracy information

| **Step I items** | **Response** |
| --- | --- |
| PECO question | Is there a difference in risk of acquiring Asthma for 60 and above years age persons living in households practicing indoor combustion of polluting fuel (wood, charcoal, coal, animal dung, plant residue, crop waste and kerosene) vs. the counterparts from households using clean fuel (Liquefied Petroleum Gas, electricity, biogas and solar energy?) |
| Confounding for HAP and child TB | - Genetics or family history of asthma - Smoking (cigarette smoke irritates the airways. Smokers have a high risk of asthma). - having allergies or other allergy-related conditions - **Viral respiratory infections** (respiratory problems during infancy and childhood can cause chronic asthma) - **Air Pollution** (exposure to the main component of smog (ozone) raises the risk for asthma. those who grew up or live in urban areas have a higher risk for asthma). - **Obesity** (adults who are [overweight or obese are at a greater risk of asthma](http://www.lung.org/about-us/blog/2016/07/the-link-between-asthma-weight.html)). |
| Co-interventions | - None identified |
| Accuracy of the measurement of exposure to  HAP | The following techniques are placed in descending order of exposure assessment accuracy.   - Biological monitoring - Personal monitoring - Micro-environmental area-based monitoring - Questionnaire/self-report |
| Accuracy of the measurement of outcome (Asthma) | These are type of asthma measurement:   - Assessing health history - Testing for asthma (it can be lung function test or/and   allergy test (blood or skin)) |

**Step II:** hypothetical target experiment, including specific confounders and co-interventions from the study that will require consideration

1. target randomized trial specific to the study

| Design | Individual randomized trial |
| --- | --- |
| Participants | Nationally representative Indian people aged 60 years old and above included in India’s 1998-9 National Family Health Survey (NFHS). |
| Experimental intervention | polluting fuels (kerosene, coal/lignite, charcoal, wood, straw/shrubs/grass, agricultural crop and animal dung) for cooking |
| Comparator | clean fuels (electricity, liquid petroleum gas (LPG), solar energy, natural gas and biogas) for cooking |

B1. Specify the outcome

Asthma

B2. If multiple outcomes presented, specify the numerical result being assessed

| **Confounding domains listed in step I** | | | |
| --- | --- | --- | --- |
| Confounding domain | Measured variable (s) | Is there evidence that  controlling for this Variable was unnecessary? | Is the confounding domain measured validly and reliably by this Variable (or these variables)? |
| Genetics or family history of asthma, Smoking status, having allergies, **Viral respiratory infections, air Pollution** (exposure to the main component of smog (ozone) and **Obesity** | None | No | No information |
| **Additional confounding domains relevant to the setting of this particular study, or which the study authors identified as important** | | | |
| age, sex, marital status, education, religion of household head, caste/tribe of household head, house type, availability of a separate kitchen in the house, crowding in the household, living standard of the household, urban/rural residence, and geographic region. | None | No | Yes |

**Step III: RoB across seven items**

| **Rec. No. EndNote_821** | | | |
| --- | --- | --- | --- |
| **Bias items** | **Risk of bias** | **Direction of bias** | **Rationale** |
| Bias due to confounder | critical |  | Authors controlled smoking status (both active and passive), age, sex, marital status, education, religion of household head, caste/tribe of household head, house type, availability of a separate kitchen in the house, crowding in the household, living standard of the household, urban/rural residence, and geographic region. However, other important asthma risk factors such as genetics or family history of asthma, having allergies, **viral respiratory infections, air pollution** (exposure to the main component of smog (ozone) and **obesity status of the respondents were not examined.** |
| Bias in selection of participants into the study | low |  | DHS implements probability sampling technique (selection of subjects was unrelated to either exposure or outcome status)  There is no means to commit bias in selection of participants. |
| Bias in classification of exposures | Critical |  | Authors classify HHs in to three groups: high-exposure group (households using only biomass fuels: wood, crop residues, or dung cakes), low-exposure group (households using only cleaner fuels: kerosene, petroleum gas, biogas, or electricity), and medium-exposure group (a mix of biomass fuels and cleaner fuels or coal/coke/lignite/charcoal). They created these group from multiple response question which could be the strength of this study. However, they considered kerosene under cleaner fuel category, which is the source of exposure classification bias. Again, the survey also did not account for past exposure to cooking fuel or recent changes in cooking methods. |
| Bias due to deviations from intended exposures | Serious |  | Even though there is high chance of exposure status change among participants, information was measured cross-sectional and analyzed from single measurement/interview. |
| Bias due to missing data | Low |  | It is nationally representative data with very few missing information (98% response rate) |
| Serious | Moderate |  | It is subjective and ascertained based on the response by the household head or other knowledgeable adult in the household to the question “Does anyone listed suffer from Asthma?” Then, authors in their analysis included a dummy variable with value 1 if a person suffers from active tuberculosis and 0 otherwise. Authors declared that there is a possibility of over reporting because some other disease conditions with similar symptoms, such as chronic bronchitis or chronic obstructive pulmonary disease, may be reported as asthma. They also stated that it is assessed among elder people so that under reporting is minimal because the stigma is for children and young adults. |
| Bias in selection of the reported result | Low |  | It is no way to report/present the report selectively. Statistics described under the method also reported in the result. Full data can be found in the two countries final Indian NFHS report. |
| Overall bias | Critical |  | Because authors miss classified the exposure and failed to control critical confounders. |

**Title of the study:** **Agrawal (2012):** Effect of Indoor Air Pollution from Biomass and Solid Fuel Combustion on Prevalence of Self-Reported Asthma among Adult Men and Women in India: Findings from a Nationwide Large-Scale Cross-Sectional Survey

**Step I:** review question, potential confounders, co-interventions, and exposure and outcome measurement accuracy information

| **Step I items** | **Response** |
| --- | --- |
| PECO question | Is there a difference in risk of acquiring Asthma for men and women adults aged between 20 & 49 living in households practicing indoor combustion of polluting fuel (wood, charcoal, coal, animal dung, plant residue, crop waste and kerosene) vs. the counterparts from households using clean fuel (Liquefied Petroleum Gas, electricity, biogas and solar energy?) |
| Confounding for HAP and child TB | - Genetics or family history of asthma - Smoking (cigarette smoke irritates the airways. Smokers have a high risk of asthma). - having allergies or other allergy-related conditions - **Viral respiratory infections** (respiratory problems during infancy and childhood can cause chronic asthma) - **Air Pollution** (exposure to the main component of smog (ozone) raises the risk for asthma. those who grew up or live in urban areas have a higher risk for asthma). - **Obesity** (adults who are [overweight or obese are at a greater risk of asthma](http://www.lung.org/about-us/blog/2016/07/the-link-between-asthma-weight.html)). |
| Co-interventions | - None identified |
| Accuracy of the measurement of exposure to  HAP | The following techniques are placed in descending order of exposure assessment accuracy.   - Biological monitoring - Personal monitoring - Micro-environmental area-based monitoring - Questionnaire/self-report |
| Accuracy of the measurement of outcome (Asthma) | These are type of asthma measurement:   - Assessing health history - Testing for asthma (it can be lung function test or/and   allergy test (blood or skin) |

**Step II:** hypothetical target experiment, including specific confounders and co-interventions from the study that will require consideration

1. target randomized trial specific to the study

| Design | Individual randomized trial |
| --- | --- |
| Participants | Nationally representative Indian adult men and women aged between 20 and 49 years included in India’s third National Family Health Survey conducted in 2005–2006. |
| Experimental intervention | polluting fuels (kerosene, coal/lignite, charcoal, wood, straw/shrubs/grass, agricultural crop and animal dung) for cooking |
| Comparator | clean fuels (electricity, liquid petroleum gas (LPG), solar energy, natural gas and biogas) for cooking |

B1. Specify the outcome

Asthma

B2. If multiple outcomes presented, specify the numerical result being assessed

| **Confounding domains listed in step I** | | | |
| --- | --- | --- | --- |
| Confounding domain | Measured variable (s) | Is there evidence that  controlling for this Variable was unnecessary? | Is the confounding domain measured validly and reliably by this Variable (or these variables)? |
| Genetics or family history of asthma, Smoking status, having allergies, **Viral respiratory infections, air Pollution** (exposure to the main component of smog (ozone) and **Obesity** | None | No | No information |
| **Additional confounding domains relevant to the setting of this particular study, or which the study authors identified as important** | | | |
| age, marital status, education, religion, ethnicity, house type, cooking place, crowding, HH structure, wealth index, residence & region | None | No | Yes |

**Step III: RoB across seven items**

| **Rec. No. EndNote_509** | | | |
| --- | --- | --- | --- |
| **Bias items** | **Risk of bias** | **Direction of bias** | **Rationale** |
| Bias due to confounder | critical |  | Tobacco smoking status, age, marital status, education, religion, ethnicity, house type, cooking place, crowding, HH structure, wealth index, residence & region are controlled as a confounder for self-reported asthma. Some of these variables could be risk factors for but other important asthma risk factors such as genetics or family history of asthma, having allergies, **viral respiratory infections, air pollution** (exposure to the main component of smog (ozone) and **obesity status of the respondents were not examined.** |
| Bias in selection of participants into the study | low |  | DHS implements probability sampling technique (selection of subjects was unrelated to either exposure or outcome status)  There is no means to commit bias in selection of participants. |
| Bias in classification of exposures | Serious | Towards the null | Authors classify HHs in to two groups: 1. HHs using biomass fuel (wood, crop residues, or dung cakes) and solid fuel (coal/lignite and charcoal) and 2. HHs using cleaner fuels (kerosene, petroleum gas, biogas, or electricity). According to this category, kerosene is grouped under cleaner fuel while it is highly polluted, which is the source of exposure classification bias. Again, the survey also did not account for past exposure to cooking fuel or recent changes in cooking methods. |
| Bias due to deviations from intended exposures | Serious |  | In addition to the bias made in categorizing fuel in to cleaner and polluted fuel (inter-mixing), there could be probability of switching fuel from one for to another. Furthermore, information was measured cross-sectional and analyzed from single measurement/interview. |
| Bias due to missing data | Low |  | It is nationally representative data with very few missing information (98% response rate) |
| Serious | Moderate |  | It is subjective and ascertained based on the response by the household head or other knowledgeable adult in the household to the question “Does anyone listed suffer from Asthma?” Then, authors in their analysis included a dummy variable with value 1 if a person suffers from active tuberculosis and 0 otherwise. Authors declared that there is a possibility of over reporting because some other disease conditions with similar symptoms, such as chronic bronchitis or chronic obstructive pulmonary disease, may be reported as asthma. They also stated that it is assessed among elder people so that under reporting is minimal because the stigma is for children and young adults. |
| Bias in selection of the reported result | Low |  | It is no way to report/present the report selectively. Statistics described under the method also reported in the result. Full data can be found in the two countries final Indian NFHS report. |
| Overall bias | Critical |  | Because authors miss classified the exposure and failed to control critical confounders. |

**Reference**

1. Morgan RL, Thayer KA, Santesso N, Holloway AC, Blain R, Eftim SE, et al. A risk of bias instrument for non-randomized studies of exposures: A users' guide to its application in the context of GRADE. Environ Int. 2019; 122:168-84.
