# Supplementary material for: A Systematic Review and Appraisal of Epidemiological Studies on Household Fuel Use and Its Health Effects Using Demographic and Health Surveys
Source: Int J Environ Res Public Health. 2021 Feb 3;18(4):1411. doi: 10.3390/ijerph18041411 (PMC7913474; doi:10.3390/ijerph18041411)
Supplement: Supplementary file 1 [file ijerph-18-01411-s001.zip › Supplementary files/Table S3_Quality and Risk of Bias results heat map.docx]

**S3 Table: Risk of bias judgement**: Based on **Morgan et al. (2019):** A risk of bias instrument for non-randomized studies of exposures tool [1]

1. Two-level (item and study) judgements for **respiratory problem** studies.

| Studies | Confounding | Selection of participants | Classification of exposures | Deviations from intended exposures | Missing data | Measurement of the outcome | Reported result | **Study-level RoB judgement** |
| --- | --- | --- | --- | --- | --- | --- | --- | --- |
| Mondal (2020) |  |  |  |  |  |  |  |  |
| Naz (2020) |  |  |  |  |  |  |  |  |
| Woolley (2020) |  |  |  |  |  |  |  |  |
| Budhathoki (2020) |  |  |  |  |  |  |  |  |
| Rana (2019) |  |  |  |  |  |  |  |  |
| Khan (2018) |  |  |  |  |  |  |  |  |
| Capuno (2018) |  |  |  |  |  |  |  |  |
| Akinyemi (2018) |  |  |  |  |  |  |  |  |
| Khan (2017) |  |  |  |  |  |  |  |  |
| Daniel (2016) |  |  |  |  |  |  |  |  |
| Wichmann (2015) |  |  |  |  |  |  |  |  |
| Buchner (2015) |  |  |  |  |  |  |  |  |
| Acharya (2015) |  |  |  |  |  |  |  |  |
| Patel (2013) |  |  |  |  |  |  |  |  |
| Kilabuko (2007) |  |  |  |  |  |  |  |  |
| Mishra (2005) |  |  |  |  |  |  |  |  |
| Mishra (2003) |  |  |  |  |  |  |  |  |
| **Item-level** |  |  |  |  |  |  |  |  |

| low | moderate | serious | critical |
| --- | --- | --- | --- |

1. Two-level (item and study) judgements for **mortality** studies.

| Studies | Confounding | Selection of participants | Classification of exposures | Deviations from intended exposures | Missing data | Measurement of the outcome | Reported result | **Study-level RoB judgement** |
| --- | --- | --- | --- | --- | --- | --- | --- | --- |
| Samuel (2018) |  |  |  |  |  |  |  |  |
| Nisha (2018) |  |  |  |  |  |  |  |  |
| Naz (2018) |  |  |  |  |  |  |  |  |
| Owili (2017) |  |  |  |  |  |  |  |  |
| Naz (2017) |  |  |  |  |  |  |  |  |
| Khan (2017) |  |  |  |  |  |  |  |  |
| Naz (2016) |  |  |  |  |  |  |  |  |
| Akinyemi (2016) |  |  |  |  |  |  |  |  |
| Naz (2015) |  |  |  |  |  |  |  |  |
| Kleimola (2015) |  |  |  |  |  |  |  |  |
| Ezeh (2014) |  |  |  |  |  |  |  |  |
| Pandey (2013) |  |  |  |  |  |  |  |  |
| Epstein (2013) |  |  |  |  |  |  |  |  |
| Wichmann (2006) |  |  |  |  |  |  |  |  |
| **Item-level** |  |  |  |  |  |  |  |  |

| low | moderate | serious | critical |
| --- | --- | --- | --- |

1. Two-level (item and study) judgements for **nutritional problem** studies.

| Studies | Confounding | Selection of participants | Classification of exposures | Deviations from intended exposures | Missing data | Measurement of the outcome | Reported result | **Study-level RoB judgement** |
| --- | --- | --- | --- | --- | --- | --- | --- | --- |
| Dadras (2017) |  |  |  |  |  |  |  |  |
| Machisa (2013) |  |  |  |  |  |  |  |  |
| Kyu (2010) |  |  |  |  |  |  |  |  |
| Kyu (2009) |  |  |  |  |  |  |  |  |
| Mishra (2007) |  |  |  |  |  |  |  |  |
| **Item-level** |  |  |  |  |  |  |  |  |

| low | moderate | serious | critical |
| --- | --- | --- | --- |

1. Two-level (item and study) judgements for **weight related** studies.

| Studies | Confounding | Selection of participants | Classification of exposures | Deviations from intended exposures | Missing data | Measurement of the outcome | Reported result | Study-level RoB judgement |
| --- | --- | --- | --- | --- | --- | --- | --- | --- |
| Milanzi (2017) |  |  |  |  |  |  |  |  |
| Khan (2017) |  |  |  |  |  |  |  |  |
| Epstein (2013) |  |  |  |  |  |  |  |  |
| Sreeramareddy (2011) |  |  |  |  |  |  |  |  |
| Mishra (2004) |  |  |  |  |  |  |  |  |
| **Item-level judgement** |  |  |  |  |  |  |  |  |

| low | moderate | serious | critical |
| --- | --- | --- | --- |

1. Two-level (item and study) judgements for pregnancy and birth complication studies.

| Studies | Confounding | Selection of participants | Classification of exposures | Deviations from intended exposures | Missing data | Measurement of the outcome | Reported result | Study-level RoB judgement |
| --- | --- | --- | --- | --- | --- | --- | --- | --- |
| Nisha (2018) |  |  |  |  |  |  |  |  |
| Khan (2017) |  |  |  |  |  |  |  |  |
| Mishra (2005) |  |  |  |  |  |  |  |  |
| Agrawal (2015) |  |  |  |  |  |  |  |  |
| **Item-level** |  |  |  |  |  |  |  |  |

| low | moderate | serious | critical |
| --- | --- | --- | --- |

1. Two-level (item and study) judgements for other health outcome studies.

| Studies | Confounding | Selection of participants | Classification of exposures | Deviations from intended exposures | Missing data | Measurement of the outcome | Reported result | Study-level RoB judgement |
| --- | --- | --- | --- | --- | --- | --- | --- | --- |
| Amegah (2019) |  |  |  |  |  |  |  |  |
| Arku (2018) |  |  |  |  |  |  |  |  |
| Agrawal (2012) |  |  |  |  |  |  |  |  |
| Mishra (2003) |  |  |  |  |  |  |  |  |
| Mishra (1999) |  |  |  |  |  |  |  |  |
| **Item-level** |  |  |  |  |  |  |  |  |

| low | moderate | serious | critical |
| --- | --- | --- | --- |

**Study/outcome and item- level RoB judgement score**

Study/outcome level results

18/50- Moderate RoB= 36%

16/50- Serious RoB= 32%

16/50- Critical RoB= 32%

**Reference**

1. Morgan RL, Thayer KA, Santesso N, Holloway AC, Blain R, Eftim SE, et al. A risk of bias instrument for non-randomized studies of exposures: A users' guide to its application in the context of GRADE. Environ Int. 2019;122:168-84.
