# Supplementary material for: A Systematic Review and Appraisal of Epidemiological Studies on Household Fuel Use and Its Health Effects Using Demographic and Health Surveys
Source: Int J Environ Res Public Health. 2021 Feb 3;18(4):1411. doi: 10.3390/ijerph18041411 (PMC7913474; doi:10.3390/ijerph18041411)
Supplement: Supplementary file 1 [file ijerph-18-01411-s001.zip › Supplementary files/Table S4_Confounding controled.docx]

**Table S4.** Confounding controlled with their code in the document

| Child related factors | code | Individual/family related factors | Code | Household related factors | Code | Environmental related factors | Code | Health service related factors | Code |
| --- | --- | --- | --- | --- | --- | --- | --- | --- | --- |
| child’s age | 1 | mother’s age | 14 | number of under-five children | 44 | ecological zone | 65 | number of ANC attended | 63 |
| child’s gender | 2 | mother’s education status | 15 | crowding | 45 | survey year | 66 | place of delivery | 64 |
| child’s birth order | 3 | maternal smoking status | 16 | number of sleeping room | 46 | season of interview | 67 |  |  |
| birth weight/birth size | 4 | maternal alcohol consumption status | 17 | cooking location | 47 | geographic location | 68 |  |  |
| Child’s vaccination status | 5 | mother’s body mass index | 18 | stove ventilation | 48 | country | 69 |  |  |
| Child’s breastfeeding status | 6 | Took iron during pregnancy | 19 | Presence of smoker in the household | 49 | Acute respiratory infection status of a child | 70 |  |  |
| child’s nutritional status | 7 | mother’s anaemia status | 20 | place of residence | 50 | diarrhoea status of a child | 71 |  |  |
| parity | 8 | took malaria drug during pregnancy | 21 | region of residence | 51 | fever status of a child | 72 |  |  |
| pregnancy type (single/multiple) | 9 | pregnancy termination history | 22 | Household wealth index | 52 | stunting status of a child | 73 |  |  |
| inter birth interval | 10 | mother’s occupation | 23 | family size | 53 | wasting status of a child | 74 |  |  |
| year of birth | 11 | maternal working status | 24 | drinking water source | 54 | malaria status of a child | 75 |  |  |
| child live with mother | 12 | media exposure | 25 | time to water source | 55 | respondent’s diabetes status | 76 |  |  |
| own child or grandchild | 13 | mother controlled by husband | 26 | latrine status | 56 | respondent’s asthma status | 77 |  |  |
| *Vitamin A supplementation | 1a | mother physically abused by husband | 27 | housing material | 57 | month of interview | 78 |  |  |
| *Mode of delivery | 1b | mother humiliated by husband | 28 | presence of window | 58 | History of TB contact | 78a |  |  |
|  |  | mother’s perception of medical care | 29 | access to electricity | 59 |  |  |  |  |
|  |  | religion | 30 | having health insurance | 60 |  |  |  |  |
|  |  | ethnicity | 31 | utilization of health care service | 61 |  |  |  |  |
|  |  | maternal marital status | 32 | food security | 62 |  |  |  |  |
|  |  | father’s occupation | 33 |  |  |  |  |  |  |
|  |  | father’s education | 34 |  |  |  |  |  |  |
|  |  | father’s age | 35 |  |  |  |  |  |  |
|  |  | father’s smoking status | 36 |  |  |  |  |  |  |
|  |  | gender of household head | 37 |  |  |  |  |  |  |
|  |  | age of household head | 38 |  |  |  |  |  |  |
|  |  | smoking status | 39 |  |  |  |  |  |  |
|  |  | age | 40 |  |  |  |  |  |  |
|  |  | gender | 41 |  |  |  |  |  |  |
|  |  | marital status | 42 |  |  |  |  |  |  |
|  |  | educational status | 43 |  |  |  |  |  |  |

ANC: antenatal care, *from articles obtained in the update search
